# Triplet Energy Transfer as a Handle to Tune 1,2-Dialkyldiazene Fragmentation in Radical C(sp^3^)–C(sp^2^) Cross-Coupling

**DOI:** 10.1021/jacs.5c22244

**Published:** 2026-02-17

**Authors:** Joffrey Scriven, Deepta Chattapadhyay, Felix Glaser, Benjamin Elias, Quentin Michaudel, Ludovic Troian-Gautier

**Affiliations:** † 83415 UCLouvain, Institut de la Matière Condensée et des Nanosciences (IMCN), Molecular Chemistry, Materials and Catalysis (MOST), Place Louis Pasteur 1/L4.01.02, B-1348 Louvain-la-Neuve, Belgium; ‡ Department of Chemistry, Texas A&M University, College Station, Texas 77843, United States; § Department of Materials Science and Engineering, 14736 Texas A&M University, College Station, Texas 77843, United States; ∇ Wel Research Institute, Avenue Pasteur 6, 1300 Wavre, Belgium

## Abstract

Mechanistic investigation of light-induced processes is paramount as it not only offers an overall mechanistic picture but also provides information about the efficiency and associated rate constants of the different reaction steps. In some cases, study of systematic series of photosensitizers or quenchers also allows to determine ground-state redox potentials or triplet energy levels of unknown species. Herein, through a combination of steady-state and time-resolved spectroscopic techniques, we elucidate the mechanism of geminate triplet radical pair formation from 1,2-dialkyldiazenes operating via energy transfer from excited photocatalysts. Stern–Volmer and Rehm–Weller analyses confirmed the energy-transfer pathway and provided access to the triplet energy level of two model 1,2-dialkyldiazenes, which were found to be around 2.3 eV. Further evidence was gained by mediator-enhanced triplet energy transfer to an anthracene substrate, showcasing that the excited diazene can serve as an energy shuttle that can be intercepted before bond fragmentation to release N_2_ and the corresponding radicals. This activation mechanism confers clear advantages over conventional high-energy UV photofragmentation as triplet sensitization was shown to promote a more efficient solvent-cage escape of the resulting geminate radical pairs relative to direct excitation. Additionally, the structure of the 1,2-dialkyldiazenes was found to profoundly influence the kinetics of fragmentation following energy transfer. These mechanistic insights were leveraged to improve C­(sp^3^)–C­(sp^2^) cross-coupling efficiency with challenging electron-rich aryl bromides by slowing alkyl radical generation through photocatalyst selection to match the rate of Ni oxidative addition, thereby demonstrating the tunability of energy-transfer-based dual catalytic systems.

## Graphical abstract

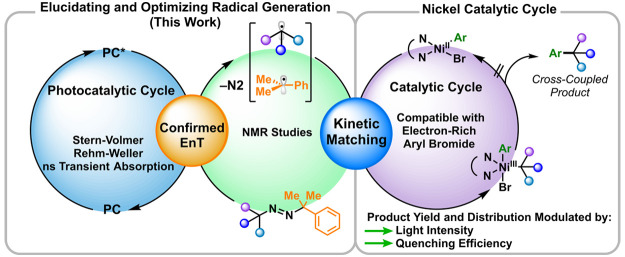

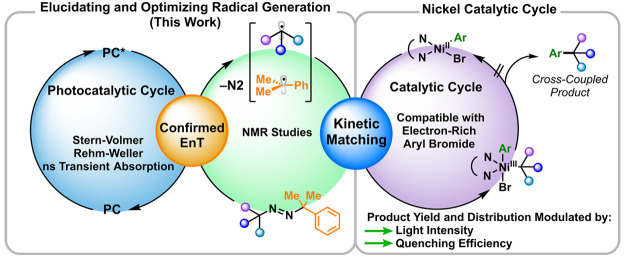

## Introduction

The development of photocatalytic systems, promoting either single-electron transfer or energy transfer processes upon light irradiation, have dramatically reshaped the way chemists approach reaction design in organic synthesis and represents a modular strategy for the late-stage formation of various linkages under mild and selective conditions.^1,2^ While visible-light photocatalysis has recently been leveraged in a myriad of organic transformations,^3–5^ elucidating the mechanistic underpinnings that control the reactivity of photocatalysts (PCs) in these processes remains a grand challenge, which is even more apparent based on the growing complexity of mechanisms using two or three catalytic cycles in combination.^6–10^ Indeed, understanding whether photocatalysts in a given transformation operate through energy transfer (EnT), oxidative or reductive single-electron transfer (SET) or competing nonproductive pathways is crucial for reaction design and process optimization. Metallaphotoredox catalysis, which merges photoredox and transition-metal (TM) catalysis, has dominated the field of radical cross-coupling reactions under visible light.^11^ However, these processes require careful tuning of redox events within a complex mixture of intermediates and often rely on terminal oxidants or reducing agents. In contrast, EnT-based photocatalysis requires only matching the photocatalyst (PC) and substrate to enable exergonic Dexter energy transfer in the case of triplet photosensitizers.^12–14^ Despite their appeal, photocatalytic systems that generate radicals via energy transfer remain underexplored in transition-metal-catalyzed cross-couplings,^15,16^ largely due to the difficulty of distinguishing EnT from single-electron transfer in multimetallic systems.^11,17^ Further understanding of dual catalytic system operating independently would allow the optimization of such methods and subsequent design of unprecedented transformations.

Spectroscopic measurements have proven indispensable in meeting this challenge, allowing researchers to directly probe the short-lived excited states and transient intermediates that dictate photoredox reactivity. While developed decades ago in the context of classical photochemistry, Stern–Volmer quenching experiments, coupled to transient absorption spectroscopy remain among the most prominent approaches to unravel mechanistic details. In Stern–Volmer quenching experiments, scientists can quantify the quenching rate constant (*k*_q_) of any quencher toward the photocatalysts and thus establish whether substrates or additives effectively interact with the photocatalyst’s excited state.^18^ Whereas Stern–Volmer analysis has become a standard diagnostic tool to provide the first experimental evidence of a proposed mechanism, it is unable to provide information about the quenching process, i.e. whether quenching is operating via energy or electron transfer processes. It only provides kinetic information via the determined quenching rate constant.^19^ Transient absorption spectroscopy, albeit a specialized technique, is the technique of choice to establish the quenching process as it allows observation of transient photoproducts formed immediately after the quenching step, with the condition that the cage escape yield is sufficiently large and that the photoproducts absorb in the investigated detection range.^20^ To complete the kinetic information given by the Stern–Volmer analysis, the complementary Rehm–Weller analysis allows to obtain critical thermodynamic insight.^21^ By combining the quenching rate constant with excited-state redox potential or the excited-state energy of a photocatalyst, the Rehm–Weller approach allows to differentiate between energy and electron transfer quenching processes. This approach has been scarcely used in photoredox catalysis, despite its ability to distinguish the often-subtle distinctions between electron transfer and energy transfer chemistry.^22,23^

Recently, the Michaudel group reported a deaminative Ni-catalyzed C­(sp^3^)–C­(sp^2^) cross coupling relying on cumyl-based 1,2-dialkyldiazenes as radical precursors that were activated under blue light irradiation in the presence of Ir­(III) PCs (Figure 1).^24,25^ Primary amines were converted to diazenes through a two-step procedure that included activation via SuFEx click chemistry^26^ followed by the *aza-*Ramberg Bäcklund reaction^27,28^ (Figure 1). The proposed mechanism for the subsequent denitrogenative generation of alkyl radicals and Ni-catalyzed cross-coupling is depicted in Figure 1. Visible light excitation of an Ir­(III) PC leads to a presumed energy transfer to the diazene, triggering its fragmentation and giving rise to an unsymmetrical geminate triplet radical pair that prevents the ultrafast recombination that characterizes singlet pairs produced from direct excitation of 1,2-dialkyldiazenes with UV light.^29–31^ Once the radicals escape the solvent cage and diffuse apart, they adopt different trajectories according to a Ni-promoted “radical sorting” step. Cumyl radical **II**, owing to its planar structure and resonance stabilization, shows little tendency to coordinate with Ni, and instead undergoes dimerization potentially facilitated by π—π interactions.^32^ In contrast, radical **I** can be intercepted through an oxidative ligation step, putatively by a Ni­(II) species **III** and reductive elimination from **IV** then affords the target cross-coupled product. Based on the literature, several pathways can be invoked to close the catalytic cycle including reduction of Ni­(I) **V** to Ni(0) **VI** in a single-electron step followed by oxidative addition with the aryl bromide substrate.^33^ Alternatively, recent studies suggest that oxidative addition could take place at a Ni­(I) center (**V** to **VII**, gray pathway) followed by reduction to **III**, for example through comproportionation.^34–36^ While nickel-based catalytic cycles, particularly in metallaphotoredox systems, have been studied extensively in the literature,^37–43^ examples of cross-couplings in which carbon-centered radicals are generated through photosensitized, redox-neutral pathways from organic precursors remain scarce.^44,45^ In the rare reported examples of radical generation via energy transfer, photosensitization of a metal complex is indeed typically at play rather than substrate sensitization as the one postulated in Figure 1.^2,46,47^ Mechanistic data collected in the seminal publication by Michaudel and co-workers included differences in Stern–Volmer constants (K_SV_) among selected Ir­(III) PCs and cyclic voltammetry measurement that provided mechanistic clues but distinguishing between SET and EnT pathways remained challenging in the absence of kinetic and spectroscopic information from time-resolved spectroscopy.

**Figure 1:**
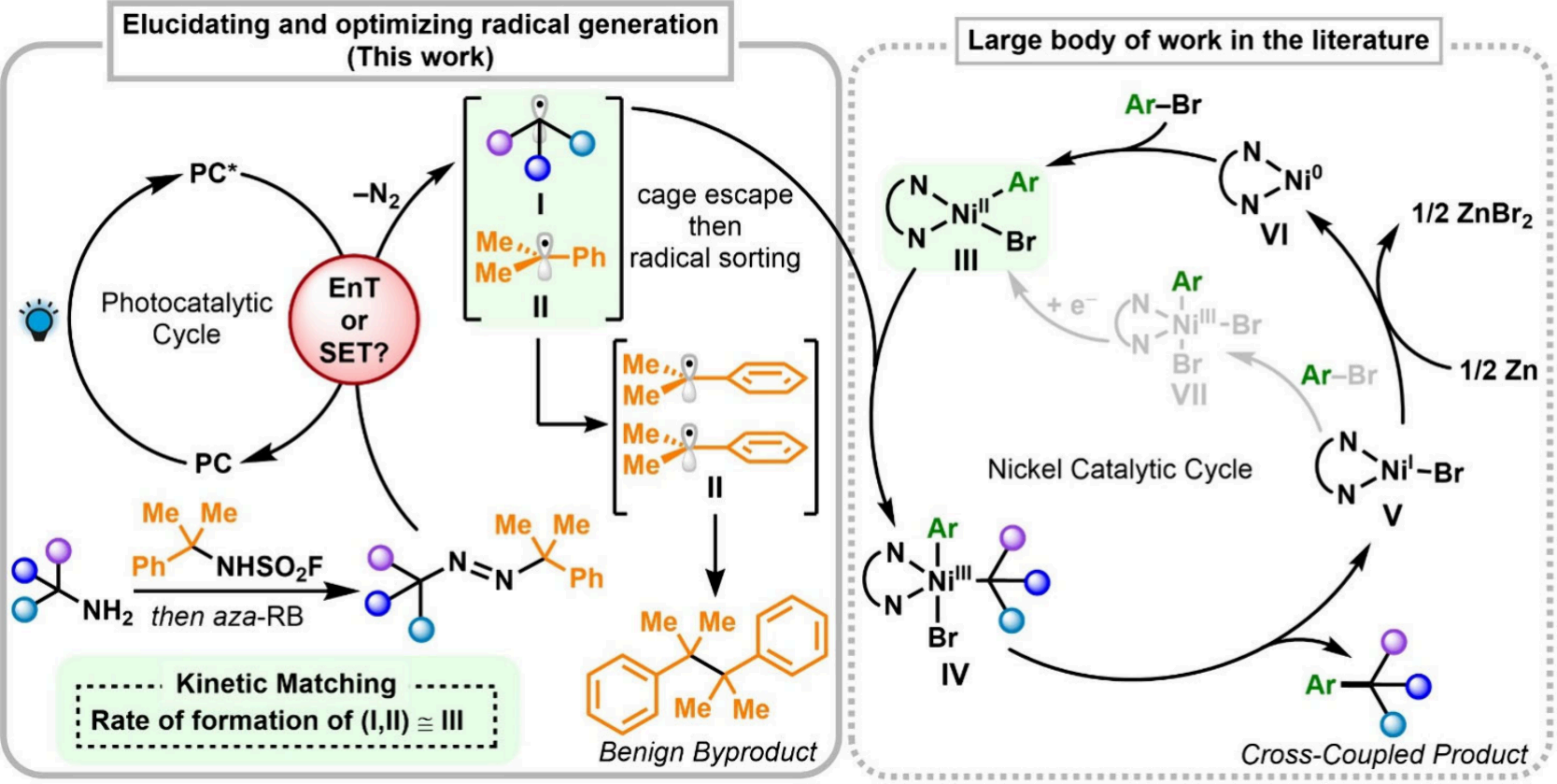
Proposed reaction mechanism showcasing the putative energy transfer step from the excited PC (PC*) to the diazene, leading to fragmentation and the formation of the corresponding geminate radical pair [**I**, **II**]. Following cage escape and radical sorting, desired radical **I** is putatively captured by Ni species **III** to produce the corresponding cross-coupled product and **V** after reductive elimination. Several pathways have been proposed in the literature for the Ni turnover steps as similar Ni catalytic cycles have been investigated (refs 37–43 ). Cumyl radical **II**, likely due to its planar structure and resonance stabilization, shows little tendency to coordinate with Ni, and instead undergoes dimerization. *aza-*RB = *aza*-Ramberg-Bäcklund reaction (ref 25). Note that other routes have been proposed to involve oxidative addition to the Ni­(I) species, as highlighted in gray (refs 34–36 ).

In this study, we set out to determine the triplet energy of the diazene substrates using a series of 15 photocatalysts that span excited-state reduction potential from +0.47 to +2.52 V vs Ag|AgCl and triplet energy that ranges from 1.93 to 3.21 eV. Employing Stern–Volmer and Rehm–Weller analyses, in combination with nanosecond transient absorption spectroscopy, we were able to investigate the rate of quenching over a wide range of driving forces and determine that the triplet energy of the diazene is located around 2.3 eV. This, coupled to diazene-mediated energy transfer to anthracene allowed to unequivocally confirm that the quenching process leading to product formation occurs via excited-state energy transfer. Kinetic analysis of the photofragmentation under varied conditions via ^1^H NMR spectroscopy supported the importance of a triplet-sensitization process to obtain better cage escape. Leveraging these mechanistic insights, we identified improved conditions for the cross-coupling of cumyl-based diazenes, prepared from various amines, with otherwise sluggish electron-rich aryl bromides by finely matching the rates of radical generation and radical capture by the Ni catalyst. Overall, our work highlights the importance of mechanistic understanding to guide the optimization of conditions and yields for photocatalysis.

## Results and Discussion

Rehm–Weller analysis requires the use of a judicious series of PCs or quenchers that spans a large range of redox potentials or triplet energies. As such, we used a series of 15 PCs composed of 7 Ir­(III) complexes, 2 Ru­(II) complexes and 6 organic PCs (Figure 2). These photocatalysts were all available from previous studies^48^ or commercially available. [Ir­(CF_3_pmb)_3_] (**PC-14**) was synthesized as described in the literature.^49^

**Figure 2:**
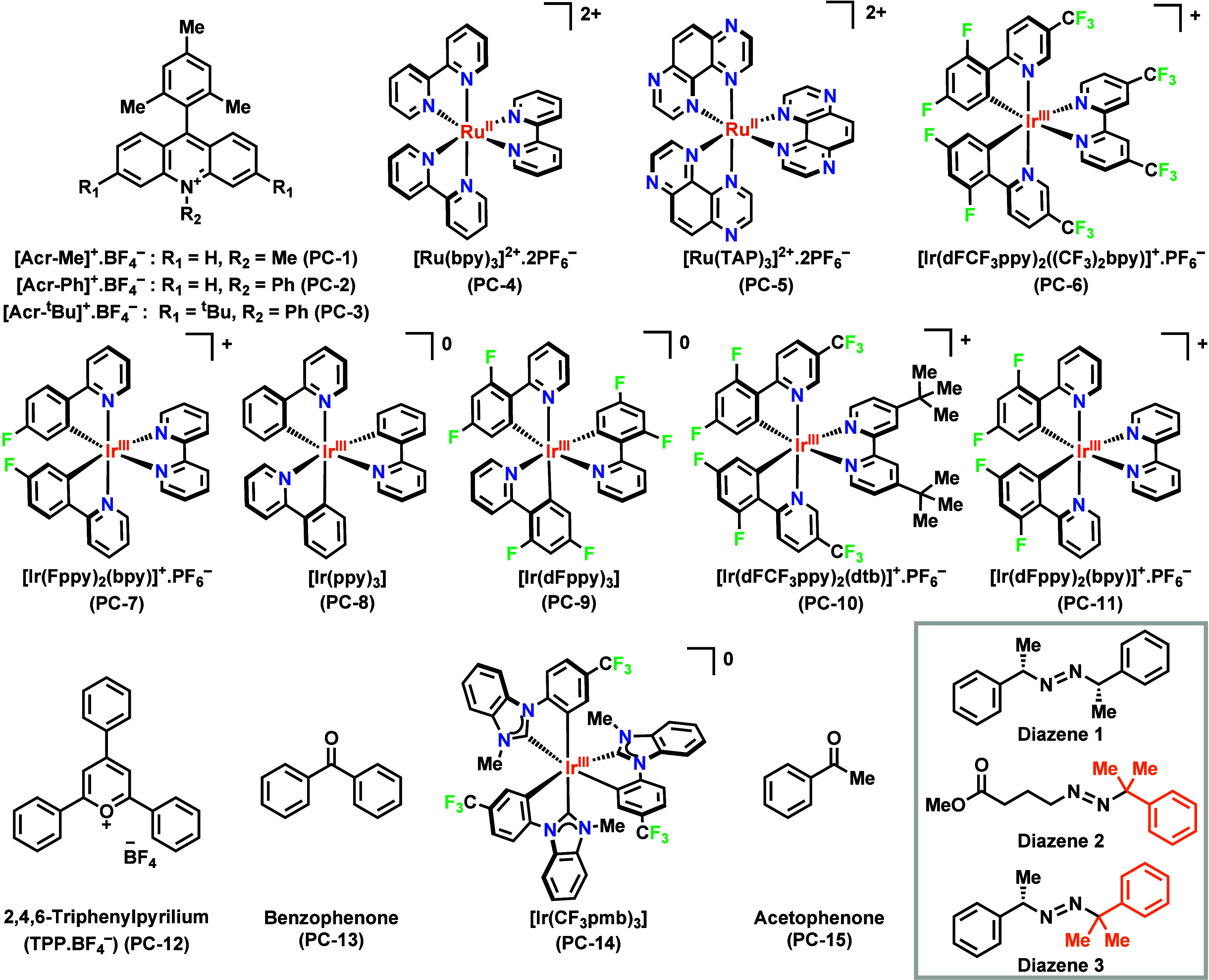
Structure of the 15 photocatalysts and relevant diazenes investigated in the present study. The relevant ground- and excited-state properties of the PCs are compiled in Table 1.

### Ground- and Excited-State Characterization

The ground- and excited-state properties of the series of photocatalysts were first measured under similar conditions and are tabulated in Table 1. Steady-state UV–visible absorption spectroscopy of the inorganic PCs was in line with expectations, i.e. Ru­(II) PCs exhibited the typical metal-to-ligand charge transfer (MLCT) band around 450 nm, with molar absorption coefficients (ε) around 15000 M^–1^cm^–1^ whereas the Ir­(III) PCs exhibited blue-shifted absorption spectra with smaller molar absorption coefficients in the visible spectral range. Tris-cyclometalated Ir­(III) PCs, such as [Ir­(CF_3_pmb)_3_] (**PC-14**) or [Ir­(dFppy)_3_] (**PC-9**) barely absorbed visible light.^49,50^ Acridinium (**PC-1–3**) and triphenylpyrylium (TPP) (**PC-12**) PCs absorbed efficiently between 350 and 480 nm with molar absorption coefficients that were in the 6000 M^–1^cm^–1^ and 27000 M^–1^cm^–1^ range, respectively. Benzophenone (**PC-13**) and acetophenone (**PC-15**) did not absorb visible light as their excited-state transitions are mostly located in the UV region. Almost all PCs exhibited room temperature photoluminescence from a triplet excited-state, with the exception of acridinium (**PC-1–3**) and triphenylpyrylium (**PC-12**) PCs that showed a singlet emissive excited state and a longer-lived nonradiative triplet state. The triplet energy of the different PCs was determined through photoluminescence recorded in frozen matrix using butyronitrile at 77 K, with the addition of iodomethane to enhance the intersystem crossing for the acridinium PCs. Importantly, these measurements highlighted that a wide range of triplet energies is covered within the selected series of PCs, with the lowest recorded triplet energy at 1.93 eV for Acr-Me (**PC-1**) and the largest at 3.21 eV for acetophenone (**PC-15**). In addition, the excited-state lifetime under Argon of the PCs was also determined, either by time-correlated single photon counting (TCSPC) or by nanosecond transient absorption spectroscopy for nonemissive compounds (Table 1). The excited-state lifetime ranged from 8.8 ns to 22.3 μs, which are all competent for bimolecular quenching processes. Finally, the ground state oxidation (*E*_ox_ = $E_{1/2}\left({PC}^{\left(n+1/n\right)}\right)$ ) and reduction (*E*_red_ = $E_{1/2}\left({PC}^{\left(n/n-1\right)}\right)$ ) potentials were determined by electrochemical measurements or taken from literature. Equations [Disp-formula Eq1] and [Disp-formula Eq2], where E_00_ is the energy stored in the excited state, were then used to derive the corresponding excited-state oxidation (*E*_ox_*) and reduction (E_red_*) potentials. Note that this equation is valid for single-electron transfer only.

$$E_{red}^{*}=E_{1/2}\left({PC}^{*n}/{PC}^{n-1}\right)=E_{1/2}\left({PC}^{\left(n/n-1\right)}\right)+E_{00}$$ 1

$$E_{ox}^{*}=E_{1/2}\left({PC}^{*n+1}/{PC}^{n}\right)=E_{1/2}\left({PC}^{\left(n+1/n\right)}\right)-E_{00}$$ 2

**Table 1:** Ground- and Excited-State Properties of the 15 Photocatalysts in Acetonitrile Used in This Study

Photocatalyst^a^	λ_abs,max_ (ε)/nm (10^3^ M^–1^cm^–1^)	λ_PL_/nm^a^	τ/ns^a^ ^,^ ^b^	E_red_ ^c^	E_ox_ ^c^	E_00_ ^d^	E*_red_ ^e^	E*_ox_ ^e^
Acr-Me (**PC-1**)	450 (5.0)	510; 540	11.6^S^; 22350^T^	–0.54 ^51^	---	2.66^S^; 1.93^T^	2.12^S^; 1.39^T^	---
Acr-Ph (**PC-2**)	450 (5.6)	500	11^S^; 4800^T^	–0.48 ^52^	---	2.63^S^; 1.96^T^	2.15^S^; 1.48^T^	---
Acr-^t^Bu (**PC-3**)	420 (6.8)	495; 515	17.3^S^; 6470^T^	–0.59 ^51^	---	2.69^S^; 2.03^T^	2.1^S^; 1.44^T^	---
[Ru(bpy)_3_]^2+^ (**PC-4**)	451 (14.4)	620	950	–1.30 ^48^	1.31 ^48^	2.16	1.18	–1.17
[Ru(TAP)_3_]^2+^ (**PC-5**)	436 (16.4)	597	50	–0.72 ^48^	>2.1 ^48^	2.23	1.51	>−0.13
[Ir(dFCF_3_ppy)_2_((CF_3_)_2_bpy)]^+^ (**PC-6**)	376 (4.7)	600	400	–0.74 ^53^	1.98 ^53^	2.44	1.70	–0.46
[Ir(Fppy)_2_(bpy)]^+^ (**PC-7**)	365 (9.6)	560	950	–1.32	1.46	2.53	1.21	–1.07
[Ir(ppy)_3_] (**PC-8**)	375 (5.3)	525	1550	–2.18 ^54^	0.82 ^54^	2.65	0.47	–1.83
[Ir(dFppy)_3_] (**PC-9**)	340 (5.1)	470; 490	1600	–2.10 ^55^	1.11 ^55^	2.76	0.66	–1.65
[Ir(dFCF_3_ppy)_2_(dtb)]^+^ (**PC-10**)	378 (5.5)	475; 495	2250	–1.35 ^56^	1.74 ^56^	2.76	1.41	–1.02
[Ir(dFppy)_2_(bpy)]^+^ (**PC-11**)	360 (7.0)	530	1270	–0.89 ^57^	2.09 ^57^	2.77	1.88	–0.68
2,4,6-Triphenylpyrylium (**PC-12**)	405 (37.3)	465	8.8^S^; 10000^T,^ ^58^	–0.31 ^59^	2.34 ^59^	2.83^S^; 2.3^T^	2.52^T^; 1.99^S^	–0.49^T^; 0.04^S^
Benzophenone (**PC-13**)	320 (0.05)	417	16800	–1.79 ^60^	---	2.99 ^49^	1.20	---
[Ir(CF_3_pmb)_3_] (**PC-14**)	295 (38.8)	415	6000	---	1.21	3.18	---	–1.97
Acetophenone (**PC-15**)	335 (0.13)	420	2800	–2.05 ^61^	---	3.21 ^49^	1.16	---

a Measured in acetonitrile under Argon at room temperature. S = Singlet and T = Triplet excited states

b Lifetime of the triplet excited state, unless otherwise specified.

c V vs Ag|AgCl.

d eV, determined from emission measurements at 77 K.

e V vs Ag|AgCl, calculated using eqs [Disp-formula Eq1] and [Disp-formula Eq2].

The *E*_ox_* was shown to span from – 0.13 to – 2.10 V vs Ag|AgCl, whereas corresponding E_red_* ranged from +0.47 to +2.52 V vs Ag|AgCl. Taken altogether, this ground- and excited-state characterization showcases a wide range of excited-state redox potentials and triplet energies which will enable the use of a Rehm–Weller analysis based on Stern–Volmer experiments to distinguish energy and electron transfer processes.

### Excited-State Quenching Experiments

With a clear description of the ground- and excited-state properties of the full series of PCs, we then investigated the excited-state reactivity between the PCs and two model 1,2-dialkyldiazene quenchers (diazenes **1** and **2**). In most cases, the excited-state quenching was evaluated by a combination of steady-state and time-resolved photoluminescence quenching experiments. A time-correlated single photon counting apparatus was used for all PCs, with the exception of acridiniums (**PC-1–3**), TPP (**PC-12**), [Ir­(CF_3_pmb)_3_] (**PC-14**), benzophenone (**PC-13**) and acetophenone (**PC-15**) that did not absorb at 447 or 507 nm, i.e. the two laser diodes available on our TCSPC apparatus. For these PCs, the excited-state quenching was determined using 355 nm laser irradiation on our transient absorption setup, using only the kinetic emission mode to investigate the quenching of the singlet emissive state for the organic PCs and the triplet emissive state for the iridium-based PCs. Finally, acridinium PCs (**PC-1–3**) exhibited a nonemissive triplet state in acetonitrile, and as such the Stern–Volmer experiments were performed using nanosecond transient absorption spectroscopy, monitoring the absorption changes over time of the dark excited state produced following pulsed-light excitation and intersystem crossing. The dark triplet state of TPP was not studied. A representative excited-state quenching experiment is shown for [Ir­(dFCF_3_ppy)_2_(dtb)]^+^ (**PC-10**) with diazene **1** (Figure 3A) while the other quenching experiments are gathered in the Supporting Information. The quenching rate constants (*k*_q_) are all gathered in Table 2.

**Table 2:** Quenching Rate Constants Recorded for Two Model Diazenes in Argon Purged Acetonitrile at Room Temperature

Photocatalyst	*k*_q_ (× 10^8^ M^–1^ s^–1^), Diazene **1**	*k*_q_ (× 10^8^ M^–1^ s^–1^), Diazene **2**
Acr-Me (**PC-1** ^**T**^)	0.10 ± 0.05	0.10 ± 0.05
Acr-Ph (**PC-2** ^**T**^)	0.15 ± 0.02	0.24 ± 0.03
Acr-^t^Bu (**PC-3** ^**T**^)	0.13 ± 0.04	0.11 ± 0.02
Acr-Me (**PC-1** ^**S**^)	25 ± 1	30 ± 1
Acr-Ph (**PC-2** ^**S**^)	28.1 ± 0.6	33.1 ± 0.9
Acr-^t^Bu (**PC-3** ^**S**^)	33.4 ± 0.5	38.9 ± 0.7
[Ru(bpy)_3_]^2+^ (**PC-4**)	0.2 ± 0.1	0.3 ± 0.1
[Ru(TAP)_3_]^2+^ (**PC-5**)	2 ± 1	3 ± 1
[Ir(dFCF_3_ppy)_2_((CF_3_)_2_bpy)]^+^ (**PC-6**)	0.38 ± 0.06	0.58 ± 0.05
[Ir(Fppy)_2_(bpy)]^+^ (**PC-7**)	1.6 ± 0.1	2.5 ± 0.1
[Ir(ppy)_3_] (**PC-8**)	6.5 ± 0.2	7.3 ± 0.1
[Ir(dFppy)_3_] (**PC-9**)	21.8 ± 0.6	22.6 ± 0.8
[Ir(dFCF_3_ppy)_2_(dtb)]^+^ (**PC-10**)	14.5 ± 0.6	12.3 ± 0.7
[Ir(dFppy)_2_(bpy)]^+^ (**PC-11**)	3.3 ± 0.1	6.1 ± 0.1
2,4,6-Triphenylpyrylium (**PC-12**)	35.9 ± 0.1	36.5 ± 0.3
Benzophenone (**PC-13**)	35.1 ± 0.1	30.8 ± 0.1
[Ir(CF_3_pmb)_3_] (**PC-14**)	20.1 ± 0.9	16.9 ± 0.5
Acetophenone (**PC-15**)	53 ± 2	63 ± 3

**Figure 3:**
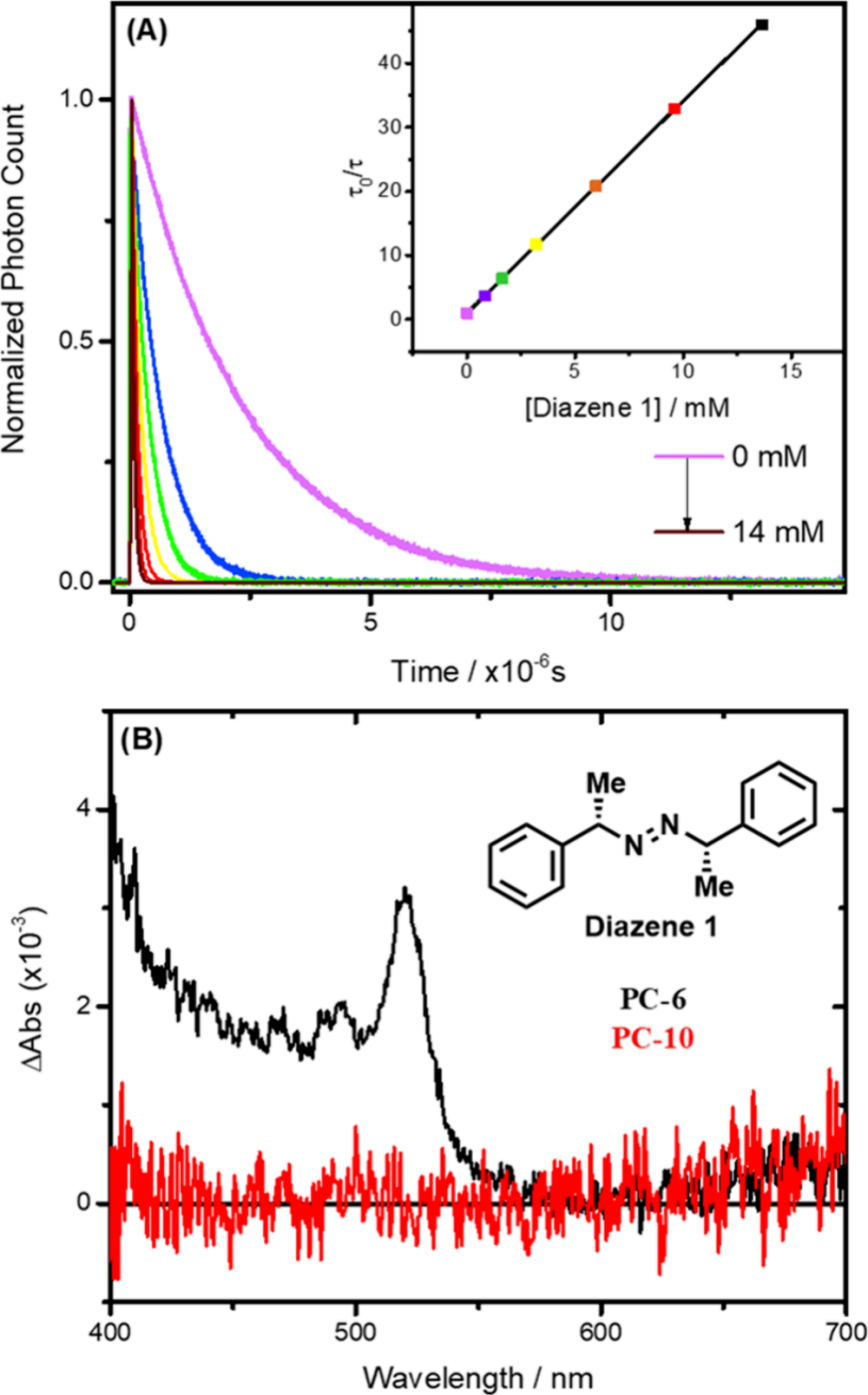
(A) Time-resolved photoluminescence quenching of [Ir­(dFCF_3_ppy)_2_(dtb)]^+^ in argon-purged acetonitrile with increasing concentrations of diazene **1**. The inset represents the corresponding Stern–Volmer plot from which the quenching rate constant, *k*_q_ = 1.4 × 10^9^ M^–1^s^–1^ was determined. (B) Nanosecond transient absorption spectroscopy of [Ir­(dFCF_3_ppy)_2_(dtb)]^+^ (**PC-10**) (red) and [Ir­(dFCF_3_ppy)_2_((CF_3_)_2_bpy)]^+^ (**PC-6**) (black) in the presence of diazene **1** (15 mM), recorded after the excited-state decay following pulsed 420 nm light excitation in argon purged acetonitrile. The presence of monoreduced **PC-6** was confirmed by the absorption changes presenting a maximum at 520 nm, in line with reference spectra generated using triethylamine as an electron donor (see Figure S54).

As emphasized in the introduction, the Stern–Volmer analysis allows to gain kinetic information about an excited-state quenching process but does not allow to determine through which mechanism the quenching is operating. Often, reaction mechanisms are inferred from Stern–Volmer experiments and related thermodynamic considerations, but only the detection of photoproducts, for example by UV–visible transient absorption spectroscopy, allows to confirm the nature of an excited-state quenching process with confidence. Nanosecond transient absorption spectroscopy of [Ir­(dFCF_3_ppy)_2_(dtb)]^+^ (**PC-10**) in the presence of diazene did not lead to the formation of any detectable photoproduct (Figure 3B, **red**). The absence of a signal precludes any definitive mechanistic conclusions, yet it allows to posit two main hypotheses. First, the excited-state quenching may operate via energy transfer, generating the triplet excited state of diazene **1**. Neither this species nor the fragmentation-derived radicals are expected to be detected, as their molar absorption coefficients are likely too small within the spectral range accessible to our nanosecond transient absorption setup. Alternatively, the reaction might proceed *via* excited-state electron transfer, in which case the reduced Ir photocatalyst should be observed around 520 nm. In this case, the absence of a signal would mean that the cage escape yield is very small and/or the molar absorption coefficient of the photoproducts is very low—both of which would be unprecedented for Ir­(III) photosensitizers.^62^ Nevertheless, to determine whether electron transfer products could be detected, we used [Ir­(dFCF_3_ppy)_2_((CF_3_)_2_bpy)]^+^ (**PC-6**) that exhibits a more positive E_red_* and smaller E_T_ than [Ir­(dFCF_3_ppy)_2_(dtb)]^+^ (**PC-10**). With this specific Ir complex, the nanosecond transient absorption spectroscopy was in line with excited-state electron transfer from the diazene to the excited PC, as indicated by the typical absorption changes of the reduced Ir PC at 520 nm (Figure 3B, **black**). The weak signal intensity originates from the moderate quenching rate constant (*k*_q_ = 3.8 × 10^7^ M^–1^s^–1^) and the small driving force resulting from an oxidation potential of 1.51 V for diazene **1** (Figure S20). Similar electron transfer products were also observed with the acridinium derivatives that represent potent photo-oxidant with moderate E_T_. In previous studies on model reversible electron donors, we have shown that [Ir­(dFCF_3_ppy)_2_(dtb)]^+^ (**PC-10**) leads to large cage escape yields upon bimolecular electron transfer.^63^ We expect that [Ir­(dFCF_3_ppy)_2_((CF_3_)_2_bpy)]^+^ (**PC-6**) behaves similarly and as such, since no photoproducts were observed with **PC-10**, we propose that quenching occurs via an excited-state energy transfer rather than electron transfer. Further experimental evidence corroborating this energy transfer mechanism will be presented in the following sections.

### Rehm–Weller Analysis

The determination of the quenching rate constant for all PCs with 1,2-dialkyldiazenes **1** and **2** ideally positioned us to use a Rehm–Weller-type analysis.^64^ The interplay of thermodynamic and kinetic factors, i.e. the free-energy change of electron transfer (ΔG_et_) and the associated rate constants, plays a decisive role in understanding excited-state quenching. When ΔG_et_ assumes moderately negative values, the rate of electron transfer tends to rise as the driving force becomes more negative. At sufficiently large driving forces, however, the experimentally accessible rate constant is no longer determined by the intrinsic energetics of the system but instead reaches the diffusion-controlled limit of the medium (*k*_diff_). An influential framework for describing this behavior was provided by Rehm and Weller, who established an empirical relationship between ΔG_et_ and the electron transfer rate constant (*k*_et_) or quenching rate constant (*k*_q_).^64^ An adaptation of this relationship was introduced by Balzani and co-workers for energy transfer photochemistry (eqs [Disp-formula Eq3] and [Disp-formula Eq4]).^65^ A brief overview of the evolution of the so-called Rehm–Weller-type Equations is discussed in the Supporting Information (Section **VII**).

$$k_{\mathrm{EnT}}=\frac{k_{\mathrm{diff}}}{1+\exp\left(\frac{\Delta G_{\mathrm{EnT}}}{k_{b}T}\right)+\frac{k_{-\mathrm{diff}}}{Z}\exp\left(\frac{\Delta G_{\mathrm{EnT}}^{\#}}{k_{b}T}\right)}$$ 3

$$\Delta G_{\mathrm{EnT}}^{\#}=\frac{\Delta G_{EnT}}{2}+{\left({\left(\frac{\Delta G_{EnT}}{2}\right)}^{2}+{\left(\frac{\lambda }{4}\right)}^{2}\right)}^{1/2}$$ 4

$$\mathrm{In}\;\mathrm{acetonitrile}\;\mathrm{at}\;\mathrm{room}\;\mathrm{temperature}:\;k_{EnT}=\frac{2\times 10^{10}\;M^{-1}\;s^{-1}}{1+\exp\left(\frac{\Delta G_{EnT}}{0.0252\;\mathrm{eV}}\right)+0.25\left[\exp\left(\frac{\Delta G_{EnT}^{\#}}{0.0252\;\mathrm{eV}}\right)\right]}$$ 5

In these equations, *k*_EnT_, ΔG_EnT_ and ΔG^#^_EnT_ are the rate of energy transfer, the free-energy change of energy transfer and the activation free energy for energy transfer, respectively. For acetonitrile, the diffusion-controlled limit of 2 × 10^10^ M^–1^s^–1^ sets the upper boundary for observable *k*_et_ values (eq [Disp-formula Eq5]).^66^ The pre-exponential factor of 0.25, which corresponds to *k*_–diff_/Z, was extracted by Rehm and Weller based on the solvent’s collision frequency (Z) and the dissociation rate constant. The thermal energy *k*_b_T is estimated as 0.0252 eV.

Herein, the quenching rate constants obtained for the different photocatalyst/diazene combinations were investigated with respect to the triplet energy of the photosensitizers (*k*_q_ vs E_T_, Figures 4A and 4B) or versus their excited-state redox potentials (*k*_q_ vs E_red_* and *k*_q_ vs *E*_ox_*, Figures S55–S58). For sake of clarity, the symbols are filled when photoproducts corresponding to excited-state electron transfer were observed by nanosecond transient absorption spectroscopy and empty when no photoproducts were detected. The PCs with singlet excited states, such as singlet acridinium (**PC-1–3** ^**S**^) and TPP (**PC-12**) are not included in the plot due to the spin-forbidden energy transfer according to the spin multiplicity conservation rule. In addition, PCs exhibiting detectable electron-transfer products were also excluded (**PC-1–3** ^**T**^), except for **PC-6**, which exhibited a combination of both energy- and electron-transfer processes, yet was previously shown to be competent for deaminative arylation using symmetrical 1,2-dialkyldiazenes.^24^ As shown in Figures 4A and 4B, *k*_q_ correlates remarkably well with the triplet energy of the screened photocatalysts. Quenching rate constant increases as the triplet energy of the PC increases and level off near 10^10^ M^–1^s^–1^, consistent with the diffusion limit in acetonitrile.^66^ By fixing the reorganization energy (λ) to 1 eV in eq [Disp-formula Eq4], the best fits were obtained using eq [Disp-formula Eq5], yielding the triplet energy of the two model diazenes estimated as 2.3 eV. A value of 1 eV for the reorganization energy, often used in the literature, provided the best fit. Modifying this value between 0.5 and 1.5 eV led to large discrepancy between the data and the fit (see Figure S68). In contrast, when the quenching rate constants are plotted as a function of the excited-state reduction potentials, a trend is not apparent and the data points are scattered around the plot (Figures S55–S58). Therefore, this analysis not only provides strong evidence that the excited-state quenching is occurring via photoinduced energy transfer but also allows to determine the triplet energy of the quencher that can be used for further optimization of the substrate scope or reaction conditions (*vide infra*).

**Figure 4:**
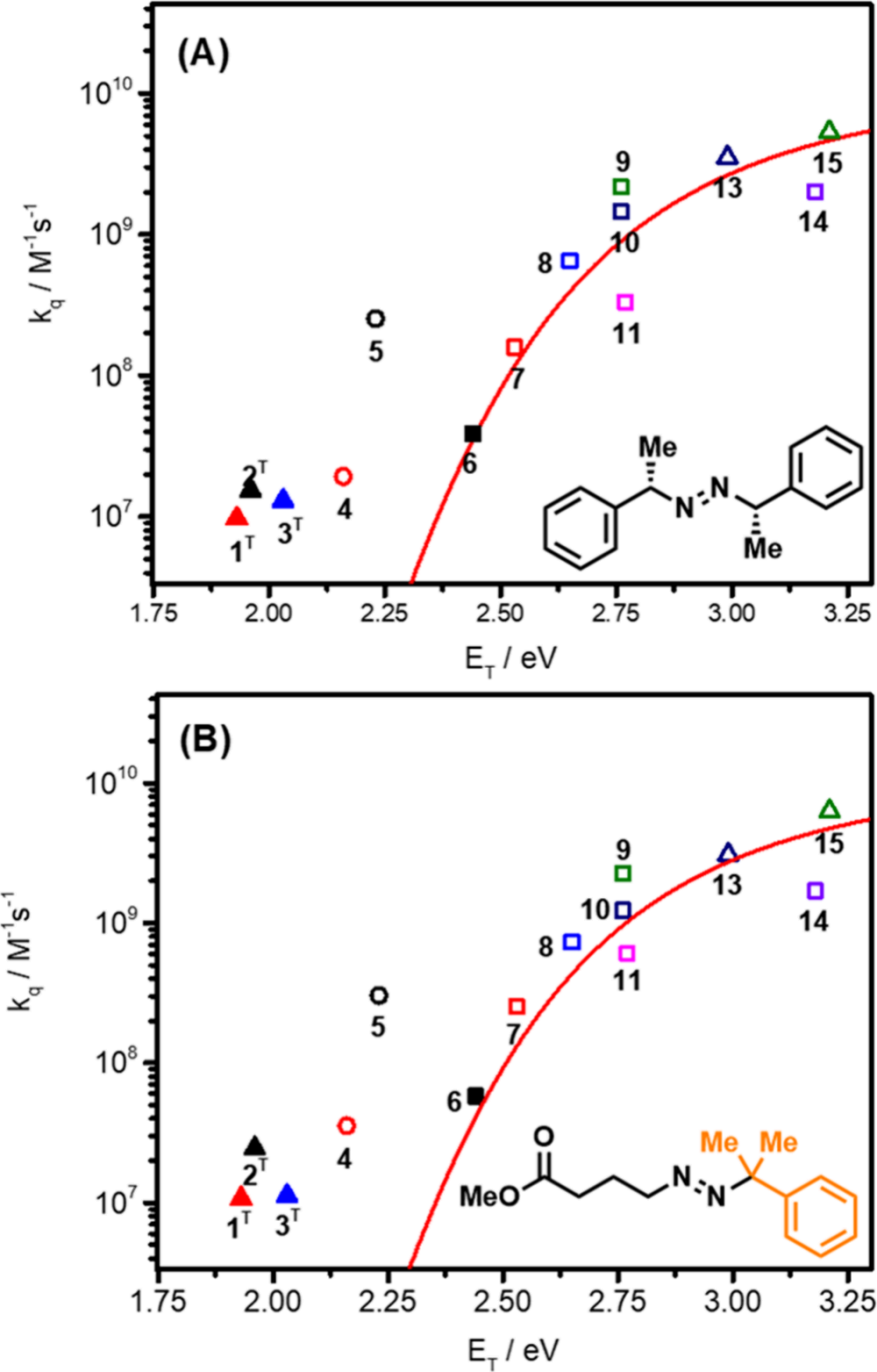
Rehm–Weller plot gathering the 15 PCs as a function of energy stored in their triplet excited state. Experiments were carried out in argon purged acetonitrile at room temperature using diazenes **1** and **2**. Ir­(III) PCs are represented by a square, Ru­(II) PCs by a circle and organic PCs by a triangle. Symbols are filled when photoproducts corresponding to excited-state electron transfer were observed and empty when no photoproducts were detected. Numbers refer to PCs in Table 1. The PCs with singlet excited state are not included in the plot due to a spin-forbidden energy transfer.

### Mediator-Enhanced Triplet Energy Transfer Strategy

Finally, with knowledge of the triplet energies of the diazenes, we sought to develop a mediator-enhanced triplet energy transfer pathway (Figure 5) to allow further indirect evidence for the occurrence of a spectroscopically invisible intermediate. In this approach, the energy is first transferred from the excited-state iridium PC to diazene **1**, and subsequently from triplet diazene ^3^ **1** to anthracene (An), which provides well-defined UV–visible spectroscopic signatures. The absorption spectrum of triplet anthracene (^3^An) was first established via direct triplet–triplet energy transfer, using [Ru­(bpy)_3_]^2+^ (**PC-4**) as the photosensitizer. Distinct new UV–visible absorption bands at 405 and 425 nm were observed in acetonitrile. We next investigated the excited-state quenching of [Ir­(dFCF_3_ppy)_2_(dtb)]^+^ (**PC-10**) by An and determined a quenching rate constant of 7.7 × 10^9^ M^–1^s^–1^. This value falls within the same range as that obtained for the diazene (*k*_q_ = 1.4 × 10^9^ M^–1^s^–1^). Considering both the excited-state lifetime of **PC-10** and the measured quenching rate constants, we performed quenching experiments in the presence of 0.1 mM An and 2.8–7.8 mM diazene **1**. Under these conditions, **PC-10** is expected to be quenched by both substrates. However, considering the contribution of both quenchers in relation to the total quenching, the maximal absorption changes for ^3^An generated solely by direct triplet–triplet energy transfer between **PC-10** and An can be calculated using eq [Disp-formula Eq6] (See Supporting Information, Section **VIII** for details). Thus, using a diazene concentration of 7.8 mM, the theoretical maximum was found to be 25.2 mOD at 425 nm.

ΔAMax−Anth@425=ΔεAnth@425×Cmax−AnTth=ΔεAnth@425×ϕAnth×[Ir*] 6

where Δ*A*_Max–Anth@425_ is the maximum absorption difference of ^3^An at 425 nm, Δ*ε*_Anth@425_ is the difference in molar absorption coefficient of ^3^An at 425 nm, *C*_max–^T^Anth_ is the maximum concentration of ^3^An formed directly by the quenching of **PC-10**, ϕ_Anth_ is the fractional contribution of An in the quenching of PC* (often referred to as the quenching efficiency) and [Ir*] is the concentration of excited iridium PC determined by actinometry.

**Figure 5:**
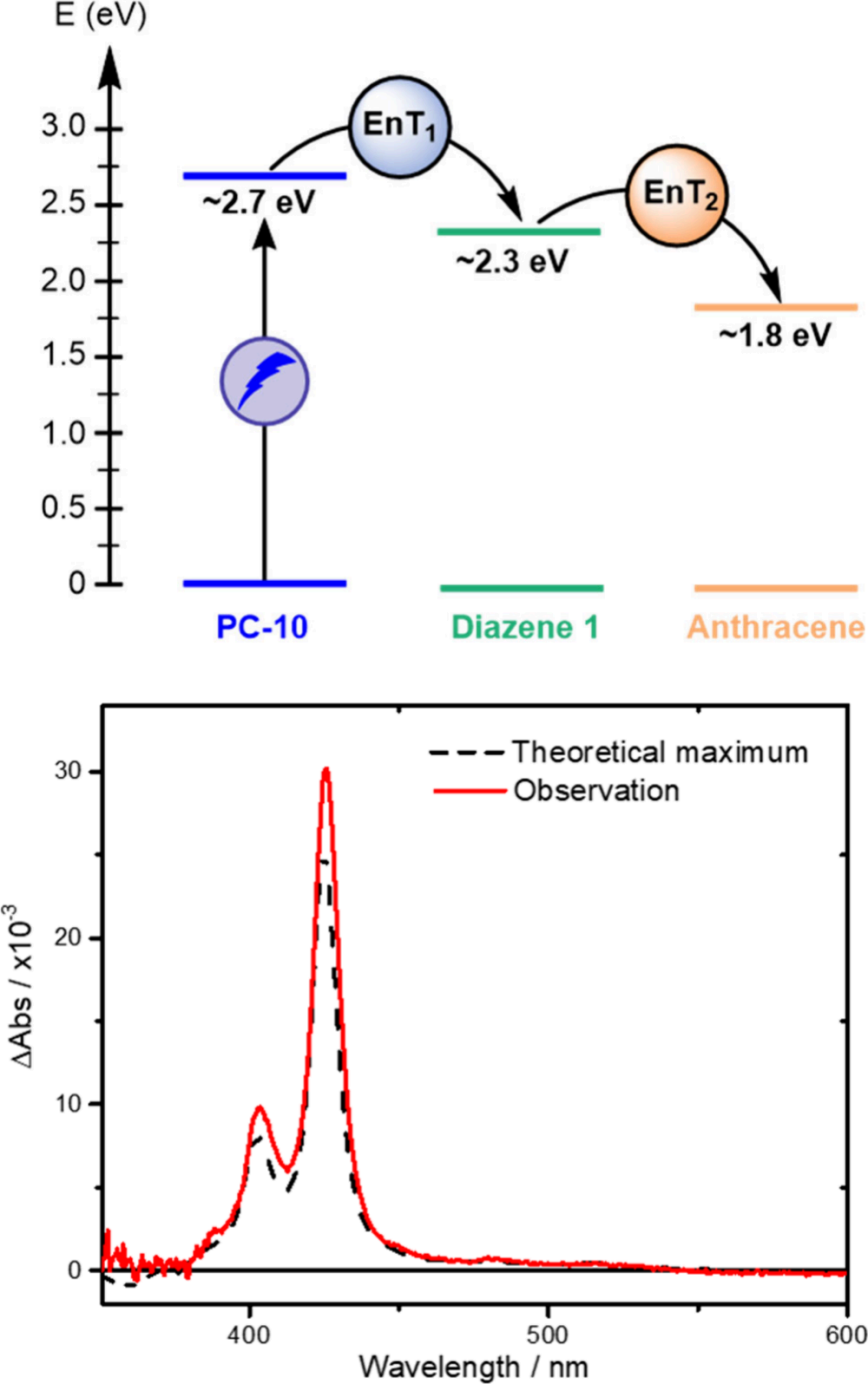
Top: Mediator-enhanced triplet energy transfer strategy where the energy is first transferred to the unobservable diazene and then to the anthracene with prototypical visible absorption signatures. Bottom: Nanosecond transient absorption spectra obtained following pulsed-light excitation of a solution containing **PC-10** (15 μM), diazene **1** (7.8 mM) and anthracene (0.1 mM) (solid) compared to the theoretically maximum (dashed) amount of ^3^An that could be formed by direct triplet–triplet energy transfer (EnT) from the iridium PC.

Gratifyingly, nanosecond transient absorption spectroscopy reproducibly yielded values exceeding this theoretical limit by 20% on average, thereby providing clear evidence for the mediator-enhanced triplet energy transfer process and confirming that initial energy transfer from **PC-10** to diazene **1** occurs.

### Influence of Triplet Sensitization over the Fate of the Geminate Radical Pair

Having established that diazene activation proceeds via energy transfer, we next sought to investigate the product distribution following homolytic bond cleavage. The generation of triplet radical pairs through photosensitization was anticipated to enhance cage escape of the geminate radical pair relative to direct UV irradiation, thereby enabling nickel capture and productive C­(sp^3^)–C­(sp^2^) cross-coupling. While singlet radical pairs recombine readily, triplet pairs require comparatively slow intersystem crossing (≈10^–8^ s), a process that outlasts the solvent cage lifetime (≈10^–10^ s) and consequently favors cage escape.^67–70^ Such spin-state control over reactivity profile has been examined in constrained systems at low temperature but remains unexplored for linear 1,2-dialkyldiazenes under ambient conditions.^29,31^

Taking inspiration from the seminal radical crossover experiments by Lyon and Levy using protio- and deutero-azomethane,^71^ we set out to monitor the fate of the alkyl radical generated under blue-light excitation using high triplet-energy photocatalyst **PC-10** (1 mol%) *via* ^1^H NMR spectroscopy. Diazene **3** was chosen because the stability of the substituted benzylic radicals facilitates fragmentation under UV irradiation, allowing a direct comparison of product distributions between blue and UV light excitation (450 vs 350 nm). In line with our prior report,^25^ blue light irradiation with PC-**10** afforded all three possible radical recombination products: cross-recombination product **4**, formed both within (**4**_*in*_) and outside (**4**_*out*_) the solvent cage, as well as out-of-cage products **5** and **6**, with a total mass recovery around 90%. These results indicate that radical recombination is the dominating reaction pathway.^32^ Of note, **6** was formed as a mixture of diastereomers (d.r. = 1:1, see Supporting Information). The observed ratio of products **4**:**5**:**6** was 2.4:1.1:1.0 (Figure 6A,B), which is close to the statistical distribution expected for a diffusion-limited out-of-cage process. Because cage-escape products **5** and **6** were formed in nearly equal amounts, the 2° and 3° benzylic radicals were inferred to have similar reactivity. Therefore, we hypothesized that the amount of cross-product **4**_*out*_ formed outside of the solvent cage should be approximately equal to the combined amount of **5** and **6**. Consistent with the observed product distribution, application of eq [Disp-formula Eq7] affords an estimated cage-escape yield (ϕ_CE_) of approximately 95% under the blue-light energy-transfer manifold, which is considerably higher than the previously estimated cage effect on symmetrical 1,2-dialkyldiazene fragmentation under thermal conditions.^72^

$$\phi_{\mathrm{CE}}=\frac{\left[Pdt_{\mathrm{out}}\right]}{\left[Pdt_{\mathrm{in}}\right]+\left[Pdt_{\mathrm{out}}\right]}=\frac{\left[4_{\mathrm{out}}\right]+\left[5\right]+\left[6\right]}{\left[4_{\mathrm{in}}\right]+\left[4_{\mathrm{out}}\right]+\left[5\right]+\left[6\right]}$$ 7

**Figure 6:**
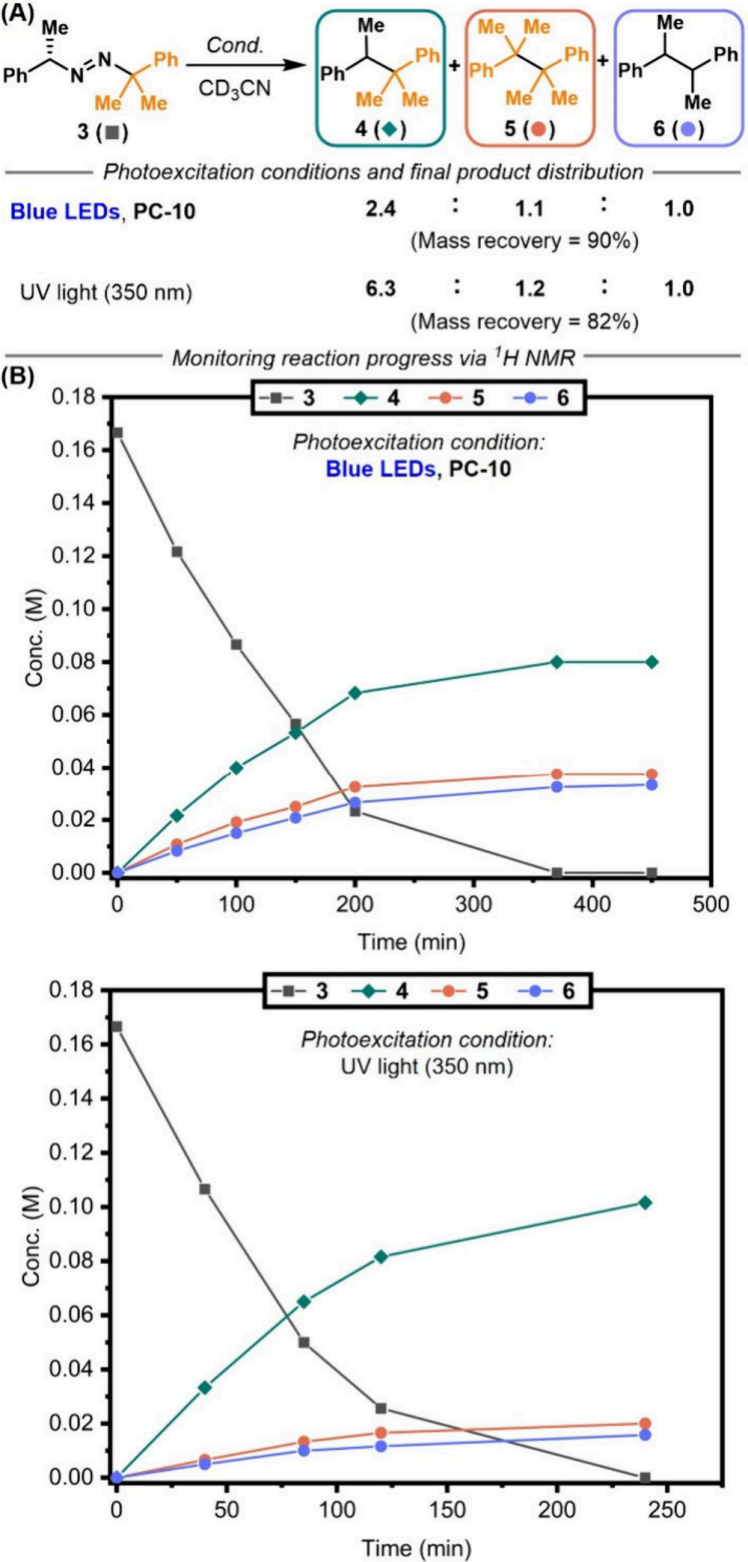
(A) Final product distribution resulting from the photodegradation of diazene **3** under distinct photoexcitation conditions as determined *via* ^1^H NMR spectroscopy. (B) Monitoring the kinetics of photodegradation of **3** using **PC-10** under blue LED irradiation demonstrates enhanced cage escape yield when compared to the photodegradation under UV light (350 nm) that favors in-cage recombination toward **4**. Unfitted lines were added to help the reader distinguish between the data sets.

In contrast, irradiation of diazene **3** at 350 nm (UV light) produced cross-product **4** in nearly 6-fold higher proportion relative to **5** or **6** (Figure 6A,B). Under the same assumptions and positing the absence of in-cage spin crossover, the cage-escape yield (ϕ_CE_) under UV light excitation was estimated at approximately 60%. With solvent, concentration, and temperature kept constant, this enhanced formation of **4** under UV excitation is attributed to faster in-cage recombination and therefore a lower cage-escape yield. Taken together, these findings corroborate the intermediacy of a triplet radical pair under blue-light energy-transfer conditions, enhancing cage escape via spin-forbidden recombination^62,63,73^ and thereby enabling productive cross-coupling with aryl bromides.

### Kinetics of Photofragmentation for Various Diazenes

Despite their structural differences, model 1,2-dialkyldiazenes **1** and **2** exhibited nearly identical quenching rate constants with **PC-10** in the Stern–Volmer experiments. Rehm–Weller analyses further confirmed that both substrates possess comparable triplet energies (E_T_) (Figure 4A,B), indicating that triplet–triplet energy transfer from **PC-10** should proceed with similar efficiency. Based on this hypothesis, we next examined how structural variations influence the relative photofragmentation rates. To minimize perturbations from photon flux and other external variables, these comparisons were performed as competition experiments rather than independent kinetic measurements. Under blue light photosensitization conditions, model diazene **2** was found to decay faster than symmetrical diazene **1** (Figure 7A). Assuming first-order decay with respect to diazene, rate constants were extracted for both substrates. Although the formation of a more stabilized cumyl radical may account for the enhanced fragmentation rate of diazene **2**, other factors beyond bond-dissociation energy (BDE) likely contribute, including the rate of *trans*–*cis* isomerization, a key step proposed in the photodegradation mechanism of 1,2-dialkyldiazenes.^74^ Consistent with the BDE trend, diazene **3** was found to fragment nearly twice as fast as diazene **1**, further supporting the efficiency of the cumyl substituent in promoting radical generation (Figure 7B).

**Figure 7:**
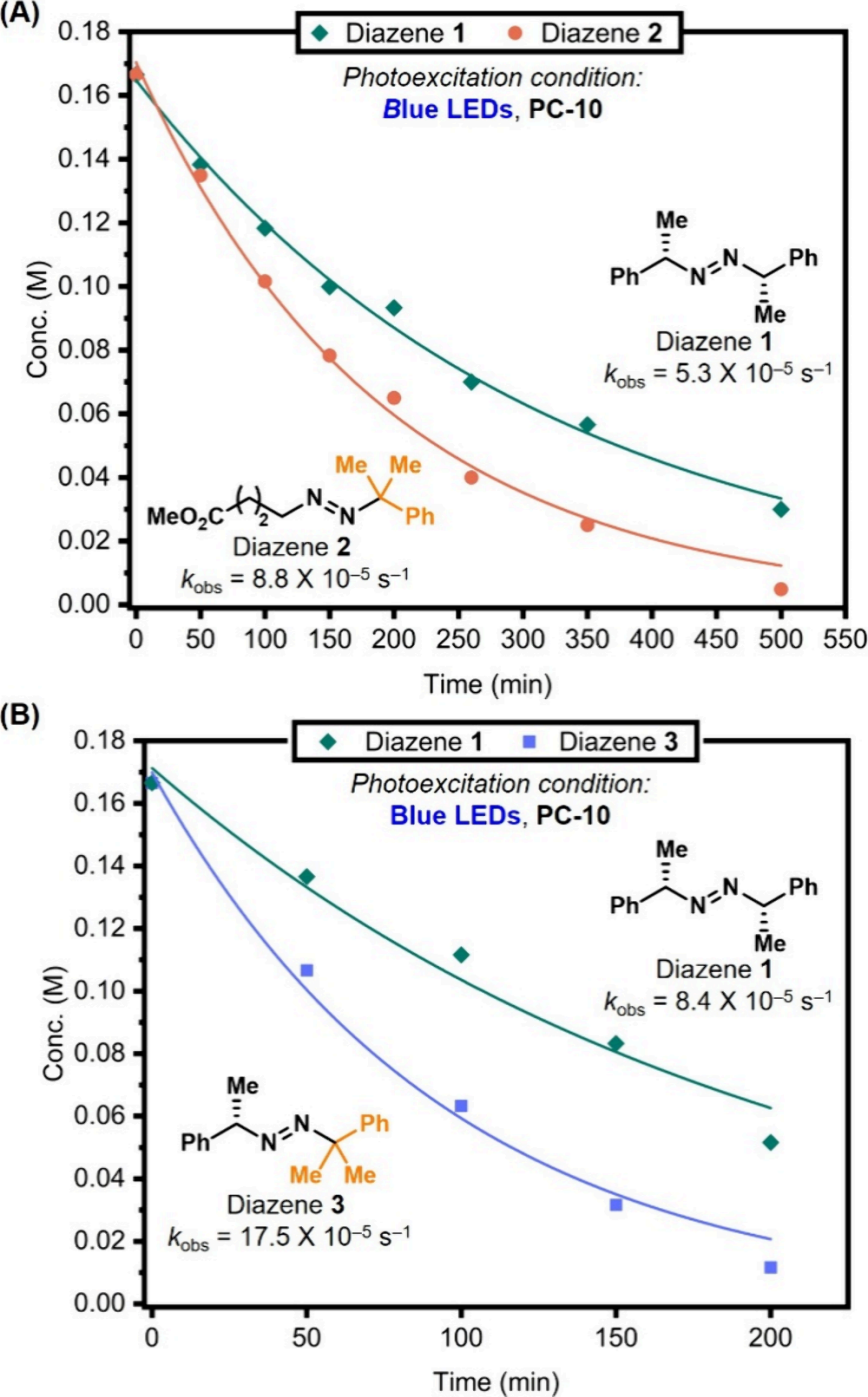
Competition experiments on the fragmentation of diazenes **1–3** under blue-light irradiation in the presence of **PC-10** (2 mol %) indicate that the photofragmentation rates are substrate-dependent: (A) Diazene **1** vs diazene **2**; (B) diazene **1** vs diazene **3**. Exponential fits following c­(t) = c_0_ × exp­(–*k*_obs_t) are provided for comparison.

### Harnessing Kinetic Matching to Improve Cross-Coupling Reaction Efficiency

A common limitation affecting the scope of deaminative arylations stems from the poor reactivity of electron-rich aryl bromides likely caused by sluggish oxidative addition at either Ni(0) or Ni­(I) species.^36,75,76^ Capitalizing on the independent nature of the dual catalytic cycles, we envisioned that a photosensitizer with a lower triplet state energy would generate the radical pairs at a reduced rate, thus enabling a *kinetic matching* with the slow oxidative addition of less reactive aryl bromides without significantly altering other reaction parameters. Although rate matching has been invoked as a key contributing factor toward cross-selectivity in Ni catalyzed radical couplings, it is typically achieved through complex ligand design or tailored activating groups.^77–79^ Applying this concept to photocatalyst selection would provide a general and practical design principle for modulating reactivity in radical cross-coupling.

The fragmentation kinetics of model diazene **2** were evaluated under blue light using **PC-6** and **PC-10**, two photosensitizers differing in triplet energies (2.44 and 2.76 eV, respectively) and quenching rate constants (5.8 × 10^7^ and 1.23 × 10^9^ M^–1^s^–1^, respectively) (Tables 1 and 2). Consistent with its lower *k*_q_, **PC-6** promoted diazene fragmentation at a lower *k*_obs_ compared to **PC-10** (Figure 8A). Notably, guided by this mechanistic insight, the selection of **PC-6** enabled the cross-coupled product **8a** to be obtained in 48% yield from electron-rich aryl bromide **7a** and diazene **2**, whereas **PC-10**, previously identified as optimal catalyst, led to a 2-fold lower yield under identical conditions (Figure 8B).^25^ The efficiency of the cross-coupling between **7a** and **2** using **PC-10** could be restored by lowering the light intensity (Figure 8B), consistent with improved synchronization between geminate radical-pair formation and oxidative addition. Employing both a lower light intensity and a less efficient photocatalyst (**PC-6**) resulted in incomplete fragmentation of diazene **2** (92% conversion) and, consequently, diminished formation of **8a**, highlighting the importance of achieving an optimal kinetic match. These observations support a kinetic-matching model in which controlled radical generation better aligns with challenging oxidative addition events, whereas faster rates of diazene fragmentation are advantageous for more reactive aryl bromides. In line with these findings, **PC-10** continued to outperform **PC-6** with electron-deficient aryl bromides that undergo faster oxidative addition (Figure S2).

**Figure 8:**
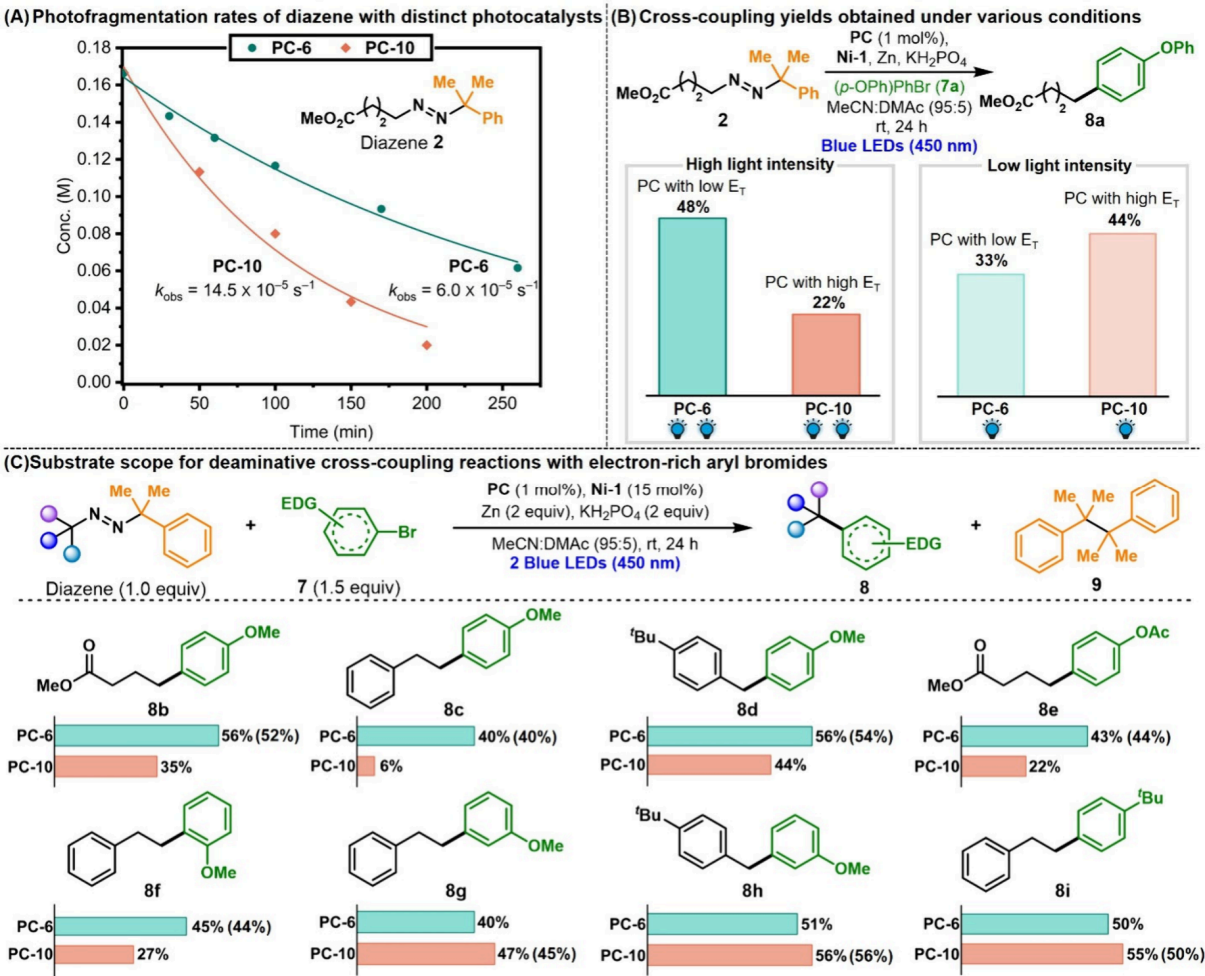
(A) Photofragmentation kinetics of diazene **2** in the presence of photocatalysts **PC-6** and **PC-10**. Exponential fits following c­(t) = c_0_ × exp­(–*k*_obs_t) are provided for comparison. (B) Investigating the influence of the Ir PC and light intensity on the coupling of **2** and **7a**. Yields were determined by ^1^H NMR using phenyltrimethylsilane as internal standard. **Ni-1** = NiBr_2_·(4,4′-Di-*tert*-butyl-2,2′-dipyridyl). (C) Yield optimization for electron-rich partners enabled by kinetic matching and measured using ^1^H NMR. Isolated yields were measured for the optimal conditions for each substrate and are indicated in parentheses. EDG = Electron donating group.

Our improved conditions for the cross coupling with electron-rich aryl bromides were tested across a range of substrates. *p*-bromoanisole (**7b**) showed consistently higher yields with **PC-6** relative to **PC-10**, regardless of the diazene partner (**8b–8d**) (Figure 8C). Impressively, **8c** was isolated in 40% yield with **PC-6** and only 6% yield with **PC-10**. Replacing the *p*-methoxy substituent with an acetate group did not change this trend (43% vs 22% for **8e**), with **PC-6** still proving more effective than **PC-10** despite the lower electron-donating ability of the substituent. A similar preference for **PC-6** was also observed with *o*-bromoanisole and the yield of resulting product **8f** (45% vs 27%). In contrast, *m*-OMe substituted aryl bromide showed no significant difference when used with either **PC-6** or **PC-10** (**8g**, **8h**), consistent with the minimal effect of a *meta*-donor on aryl π-electron density and oxidative addition. Similarly, a *p-tert*-butyl substituent did not distinguish between the photocatalysts, likely due to its weaker electronic activation and relatively rapid oxidative addition compared to alkoxy-substituted phenyl bromides (**8i**). Beyond expanding the scope of deaminative arylation, these results provide a conceptual framework for rational photocatalyst selection under energy-transfer control, affording a practical knowledge to improve reaction yield.

## Conclusions

A systematic investigation of 15 inorganic and organic photocatalysts was undertaken to elucidate the mechanism of excited-state quenching by diazene. This carefully curated set of photocatalysts spanned triplet energies from 1.93 to 3.21 eV and excited-state reduction potentials from +0.47 to +2.52 V vs Ag|AgCl, enabling a rigorous mechanistic analysis. A combination of Stern–Volmer quenching experiments and Rehm–Weller type analysis provided compelling evidence that the quenching pathway proceeded via triplet–triplet energy transfer. Importantly, this analysis also enabled the estimation of the triplet energy of the diazene substrates at 2.3 eV, thereby offering a quantitative benchmark for the rational design of next-generation photocatalysts tailored for such substrates in diverse synthetic platforms.

The energy transfer pathway was further validated through a mediator-enhanced triplet energy transfer strategy, wherein energy transfer first occurred from the iridium photocatalyst to the diazene and was subsequently relayed to an anthracene acceptor with distinct UV–visible spectroscopic features which has been observed and quantified by nanosecond transient absorption spectroscopy, providing strong evidence for the proposed mechanism. A direct comparison of distinct photoexcitation modes further revealed an enhanced cage-escape yield under triplet photosensitization, consistent with the generation of a triplet radical pair. Despite similar triplet energies, diazenes displayed distinct photofragmentation kinetics dictated by structural factors. Importantly, a slower radical-generating photocatalyst enabled significantly improved cross-coupling outcomes for challenging electron-rich aryl bromides. This could be achieved by using a photocatalyst with decreased quenching efficiency, or by modulating the irradiation intensity.

Building on these observations, these results invite a broader re-evaluation of how light-induced reactions are typically optimized and interpreted. Standard optimization workflows prioritize the identification of the most efficient photocatalyst, highest light intensity, and fastest quenching kinetics, followed by an assessment of substrate scope. Substrates that fail under these “optimal” conditions are commonly classified as intrinsic limitations of the transformation. However, such an approach may overlook mechanistically informative regimes in which slower radical generation, accessed through attenuated light flux or less performant photocatalysts, fundamentally alters reaction outcomes. Introducing a second, mechanistically guided optimization phase on less performant substrates, in which light intensity is deliberately modulated and photocatalysts with lower apparent efficiency are systematically re-examined, could uncover reactivity masked under high-flux conditions. In this context, reduced radical concentrations may suppress unproductive pathways and enable challenging bond-forming events, transforming apparent limitations into opportunities for deeper mechanistic insight and expanded reactivity.^59^

Given that energy transfer processes are often challenging to probe, owing to the weak spectroscopic signatures and low molar absorption coefficients of triplet excited states of organic molecules in the visible light range, the complementary approaches presented herein provide a powerful framework for future studies. Indeed, diazene derivatives have recently experienced a resurgence as key reaction intermediates in diverse cross-couplings.^80–83^ Overall, this work not only establishes a reliable experimental protocol for distinguishing energy transfer from alternative quenching pathways but also provides essential design principles to leverage the activation via energy transfer of a wide range of 1,2-dialkyldiazenes in the future design of radical cross-couplings.

## Supplementary Material



## Notes

Additional experimental details, Stern–Volmer experiments, photophysical characterization, transient absorption spectra (PDF)
