# Supplementary material for: Triplet Energy Transfer as a Handle to Tune 1,2-Dialkyldiazene Fragmentation in Radical C(sp3)–C(sp2) Cross-Coupling
Source: J Am Chem Soc. 2026 Feb 17;148(8):8993–9005. doi: 10.1021/jacs.5c22244 (PMC12964421; doi:10.1021/jacs.5c22244)
Supplement: Supplementary file 1 [file ja5c22244_si_001.pdf]

Supporting Information

Triplet Energy Transfer as a Handle to Tune 1,2-Dialkyldiazene Fragmentation in Radical C(sp^3)–C(sp^2) Cross-Coupling

Joffrey Scriven,[‡, #] Deepta Chattapadhyay,[†, #] Felix Glaser,[‡] Benjamin Elias,[‡] Quentin Michaudel,[†,Δ*] and Ludovic Troian-Gautier[‡,Φ*]

[‡] UCLouvain, Institut de la Matière Condensée et des Nanosciences (IMCN), Molecular Chemistry, Materials and Catalysis (MOST), Place Louis Pasteur 1/L4.01.02, B-1348 Louvain-la-Neuve, Belgium
[†]Department of Chemistry, Texas A&M University, College Station, Texas 77843, United States
[Δ]Department of Materials Science and Engineering, Texas A&M University, College Station, Texas 77843, USA
[Φ] Wel Research Institute, Avenue Pasteur 6, 1300 Wavre, Belgium

[#]Both authors equally contributed

Correspondence to: quentin.michaudel@chem.tamu.edu, Ludovic.Troian@uclouvain.be

Table of Content

I. General Information S2
II. Synthesis and Characterization........ S4
Synthesis of Symmetrical Diazene **1**: S4
Synthesis of Unsymmetrical Cumyl Diazenes: General procedure A S5
Synthesis and characterization of cumyldiazenes........ S6
Diazene fragmentation and cross-coupling reaction: General procedure B S7
Synthesis and characterization of cross-coupling products S8
III. Absorption and Emission Spectroscopies S11
IV. Electrochemistry........ S18
V. Stern-Volmer Analyses S20
Quenching measurement with Diazene **1** S20
Quenching measurement with diazene **2** S27
VI. Nanosecond Transient Absorption Spectroscopy........ S35
VII. Rehm-Weller Analyses........ S38
VIII. Mediator-enhanced triplet energy transfer strategy........ S42
IX. Reaction Progress Monitoring via ^{1}H NMR........ S46
Product distribution under photosensitized fragmentation of diazene **3**:........ S46
Product distribution under UV irradiation of diazene **3**: S47
Competitive photofragmentation of diazene pair: S48
Photofragmentation kinetics of diazene **2** with photocatalysts of different triplet energy: S50
X. Value of λ in the Rehm-Weller plot S53
XI. References S54
XII. NMR Spectroscopy S56

I. General Information

General Reagent Information. All reactions were performed without any precaution for moisture and oxygen unless otherwise stated. Dry acetonitrile (MeCN) was obtained by passing the previously degassed solvents through activated alumina columns. All starting materials and reagents were purchased at the highest commercial quality and used without further purification unless otherwise stated. All photocatalysts were commercially available unless otherwise noted. Anhydrous methanol was purchased from Sigma-Aldrich as acroseal bottle or was distilled (VWR) under argon before being used. $NiBr_2$•(dtbbpy) (**Ni-1**) was synthesized following reported procedure.[1] Zn powder was activated following reported procedure prior to use in the cross-coupling reactions.[2] The reactions were heated using an oil bath unless otherwise stated. Yields refer to chromatographically and spectroscopically (^{1}H NMR) homogeneous material, unless otherwise stated. Reactions were monitored by thin layer chromatography (TLC) carried out on 250 μm SiliCycle SilicaPlate™ silica plates (F254), using UV light as the visualizing agent and an acidic solution of phosphomolybdic acid (PMA) and heat or ninhydrin and heat as developing agents. Flash silica gel chromatography was performed using SiliCycle SilicaFlash® Irregular Silica Gel (60 Å, particle size 40–63 μm). Photochemical reactions were carried conducted using a Hepatochem EvoluChem™ PhotoRedOx Box Duo device and irradiated with two EvoluChem™ P303-30-1 LEDs (30 W, λ_{max} = 450 nm) with a cooling fan, or using Kessil PR160L lamp (30 W, 456 nm) with external cooling fan. All photochemical reactions were performed in borosilicate glass vials (or in J. Young NMR tubes for kinetic measurements).

General analytical information. Characteristic ^{1}H NMR spectra were recorded using JEOL JNM-ECZL-400R (400 MHz) or 600G (600 MHz) spectrometers at UCLouvain, or on two Bruker Avance NEO 400 MHz and a Bruker Avance 500 MHz at Texas A&M university. ^{13}C NMR spectra were recorded on a Bruker Avance 500 MHz and a Bruker Avance NEO 400 MHz; ^{19}F NMR spectra were recorded using a Bruker Avance NEO 400 MHz instrument. All ^{1}H and ^{13}C NMR spectra were calibrated using residual deuterated solvent as an internal reference ($CDCl_3$ @ 7.26 ppm ^{1}H NMR, 77.16 ppm ^{13}C NMR; DMSO-d_6 @ 2.50 ppm ^{1}H NMR, 39.52 ppm ^{13}C NMR). Coupling constants (J) are reported in Hertz (Hz). The following abbreviations were used to explain NMR peak multiplicities: s = singlet, d = doublet, t = triplet, q = quartet, p = pentet, m = multiplet, br = broad. High- resolution mass spectra (HRMS) were recorded on an Agilent LC/MSD TOF mass spectrometer by electrospray ionization time-of-flight (ESI-TOF) reflection or atmospheric-pressure chemical ionization (APCI) experiments.

UV-Visible Absorption. UV−vis absorption spectra were recorded on an Agilent Cary 60 spectrophotometer in a 1 cm path length quartz cuvette.

Time-resolved and steady-state photoluminescence. Time-resolved and steady-state photoluminescence spectra were recorded on an Edinburgh Instruments FS5 Spectrofluorometer equipped with a time-correlated single photon counting module. The steady-state photoluminescence spectra were recorded using a 150 W Xenon arc lamp as the excitation source. The photoluminescence was detected at a right angle to the excitation beam using a single photon counting PMT-900 in a temperature stabilized housing. The spectra were integrated at 0.1–0.2 s and three spectra were averaged. Steady-state photoluminescence spectra were corrected for the instrument's spectral response. All room-temperature spectra were obtained from Argon-sparged solutions unless otherwise mentioned.
Time-resolved photoluminescence data were collected on the FS5 using the time-correlated single photon counting technique (TCSPC) or on an LP980-K spectrometer from Edinburgh Instruments (see "transient absorption spectroscopy" for full description). Excitation was achieved with a 510 ± 5 nm diode laser (Edinburgh Instruments EPL-510, 90 ps pulse width at 10 MHz). Photons reaching the detector were accumulated to reach a count of 10000.

Transient absorption spectroscopy. Nanosecond transient absorption measurements were recorded on an LP980-K spectrometer from Edinburgh Instruments equipped with an iCCD detector from Andor (DH320T). The excitation source was a tunable Nd:YAG Laser NT342 Series from EKSPLA. The third harmonic (355 nm) at 150 mJ was directed into an optical parametric oscillator (OPO) to enable wavelength tuning starting from 410 nm. The laser power was then attenuated to reach appreciable signal/noise and the integrity of the samples was verified by UV-vis measurements. The LP980-K is equipped with a symmetrical Czerny-Turner monochromator. For single-wavelength absorption changes, a 1,800 g mm^{-1} grating, blazed at 500 nm is used, which affords wavelength coverage from 200 to 900 nm. For spectral mode (iCCD), a 150 g mm^{-1} grating, blazed at 500 nm is used, offering a wavelength coverage of 540 nm over the full wavelength range extending from 250 to 900 nm. Single-wavelength absorption changes were monitored using a PMT LP detector (Hamamatsu R928), which covers the spectral range from 185 to 870 nm. The probe was a 150 W ozone-free xenon short arc lamp (OSRAM XBO 150W/CR OFR) that was pulsed at the same frequency of the laser. In all cases, the concentration at the excitation wavelength was adjusted to reach absorbance values between 0.1 and 0.5. All measurements were performed in argon-purged solution at room temperature.

Electrochemistry. Cyclic voltammetry and differential pulse voltammetry was recorded on an Autolab PGSTAT 100 potentiostat using a standard three-electrode-cell containing a glassy carbon disk of approximately 0.03 cm^2 area (working electrode), a platinum wire (counter electrode) and an aqueous Ag/AgCl reference electrode with a salt bridge of 3 M KCl/saturated AgCl. The scan rate is 100 mV/s and two scans were performed. Experiments were performed in degassed, dry acetonitrile containing 0.1 M tetrabutylammonium hexafluorophosphate electrolyte and 1 mM of redox active species. The reported values were determined from half wave potential ($E_{1/2}$) or from the anodic or cathodic peak for reversible and irreversible redox events respectively.

Spectroelectrochemistry. Spectroelectrochemistry measurements were carried out in an OTTLE cell.[3-5] The cell consists of a demountable cell body equipped with a set of CaF_2 optical windows (41×23×6 mm) allowing to perform UV-Vis-NIR-IR (down to 1100 cm^{-1}) spectroelectrochemical experiments. The windows are separated with a modified polyethylene spacer (optical path of ~0.2 mm) with a melt-sealed three-electrode system consisting of a Pt minigrid working electrode (32 wires/cm), a Pt minigrid auxiliary electrode and an Ag wire pseudo-reference electrode. The electrode set is housed in a solvent-resistant polyoxomethylene (POM) frame. The wire contacts are stable against air oxidation and shielded from electronic noise. Electrochemical potential was controlled using an EmStat4S LR potentiostat from Palmsens. UV-Visible spectra at different applied potentials were recorded on the previously described Agilent setup.

II. Synthesis and Characterization

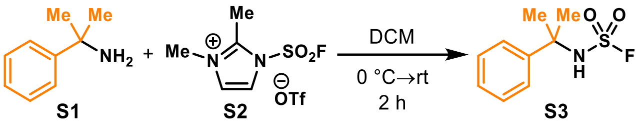

Cumyl sulfamoyl fluoride **S3** was prepared following our previously reported protocol.[6]

Synthesis of Symmetrical Diazene **1**:

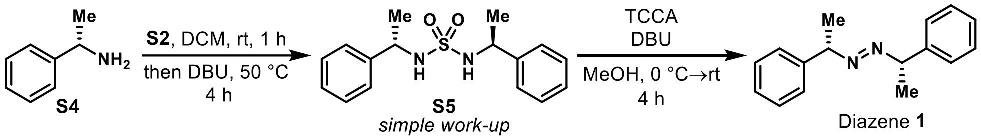

Diazene **1** was prepared following our previously reported protocol with slight modification.[7]

To a flame-dried 100 mL round-bottom-flask equipped with a PTFE-coated stir bar was added (*S*)-(–)-1-phenylethylamine (218 mg, 2.1 equiv, 1.8 mmol) under argon, followed by anhydrous MeCN (7 mL, C = 0.25 M). The mixture was cooled to 0 °C with an ice-bath, and **SuFEx-It** reagent **S2** (281 mg, 1.0 equiv, 0.9 mmol) was added quickly to the solution in one portion. The reaction mixture was allowed to reach room temperature (~5-10 min) and then stirred for 1 hour. 1,8-diazabicyclo(5.4.0)undec-7-ene (DBU, 0.13 mL, 0.9 mmol, 1.0 equiv) was subsequently added dropwise over 1 minute and then the reaction mixture was stirred at 50 °C for 4 h. The progression of the reaction was monitored by TLC. Upon completion, the reaction was quenched by the addition of *aq.* HCl (C = 1 M, ~20 mL). The mixture was extracted with EtOAc (~3×20 mL), and the combined organic layers were washed with brine (~20 mL), dried over Na_2SO_4 and then filtered. The solvent was evaporated *in vacuo* and the crude sulfamide **S5** was subjected to the next step without further purification.

The crude sulfamide **S5** (487 mg, 1.6 mmol, 1.0 equiv) was directly taken in a 50 mL round-bottom flask that had been flame-dried and had a stir bar coated with PTFE. After being evacuated, the flask

was put under Ar. After adding DBU (1.2 mL, 8.0 mmol, 5.0 equiv) and dry MeOH (11.0 mL, C = 0.15 M), the mixture was cooled to 0 °C using an ice bath. A solution of trichloroisocyanuric acid (TCCA, 409 mg, 1.8 mmol, 1.1 equiv) in dry MeOH (6.0 mL, C = 0.30 M) under argon was added using a syringe for five minutes at 0 °C. After that, the reaction mixture was brought to room temperature and agitated until TLC indicated full conversion. The volatiles were removed in vacuo with the temperature of the rotary evaporator bath kept below 30 °C, and the resulting residues were purified by column chromatography (SiO_2, 5:95 EtOAc:hexanes) to afford diazene **1** as a pale-yellow oil (340 mg, 79% yield over two steps).

The spectroscopic data for this compound was identical to those reported in the literature.[7]

^{1}H NMR ($CDCl_3$, 600 MHz) δ: 7.43 – 7.12 (m, 10 H), 4.64 (q, J = 6.9 Hz, 2 H), 1.56 (d, J = 6.8 Hz, 6 H).

Synthesis of Unsymmetrical Cumyl Diazenes: General procedure A

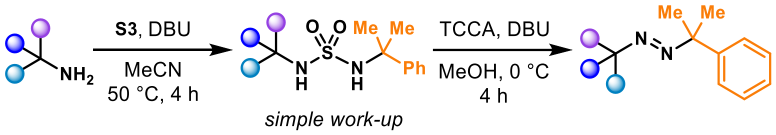

Diazenes **2, 3** and **S6, S7** were all synthesized following our previously reported protocol.[6]

The amine (1.0 equiv) and cumyl sulfamoyl fluoride **S2** (1.0 equiv) were added to a flame-dried 100 mL round-bottom flask equipped with a PTFE-coated stir bar under argon. This was followed by the addition of anhydrous MeCN (C ~ 0.15 M). [Liquid amines were introduced directly to the flask following the addition of the solvent]. 1,8-Diazabicyclo[5.4.0]undec-7-ene (DBU, 1.0 equiv) was subsequently added dropwise over one minute and the reaction mixture was warmed to 50 °C and allowed to stir for 4 h. The progression of the reaction was monitored by TLC. Upon completion (typically about 4 h), the reaction was quenched by the addition of *aq.* HCl (C = 1 M, ~20 mL). The mixture was extracted with EtOAc (~3×20 mL), and the combined organic layers were washed with brine (~20 mL), dried over Na_2SO_4, and then filtered. The solvent was evaporated *in vacuo* and the crude sulfamide was subjected to the next step without further purification.

The sulfamide precursor (1.0 equiv) was placed in a flame-dried 50 mL round-bottom flask equipped with a PTFE-coated stir bar. After evacuating the flask and purging it with argon, dry MeOH (C ≈ 0.15 M) and DBU (5.0 equiv) were added. The mixture was then cooled to 0 °C with an ice bath. A solution of trichloroisocyanuric acid (TCCA, 1.1 equiv) in dry MeOH (C ≈ 0.30 M) under argon was added to the ice-cold reaction mixture using a syringe over 5 min. The flask was then warmed to room temperature and allowed to stir until full conversion was reached as confirmed by TLC (typically about 2–4 h). The volatiles were removed *in vacuo* with the temperature of the rotary evaporator bath kept

below 30 °C, and the resulting residues were directly purified by silica gel column chromatography to afford the desired product.

Note: All yields below were calculated from the starting amines or their corresponding hydrochloride salts. While we have never observed any explosive or uncontrolled reactions with this procedure, using a blast shield is recommended because of the high reactivity of diazenes. All diazenes were stored at –20 °C in the dark. No decomposition was observed in these conditions over the course of several weeks. Slow decomposition was observed at room temperature over the course of several days.

Synthesis and characterization of cumyldiazenes

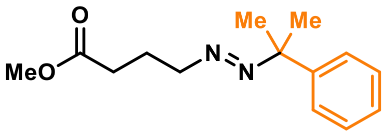

Diazene **2** was prepared from methyl 4-aminobutyrate hydrochloride (364 mg, 2.37 mmol) following **general procedure A**. (2 equiv of DBU were used to synthesize the sulfamide precursor instead of 1 equiv). Column chromatography (SiO_2, 0:100 to 20:80 EtOAc:hexanes) afforded **2** as a pale-yellow oil (376 mg, 64%).

The spectroscopic data for this compound were identical to those reported in the literature.[6]

^{1}H NMR ($CDCl_3$, 400 MHz) δ: 7.39 – 7.18 (m, 5 H), 3.87 (t, *J* = 6.9 Hz, 2 H), 3.67 (s, 3 H), 2.41 (t, *J* = 7.5 Hz, 2 H), 2.14 (p, *J* = 7.4, 7.0 Hz, 2 H), 1.50 (s, 6 H).

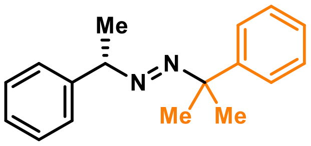

Diazene **3** was prepared from (*S*)-1-phenyl-1-ethanamine (243 mg, 2.00 mmol) following **general procedure A**. Column chromatography (SiO_2, 0:100 to 5:95 EtOAc:hexanes) afforded **3** as a colorless oil (400 mg, 79%).

The spectroscopic data for this compound were identical to those reported in the literature.[6]

^{1}H NMR (CD_3CN, 500 MHz) δ: 7.45–7.22 (m, 10 H, *overlaps with* $CDCl_3$), 4.70 (q, *J* = 6.8 Hz, 1 H), 1.54 (d, *J* = 6.9 Hz, 3 H), 1.50 (s, 3 H), 1,47 (s, 3 H) ppm.

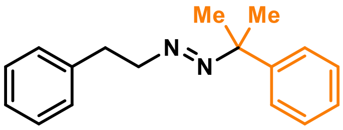

Diazene **S6** was prepared from phenethylamine (500 mg, 4.13 mmol) following **general procedure A**. Column chromatography (SiO_2, 0:100 to 5:95 EtOAc:hexanes) afforded **S6** as a pale-yellow oil (864 mg, 83%).

The spectroscopic data for this compound were identical to those reported in the literature.[6]

^{1}H NMR ($CDCl_3$, 400 MHz) δ: 7.31–7.13 (m, 10 H, *overlaps with* $CDCl_3$), 4.13 (t, *J* = 7.4 Hz, 2 H), 3.11 (t, *J* = 7.4 Hz, 2 H), 1.45 (s, 6 H) ppm.

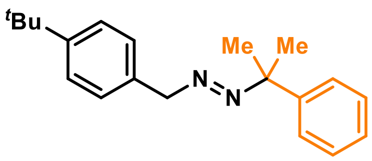

Diazene **S7** was prepared from 4-*tert*-butylbenzylamine (163 mg, 1.00 mmol) following **general procedure A**. Column chromatography (SiO_2, 0:100 to 5:95 EtOAc:hexanes) afforded **S7** as a colorless oil (159 mg, 54%).

The spectroscopic data for this compound were identical to those reported in the literature.[6]

^{1}H NMR ($CDCl_3$, 400 MHz) δ: 7.46–7.38 (m, 6 H), 7.33–7.23 (m, 3 H, *overlaps with $CDCl_3$*), 4.89 (s, 2 H), 1.45 (s, 6 H), 1.24 (s, 9 H).

Diazene fragmentation and cross-coupling reaction: General procedure B

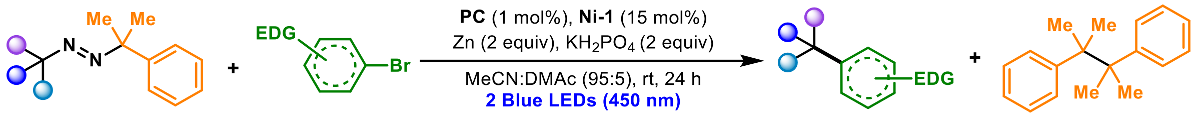

A flame-dried and argon-purged 4 mL vial equipped with a bean-shaped PTFE-coated stir bar was charged with aryl bromide **7** (0.19 mmol, 1.5 equiv), photocatalyst **PC-6 or PC-10** (1 mol%), $NiBr_2$•(dtbbpy) (**Ni-1**) (8.2 mg, 15 mol%), KH_2PO_4 (34 mg, 0.25 mmol, 2.0 equiv), and activated zinc powder[2] (16.5 mg, 0.25 mmol, 2.0 equiv). Upon addition of all the solids, the vial was evacuated under high vacuum for 15 min and then backfilled with argon. Anhydrous degassed MeCN (0.57 mL) was added with a syringe under an argon atmosphere followed by anhydrous degassed DMAc (0.03 mL) and diazene. The vial was then irradiated using two blue LEDs (30 W, 450 nm) in the Hepatochem photoreactor, while the reaction was stirred at 350 rpm. After 24 h of irradiation, the reaction mixture was diluted with EtOAc (2 mL) and filtered through a silica plug (~2.0 cm), which was subsequently flushed with additional EtOAc (50 mL). Following concentration of the volatiles *in vacuo,* NMR yields were measured by addition of $PhSiMe_3$ as an internal standard. For each substrate, isolated yields were measured with the optimal PC after purification by column chromatography (see below).

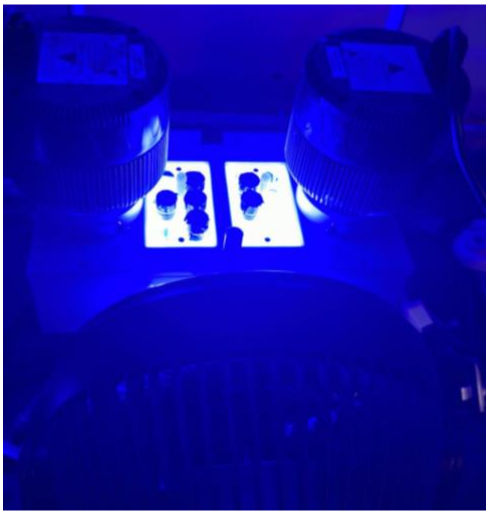

Figure S1: Photoreaction setup: Hepatochem EvoluChem™ PhotoRedOx Box Duo with light sources (EvoluChem™ P303-30-1 LEDs) and vial holders.

Synthesis and characterization of cross-coupling products

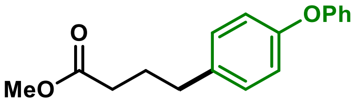

8a was prepared from **2** (31 mg, 0.13 mmol) and **7a** (47 mg, 0.19 mmol) following **general procedure B**. Column chromatography (SiO_2, 0:100 to 10:90 EtOAc:hexanes) afforded **8a** as a pale yellow oil (17 mg, 48%) when **PC-6** was used as the photocatalyst.

R_f = 0.46 (10:90 EtOAc:hexanes)

^{1}H NMR ($CDCl_3$, 400 MHz) δ: 7.32 (dd, J = 8.6, 7.3 Hz, 2 H), 7.14 (d, J = 8.5 Hz, 2 H), 7.11 – 7.05 (m, 1 H), 6.99 (dd, J = 8.7, 1.1 Hz, 2 H), 6.94 (d, J = 8.5 Hz, 2 H), 3.68 (s, 3 H), 2.64 (t, J = 7.6 Hz, 2 H), 2.35 (t, J = 7.5 Hz, 2 H), 1.96 (p, J = 7.5 Hz, 2 H).
^{13}C NMR ($CDCl_3$, 126 MHz) δ: 174.1, 157.7, 155.4, 136.5, 129.8, 123.1, 119.2, 118.7, 51.7, 34.5, 33.5, 26.7 ppm.
HRMS(+ESI) calc'd for $C_{19}H_{22}O$ $[M+H]^+$ 271.1329, found 271.1327.

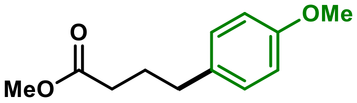

8b was prepared from **2** (31 mg, 0.13 mmol) and 4-Bromoanisole (**7b**) (35 mg, 0.19 mmol) following **general procedure B**. Column chromatography (SiO_2, 0:100 to 10:90 EtOAc:hexanes) afforded **8b** as a colorless oil (14 mg, 52%) when **PC-6** was used as the photocatalyst.

The spectroscopic data for this compound were identical to those reported in the literature.[8]

^{1}H NMR ($CDCl_3$, 400 MHz) δ: 7.09 (d, J = 8.5 Hz, 2 H), 6.83 (d, J = 8.6 Hz, 2 H), 3.79 (s, 3 H), 3.66 (s, 3 H), 2.59 (t, J = 7.6 Hz, 2 H), 2.32 (t, J = 7.5 Hz, 2 H), 1.92 (p, J = 7.5 Hz, 2 H).

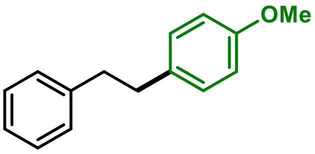

8c was prepared from **S6** (32 mg, 0.13 mmol) and 4-Bromoanisole (**7b**) (35 mg, 0.19 mmol) following **general procedure B**. Column chromatography (SiO_2, 0:100 to 3:97 EtOAc:hexanes) afforded **8c** as a white solid (11 mg, 40%) when **PC-6** was used as the photocatalyst.

The spectroscopic data for this compound were identical to those reported in the literature.[9]

^{1}H NMR ($CDCl_3$, 400 MHz) δ: 7.31–7.26 (m, 2 H), 7.23–7.15 (m, 3 H), 7.12–7.06 (m, 2 H), 6.86–6.78 (m, 2 H), 3.79 (s, 3 H), 2.93–2.82 (m, 4 H).

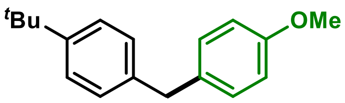

8d was prepared from **S7** (37 mg, 0.13 mmol) and 4-Bromoanisole (**7b**) (35 mg, 0.19 mmol) following **general procedure B**. Column chromatography (SiO_2, 0:100 to 3:97 EtOAc:hexanes) afforded **8d** as a colorless oil (18 mg, 54%) when **PC-6** was used as the photocatalyst.

The spectroscopic data for this compound were identical to those reported in the literature.[10]

^1H NMR ($CDCl_3$, 400 MHz) δ: 7.30 (d, J = 8.3 Hz, 2 H), 7.18–7.04 (m, 4 H), 6.83 (d, J = 8.7 Hz, 2 H), 3.90 (s, 2 H), 3.79 (s, 3 H), 1.30 (s, 9 H).

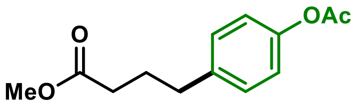

8e was prepared from **2** (31 mg, 0.13 mmol) and 4-Bromophenyl acetate (**7c**) (41 mg, 0.19 mmol) following **general procedure B**. Column chromatography (SiO_2, 0:100 to 10:90 EtOAc:hexanes) afforded **8e** as a colorless oil (13 mg, 44%) when **PC-6** was used as the photocatalyst.

The spectroscopic data for this compound were identical to those reported in the literature.[11]

^1H NMR ($CDCl_3$, 400 MHz) δ: 7.18 (d, J = 8.4 Hz, 2 H), 6.99 (d, J = 8.5 Hz, 2 H), 3.67 (s, 3 H), 2.64 (t, J = 7.6 Hz, 2 H), 2.34 (t, J = 7.4 Hz, 2 H), 2.29 (s, 3 H), 1.95 (p, J = 7.5 Hz, 2 H).

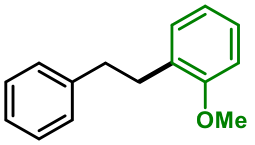

8f was prepared from **S6** (32 mg, 0.13 mmol) and 2-Bromoanisole (**7d**) (35 mg, 0.19 mmol) following **general procedure B**. Column chromatography (SiO_2, 0:100 to 5:95 EtOAc:hexanes) afforded **8f** as a colorless oil (12 mg, 44%) when **PC-6** was used as the photocatalyst.

The spectroscopic data for this compound were identical to those reported in the literature.[12]

^1H NMR ($CDCl_3$, 400 MHz) δ: 7.33–7.15 (m, 6 H, overlaps with $CDCl_3$), 7.13–7.07 (m, 1 H), 6.90 – 6.82 (m, 2 H), 3.82 (s, 3 H), 2.96–2.83 (m, 4 H).

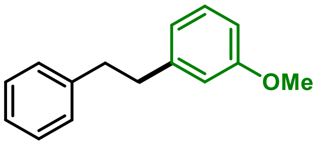

8g was prepared from **S6** (32 mg, 0.13 mmol) and 3-Bromoanisole (**7e**) (35 mg, 0.19 mmol) following **general procedure B**. Column chromatography (SiO_2, 0:100 to 2:98 EtOAc:hexanes) afforded **8g** as a colorless oil (12 mg, 45%) when **PC-10** was used as the photocatalyst.

The spectroscopic data for this compound were identical to those reported in the literature.[13]

^1H NMR ($CDCl_3$, 400 MHz) δ: 7.32–7.27 (m, 2 H), 7.23–7.17 (m, 4 H), 6.82–6.71 (m, 3 H), 3.79 (s, 3 H), 2.97–2.87 (m, 4 H).

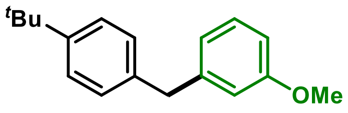

8h was prepared from **S7** (37 mg, 0.13 mmol) and 3-Bromoanisole (**7e**) (35 mg, 0.19 mmol) following **general procedure B**. Column chromatography (SiO_2, 0:100 to 2:98 EtOAc:hexanes) afforded **8h** as a colorless oil (18 mg, 56%) when **PC-10** was used as the photocatalyst.

The spectroscopic data for this compound were identical to those reported in the literature.[14]

^{1}H NMR ($CDCl_3$, 400 MHz) δ: 7.32–7.28 (m, 2 H), 7.23–7.16 (m, 1 H), 7.14–7.09 (m, 2 H), 6.82–6.77 (m, 1 H), 6.73–6.75 (m, 2 H), 3.92 (s, 2 H), 3.78 (s, 3 H), 1.30 (s, 9 H).

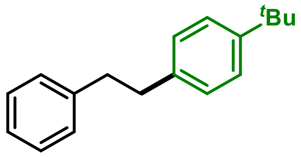

8i was prepared from **S6** (32 mg, 0.13 mmol) and 1-Bromo-4-tert-butylbenzene (**7f**) (40 mg, 0.19 mmol) following **general procedure B**. Purification by preparative TLC (2:98 Et_2O:hexanes, eluted twice) afforded **8i** as a white solid (15 mg, 50%) when **PC-10** was used as the photocatalyst.

The spectroscopic data for this compound were identical to those reported in the literature.[15]

^{1}H NMR ($CDCl_3$, 500 MHz) δ: 7.35–7.31 (m, 2 H), 7.31–7.27 (m, 2 H), 7.24–7.20 (m, 2 H), 7.20–7.15 (m, 3 H), 2.98–2.86 (m, 4 H), 1.33 (s, 9 H).

Photocatalyst screen for cross-coupling with an electron-poor aryl bromide:

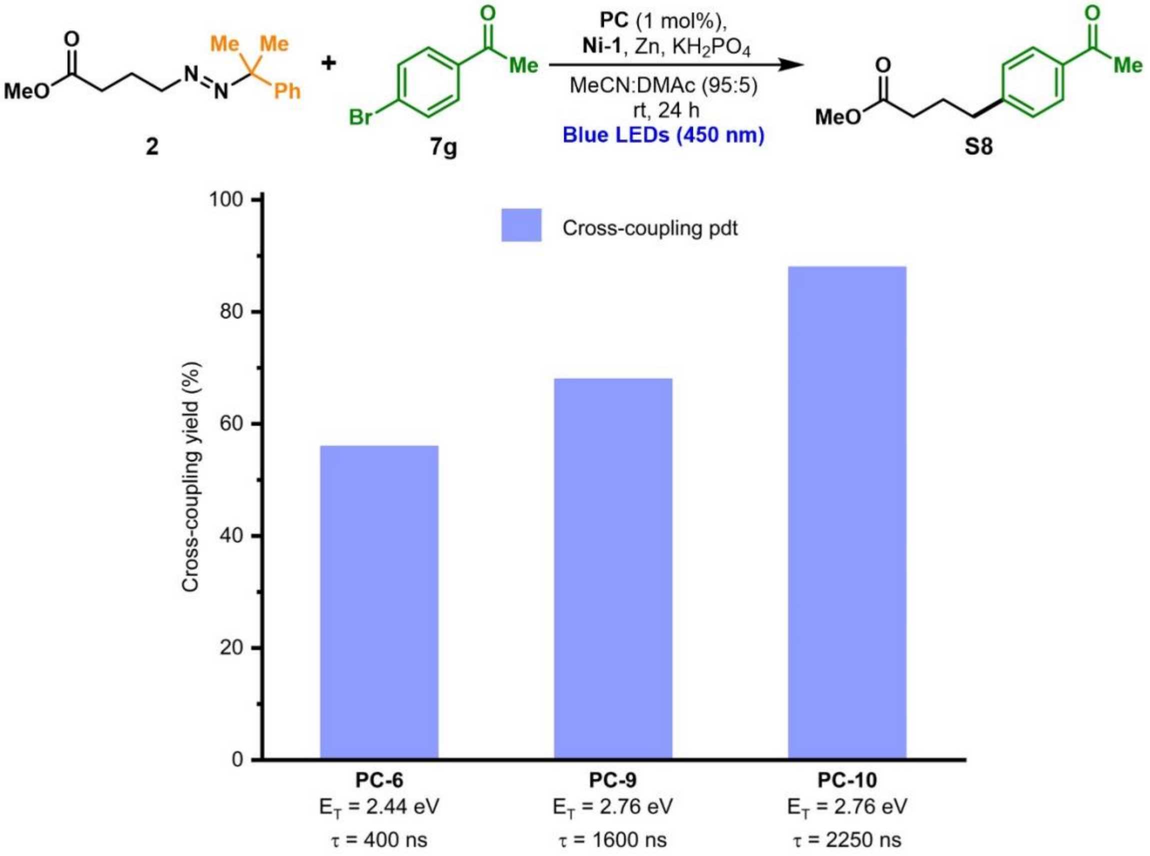

Figure S2: Evaluation of photocatalyst efficiency in the cross-coupling reaction between diazene **2** and electron-deficient 4-Bromoacetophenone (**7g**).

III. Absorption and Emission Spectroscopies

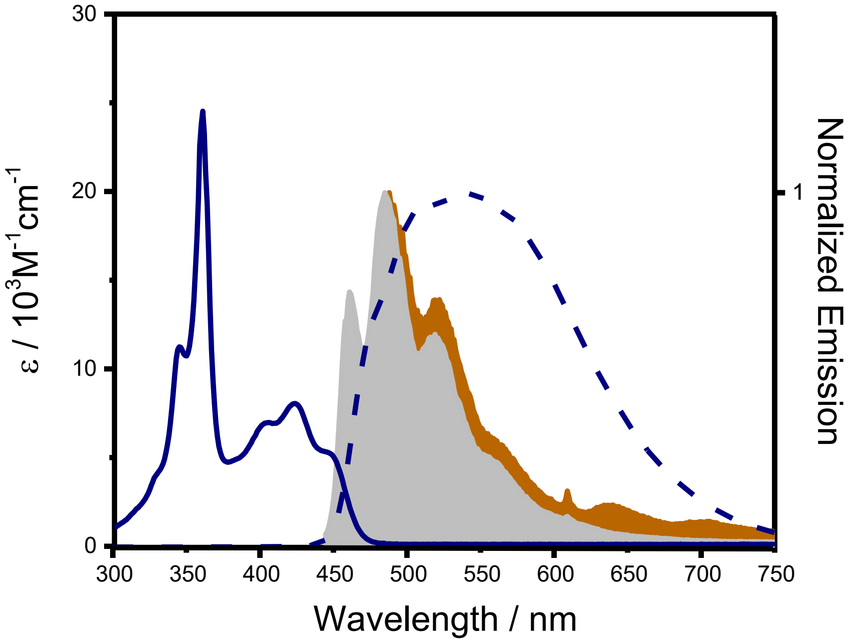

Figure S3: UV-vis absorption (dark blue, solid line) and emission spectra of (dark blue, dashed line) Acr-Me (**PC-1**) recorded in degassed acetonitrile at room temperature. 77K emission spectra of Acr-Me (**PC-1**) recorded in butyronitrile without (pale grey) and with iodomethane (dark orange).

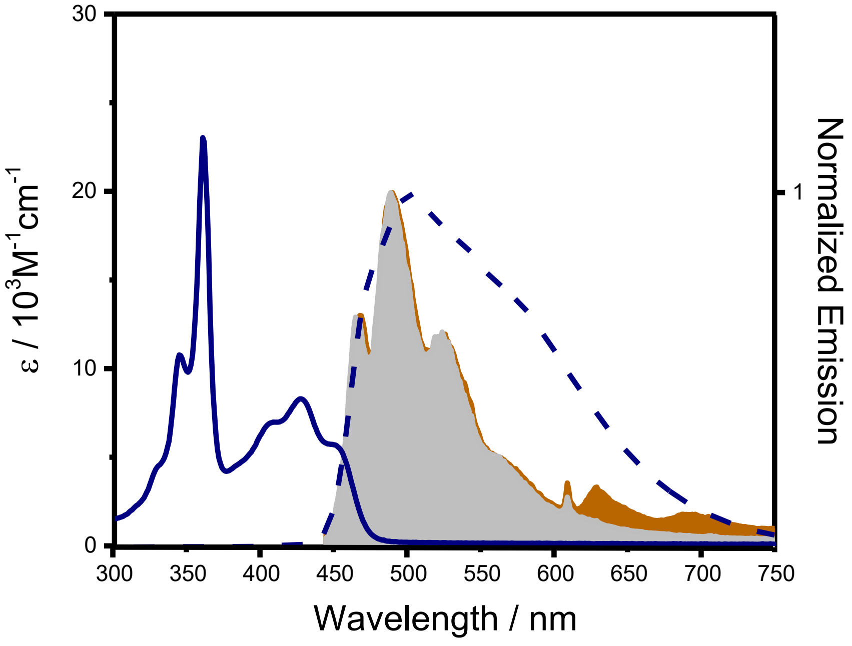

Figure S4: UV-vis absorption (dark blue, solid line) and emission spectra of (dark blue, dashed line) Acr-Ph (**PC-2**) recorded in degassed acetonitrile at room temperature. 77K emission spectra of Acr-Ph (**PC-2**) recorded in butyronitrile without (pale grey) and with iodomethane (dark orange).

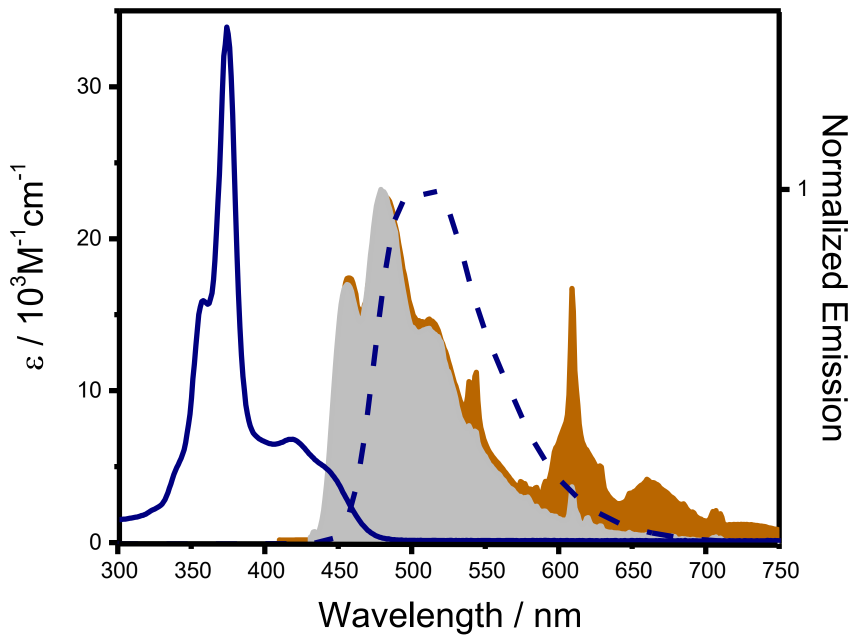

Figure S5: UV-vis absorption (dark blue, solid line) and emission spectra of (dark blue, dashed line) Acr-^{t}Bu (**PC-3**) recorded in degassed acetonitrile at room temperature. 77K emission spectra of Acr-^{t}Bu (**PC-3**) recorded in butyronitrile without (pale grey) and with iodomethane (dark orange).

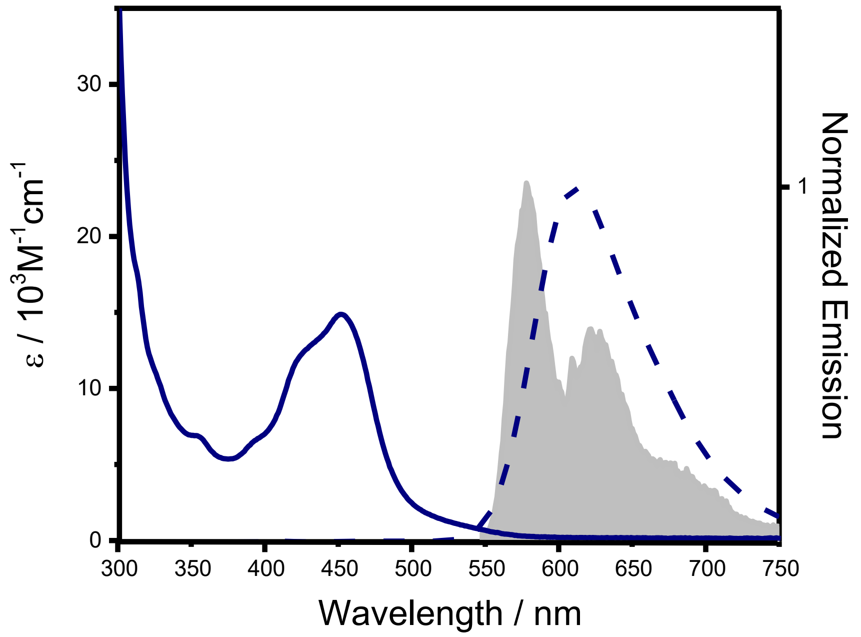

Figure S6: UV-vis absorption (dark blue, solid line) and emission spectra of (dark blue, dashed line) $[Ru(bpy)_3]^{2+}$ (**PC-4**) recorded in degassed acetonitrile at room temperature. 77K emission spectra of $[Ru(bpy)_3]^{2+}$ (**PC-4**) recorded in butyronitrile (pale grey).

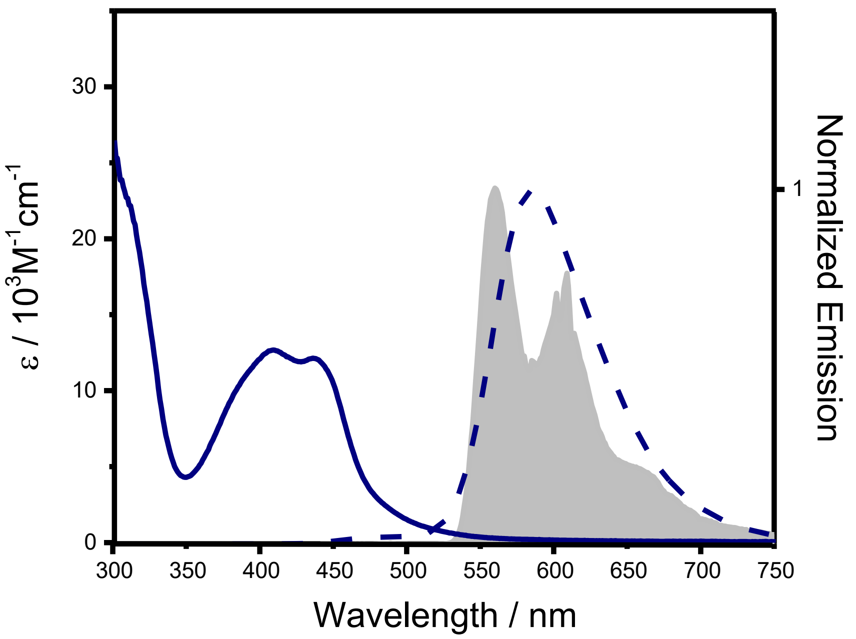

Figure S7: UV-vis absorption (dark blue, solid line) and emission spectra of (dark blue, dashed line) $[Ru(TAP)_3]^{2+}$ (**PC-5**) recorded in degassed acetonitrile at room temperature. 77K emission spectra of $[Ru(TAP)_3]^{2+}$ (**PC-5**) recorded in butyronitrile (pale grey).

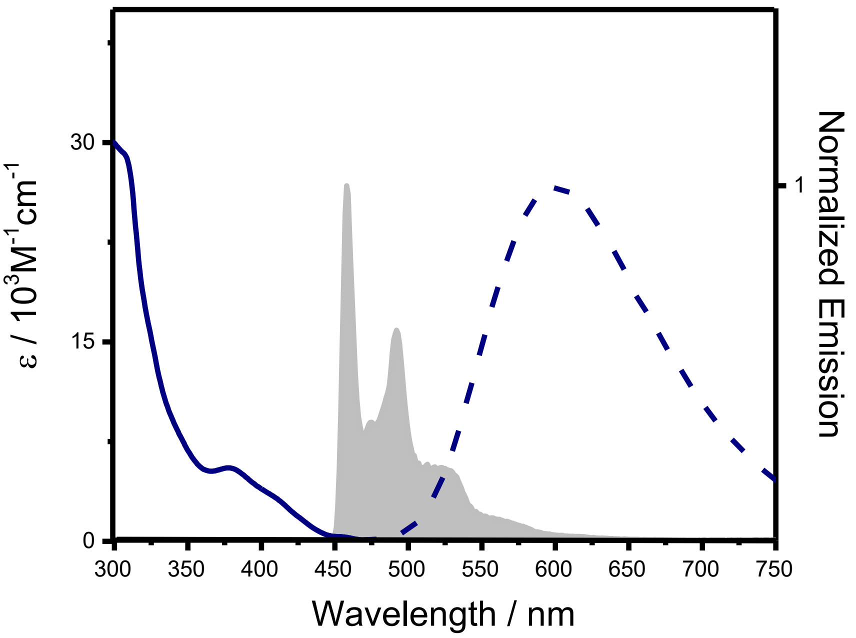

Figure S8: UV-vis absorption (dark blue, solid line) and emission spectra of (dark blue, dashed line) $[Ir(dFCF_3ppy)_2((CF_3)_2bpy)]^+$ (**PC-6)** recorded in degassed acetonitrile at room temperature. 77K emission spectra of $[Ir(dFCF_3ppy)_2((CF_3)_2bpy)]^+$ (**PC-6)** recorded in butyronitrile (pale grey).

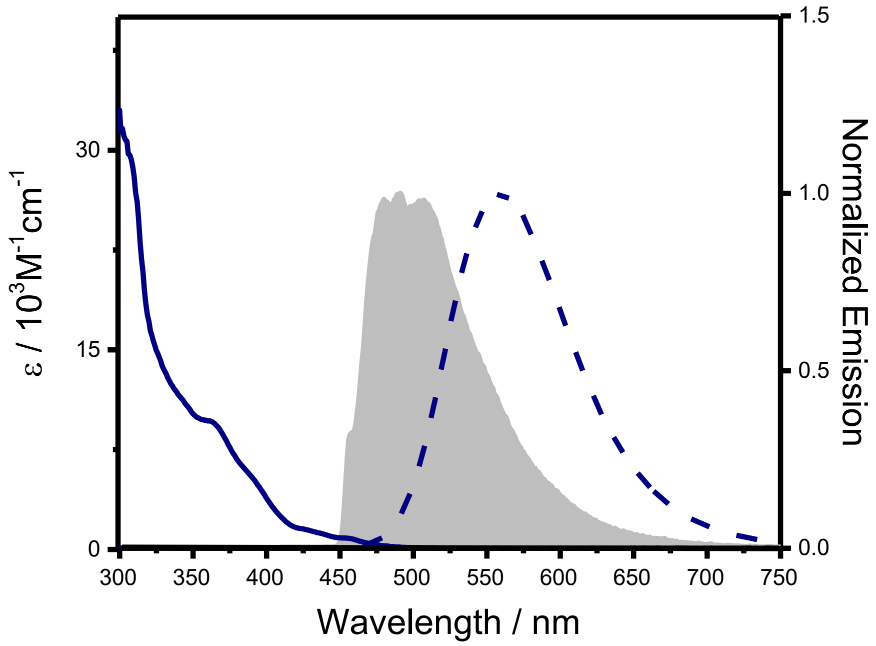

Figure S9: UV-vis absorption (dark blue, solid line) and emission spectra of (dark blue, dashed line) [Ir(Fppy)$_2$(bpy)]$^+$ (**PC-7**) recorded in degassed acetonitrile at room temperature. 77K emission spectra of [Ir(Fppy)$_2$(bpy)]$^+$ (**PC-7**) recorded in butyronitrile (pale grey).

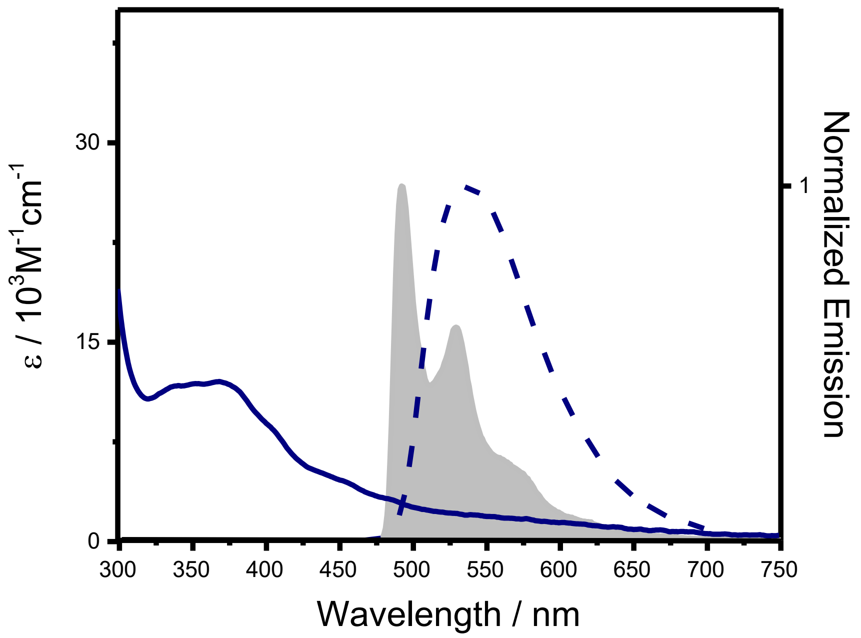

Figure S10: UV-vis absorption (dark blue, solid line) and emission spectra of (dark blue, dashed line) [Ir(ppy)$_3$] (**PC-8**) recorded in degassed acetonitrile at room temperature. 77K emission spectra of [Ir(ppy)$_3$] (**PC-8**) recorded in butyronitrile (pale grey).

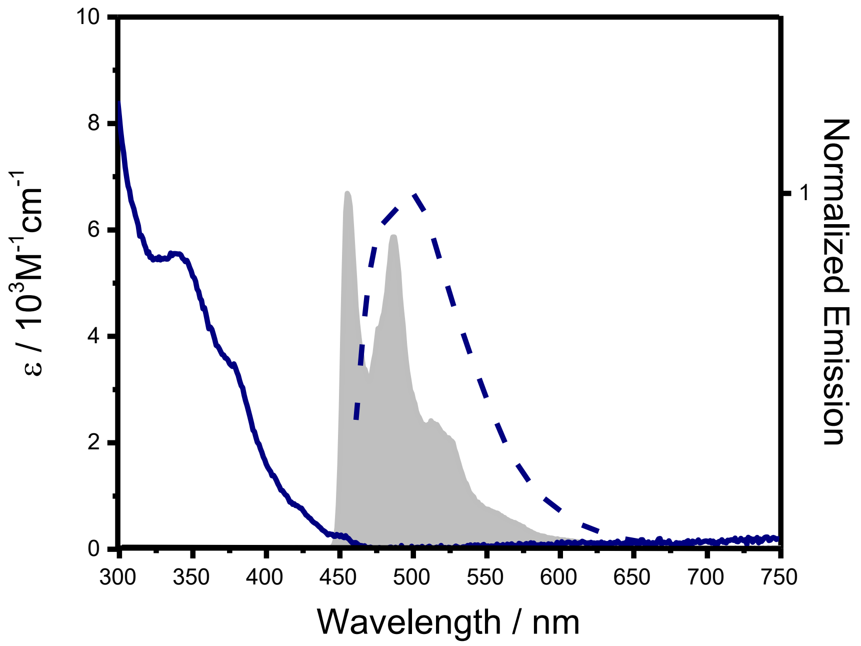

Figure S11: UV-vis absorption (dark blue, solid line) and emission spectra of (dark blue, dashed line) [Ir(dFppy)$_3$] (**PC-9**) recorded in degassed acetonitrile at room temperature. 77K emission spectra of [Ir(dFppy)$_3$] (**PC-9**) recorded in butyronitrile (pale grey).

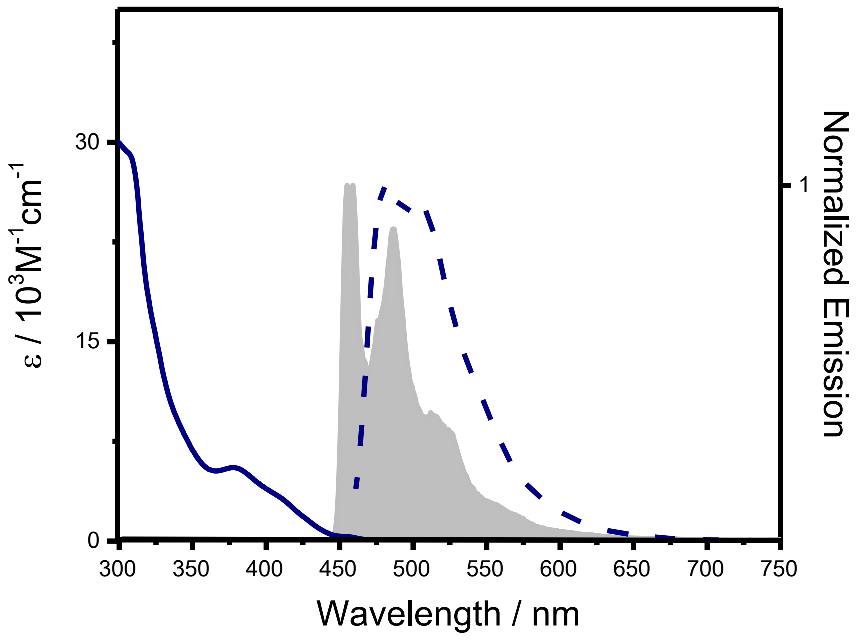

Figure S12: UV-vis absorption (dark blue, solid line) and emission spectra of (dark blue, dashed line) [Ir(dFCF$_3$ppy)$_2$(dtb)]$^+$ (**PC-10**) recorded in degassed acetonitrile at room temperature. 77K emission spectra of [Ir(dFCF$_3$ppy)$_2$(dtb)]$^+$ (**PC-10**) recorded in butyronitrile (pale grey).

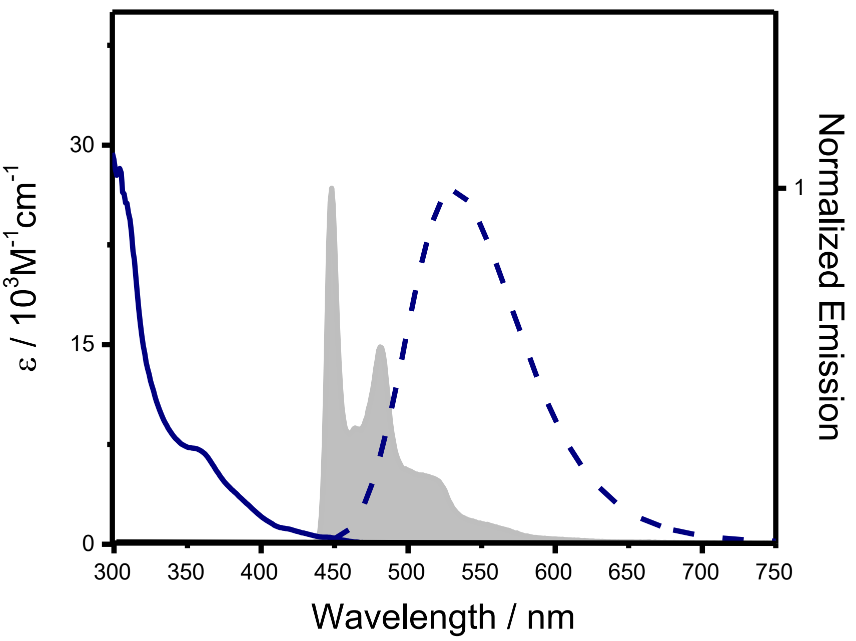

Figure S13: UV-vis absorption (dark blue, solid line) and emission spectra of (dark blue, dashed line) $[Ir(dFppy)_2(bpy)]^+$ (**PC-11**) recorded in degassed acetonitrile at room temperature. 77K emission spectra of $[Ir(dFppy)_2(bpy)]^+$ (**PC-11**) recorded in butyronitrile (pale grey).

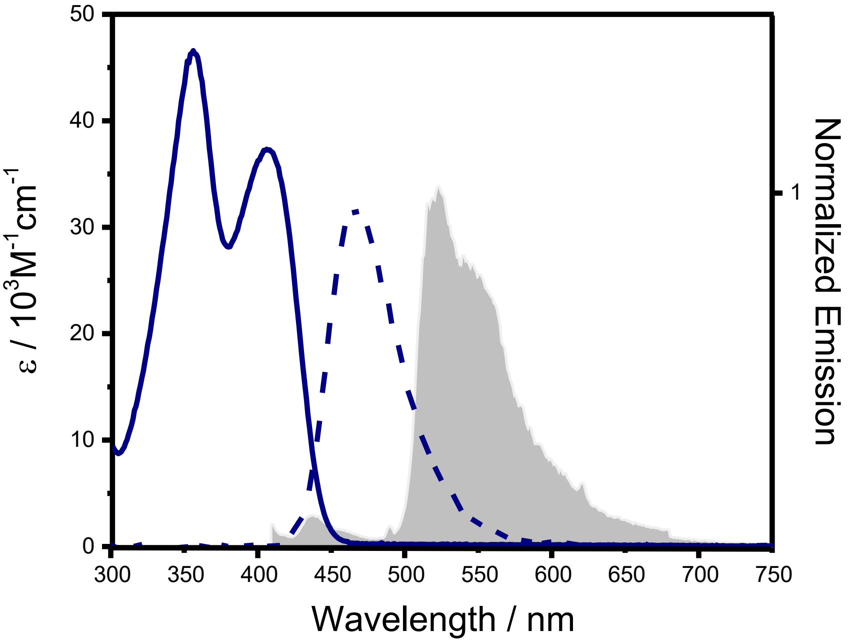

Figure S14: UV-vis absorption (dark blue, solid line) and emission spectra of (dark blue, dashed line) 2,4,6-Triphenylpyrilium (**PC-12**) recorded in degassed acetonitrile at room temperature. 77K emission spectra of 2,4,6-Triphenylpyrilium (**PC-12**) recorded in butyronitrile (pale grey).

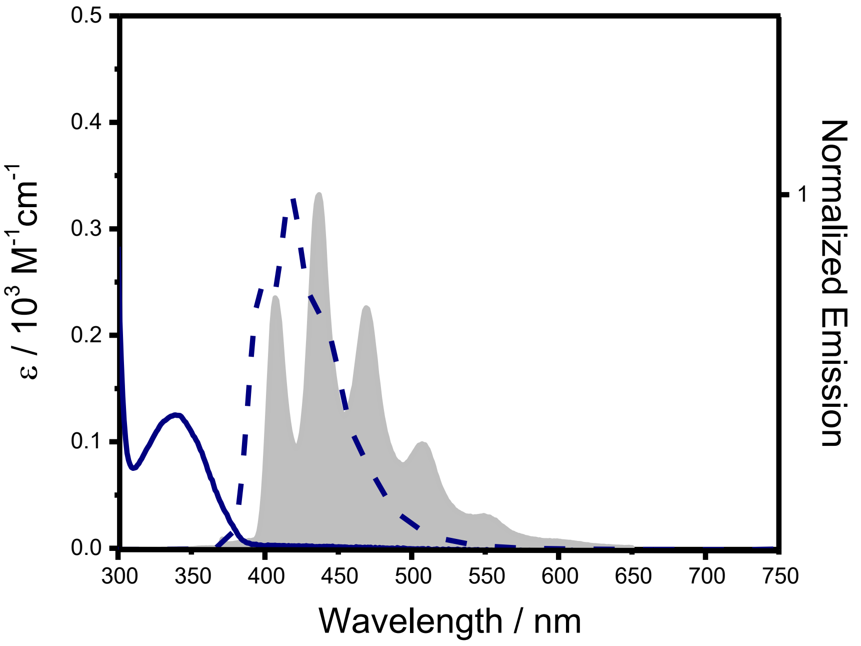

Figure S15: UV-vis absorption (dark blue, solid line) and emission spectra of (dark blue, dashed line) Benzophenone (**PC-13**) recorded in degassed acetonitrile at room temperature. 77K emission spectra of Benzophenone (**PC-13**) recorded in butyronitrile (pale grey).

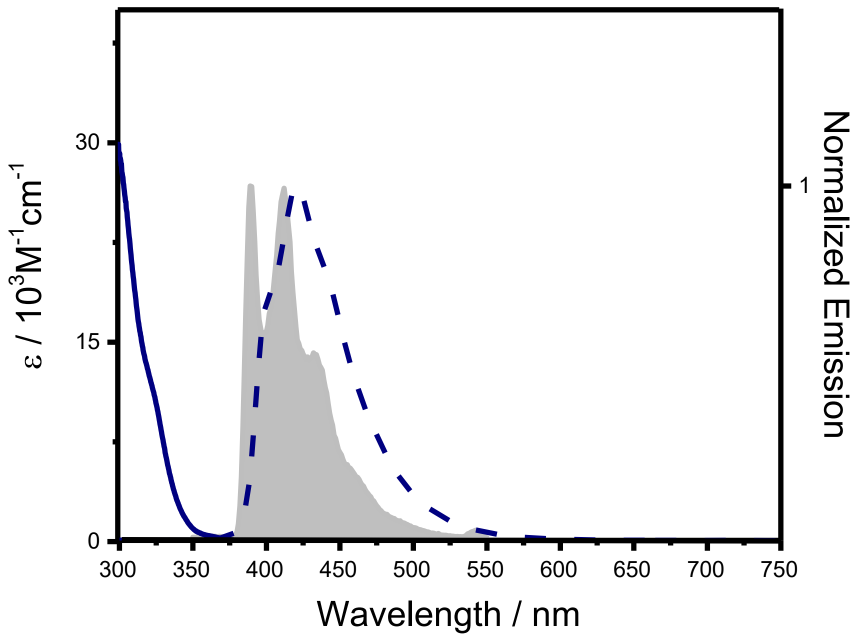

Figure S16: UV-vis absorption (dark blue, solid line) and emission spectra of (dark blue, dashed line) [Ir(CF_3pmb)$_3$] (**PC-14**) recorded in degassed acetonitrile at room temperature. 77K emission spectra of [Ir(CF_3pmb)$_3$] (**PC-14**) recorded in butyronitrile (pale grey).

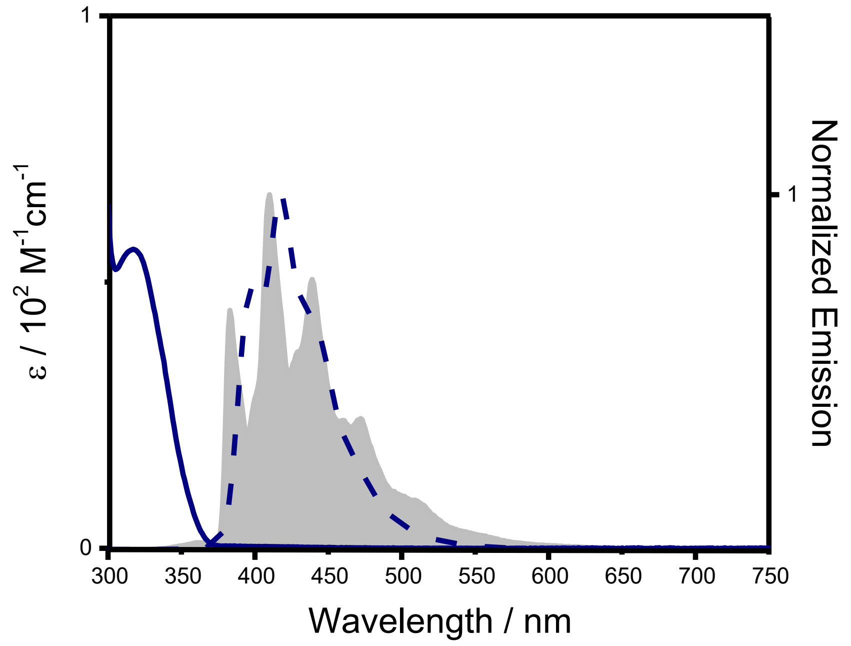

Figure S17: UV-vis absorption (dark blue, solid line) and emission spectra of (dark blue, dashed line) Acetophenone (**PC-15**) recorded in degassed acetonitrile at room temperature. 77K emission spectra of Acetophenone (**PC-15**) recorded in butyronitrile (pale grey).

IV. Electrochemistry

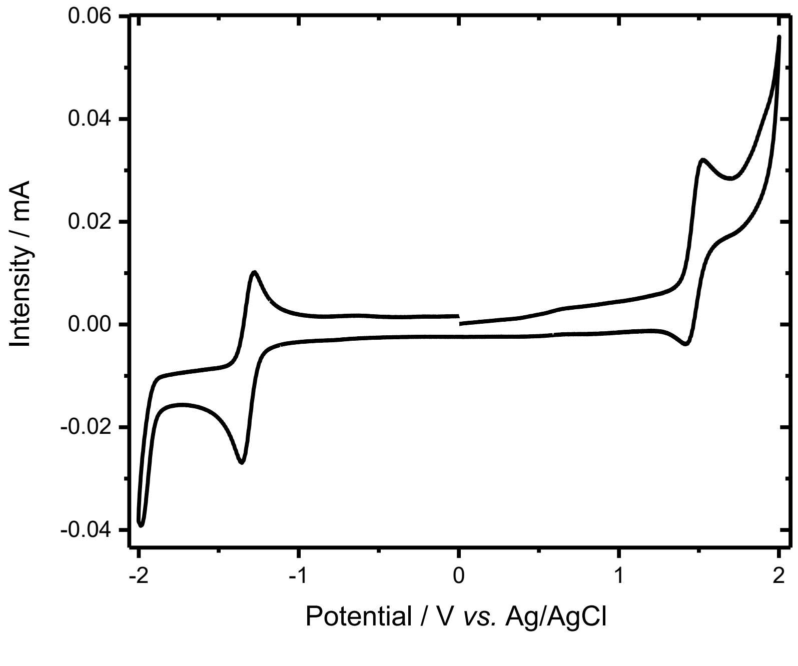

Figure S18: Cyclic voltammetry measurement of $[Ir(Fppy)_2(bpy)]^+$ (**PC-7**) recorded in de-aerated acetonitrile containing 0.1 M $TBAPF_6$ electrolyte.

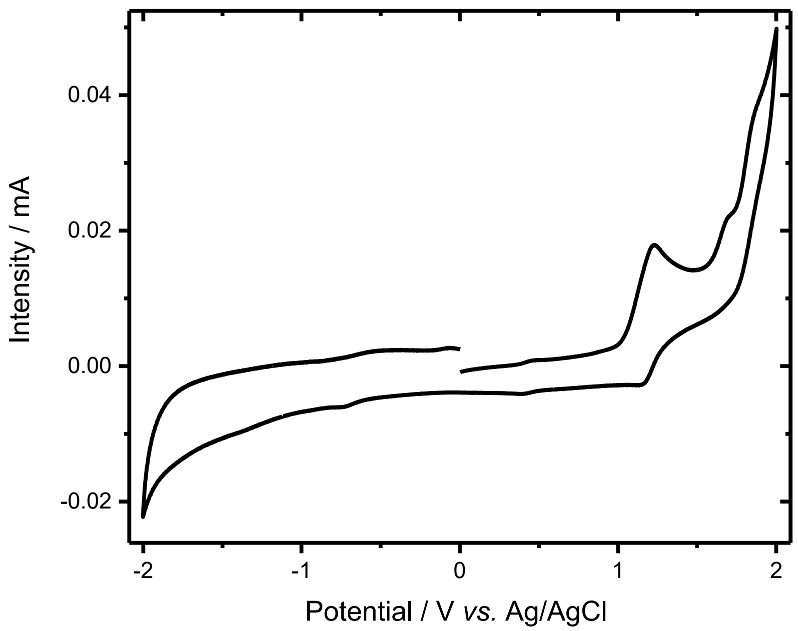

Figure S19: Cyclic voltammetry measurement of [Ir(CF_3pmb)$_3$] (**PC-14**) recorded in de-aerated acetonitrile containing 0.1 M $TBAPF_6$ electrolyte.

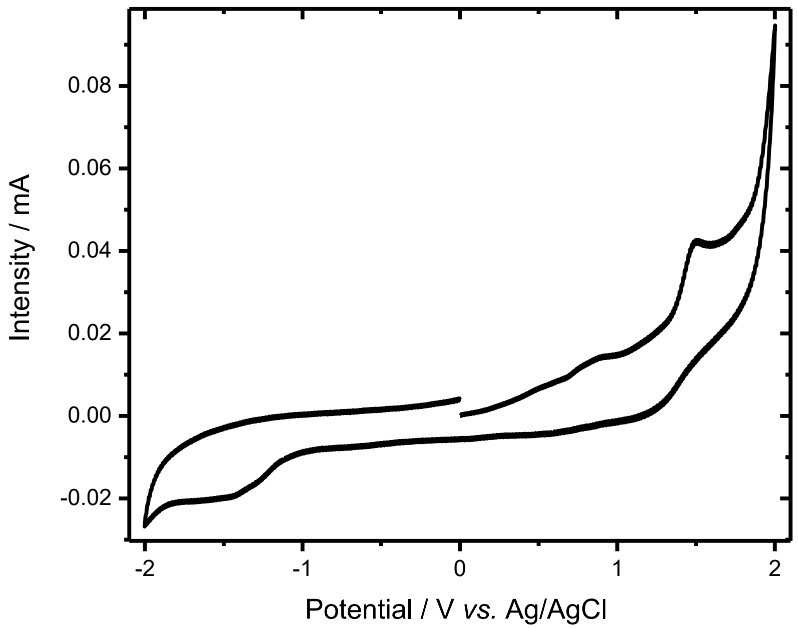

Figure S20: Cyclic voltammetry measurement of **diazene 1** recorded in de-aerated acetonitrile containing 0.1 M $TBAPF_6$ electrolyte.

V. Stern-Volmer Analyses

Quenching measurement with Diazene 1

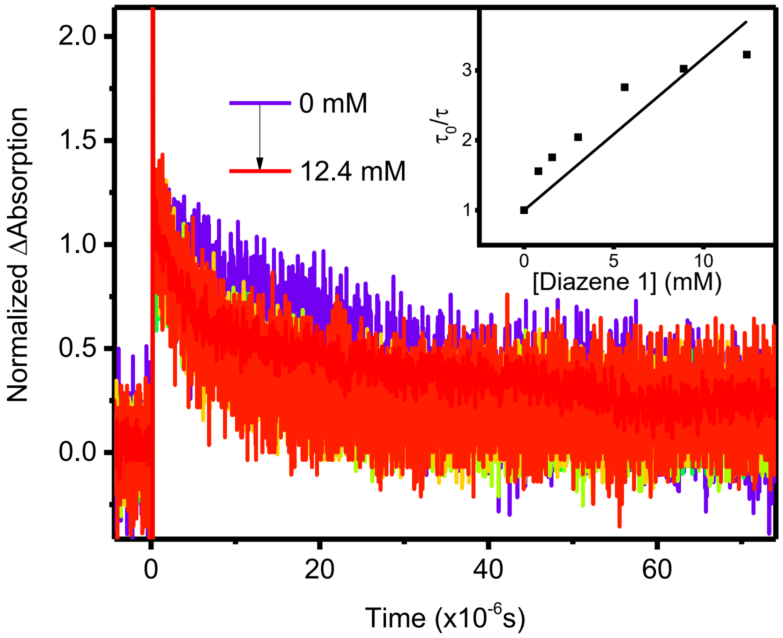

Figure S21: Normalized single wavelength absorption changes of Acr-Me (**PC-1**) (λ_{det} = 495 nm, λ_{exc} = 450 nm) in Ar-saturated acetonitrile in the presence of diazene **1** at different concentrations. Inset: Corresponding Stern-Volmer plot with linear fit from which the quenching rate constant was estimated. For discussion about photoproduct, see section **VII**. Due to presence of photoproduct, the linearity of the Stern-Volmer plot is not ideal. The linear fit has been used to estimate the quenching rate constant.

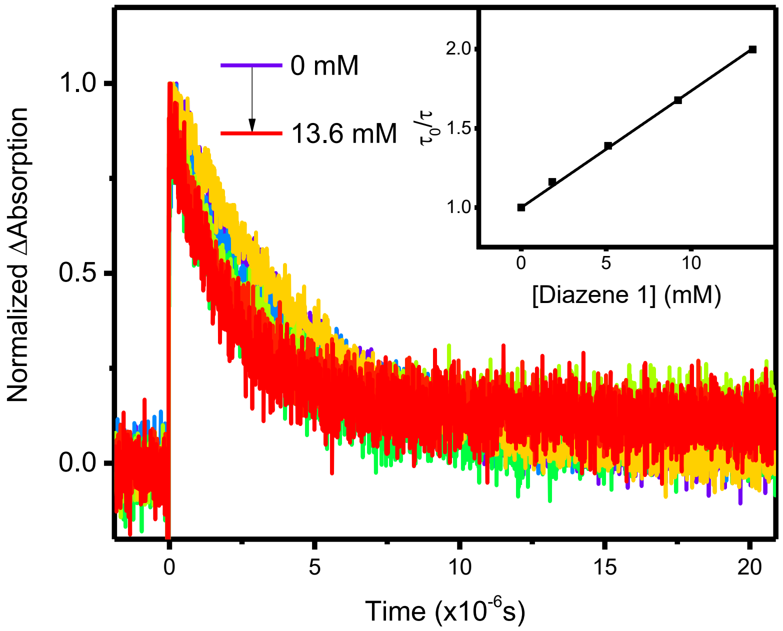

Figure S22: Normalized single wavelength absorption changes of Acr-Ph (**PC-2**) (λ_{det} = 500 nm, λ_{exc} = 450 nm) in Ar-saturated acetonitrile in the presence of diazene **1** at different concentrations. Inset: Corresponding Stern-Volmer plot with linear fit from which the quenching rate constant was estimated. For discussion about photoproduct, see section **VII**.

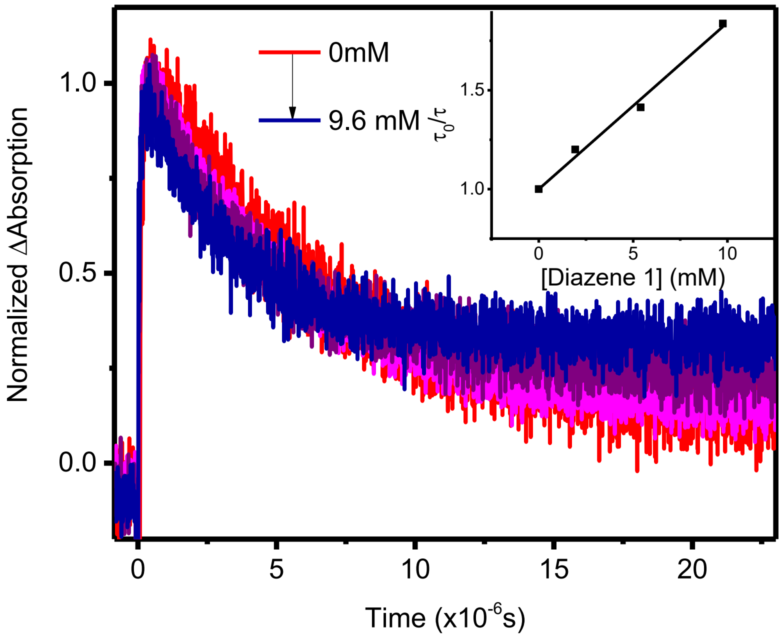

Figure S23: Normalized single wavelength absorption changes of Acr-^{t}Bu (**PC-3**) (λ_{det} = 500 nm, λ_{exc} = 450 nm) in Ar-saturated acetonitrile in the presence of diazene **1** at different concentrations. Inset: Corresponding Stern-Volmer plot with linear fit from which the quenching rate constant was estimated. For discussion about photoproduct, see section **VII**. Due to presence of photoproduct, the linearity of the Stern-Volmer plot is not ideal. The linear fit has been used to estimate the quenching rate constant.

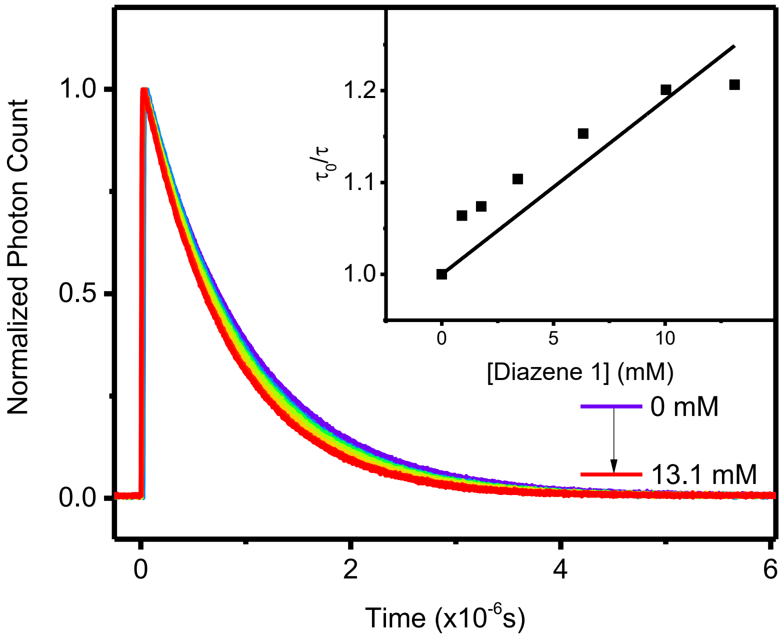

Figure S24: Normalized time-resolved emission measurements of $[Ru(bpy)_3]^{2+}$ (**PC-4**) (λ_{det} = 600 nm, λ_{exc} = 410 nm) recorded in Ar-saturated acetonitrile in the presence of diazene **1** at different concentrations. Inset: Corresponding Stern-Volmer plot with linear fit from which the quenching rate constant was estimated. Due to low quenching, the linearity of the Stern-Volmer plot is not ideal. The linear fit has been used to estimate the quenching rate constant.

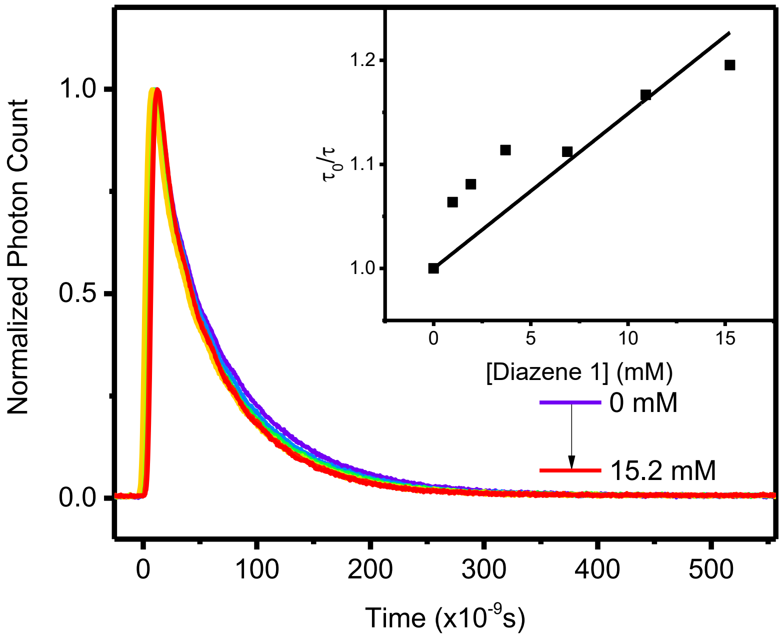

Figure S25: Normalized time-resolved emission measurements of $[Ru(TAP)_3]^{2+}$ (**PC-5**) (λ_{det} = 600 nm, λ_{exc} = 450 nm) recorded in Ar-saturated acetonitrile in the presence of diazene **1** at different concentrations. Inset: Corresponding Stern-Volmer plot with linear fit from which the quenching rate constant was estimated. Due to low quenching, the linearity of the Stern-Volmer plot is not ideal. The linear fit has been used to estimate the quenching rate constant.

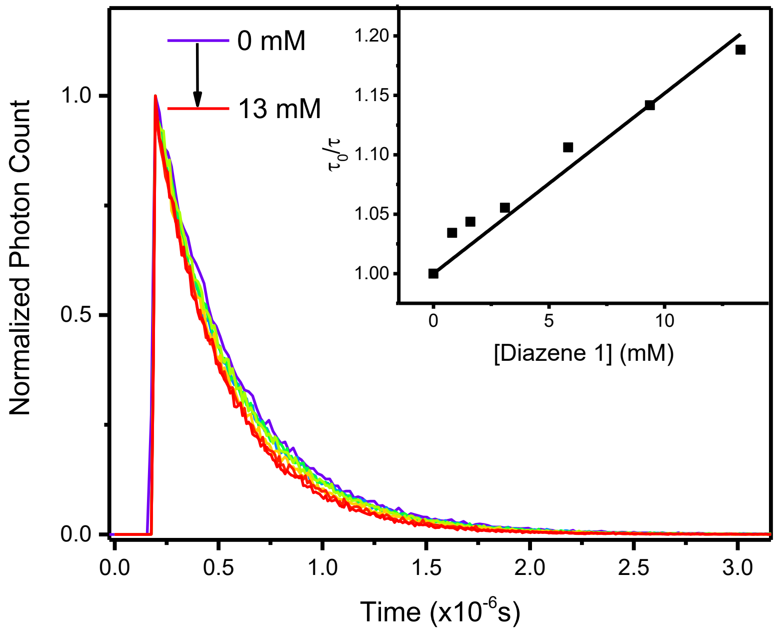

Figure S26: Normalized time-resolved emission measurements of $[Ir(dFCF_3ppy)_2((CF_3)_2bpy)]^+$ (**PC-6**) (λ_{det} = 600 nm, λ_{exc} = 450 nm) recorded in Ar-saturated acetonitrile in the presence of diazene **1** at different concentrations. Inset: Corresponding Stern-Volmer plot with linear fit from which the quenching rate constant was estimated. For discussion about photoproduct, see section **VII**. Due to competition between energy and electron transfer, the linearity of the Stern-Volmer plot is not ideal. The linear fit has been used to estimate the quenching rate constant.

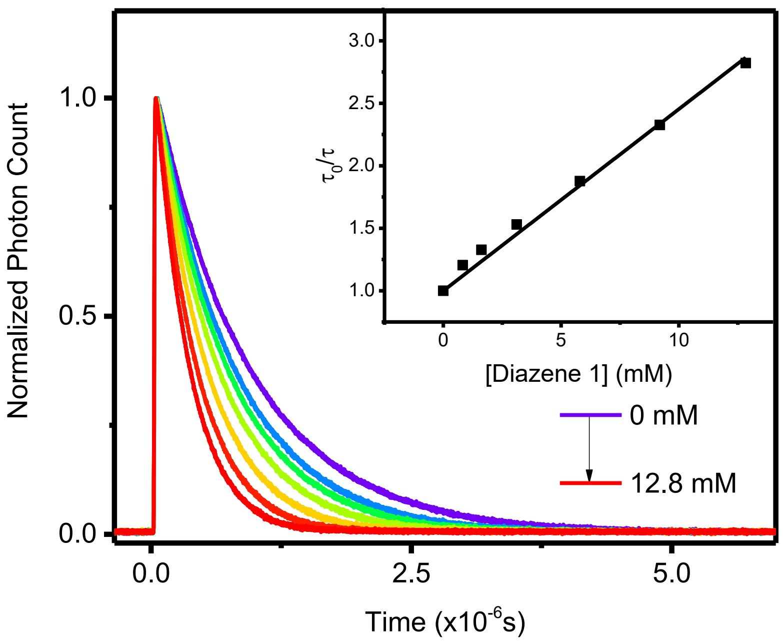

Figure S27: Normalized time-resolved emission measurements of $[Ir(Fppy)_2(bpy)]^+$ (**PC-7**) (λ_{det} = 560 nm, λ_{exc} = 410 nm) recorded in Ar-saturated acetonitrile in the presence of diazene **1** at different concentrations. Inset: Corresponding Stern-Volmer plot with linear fit from which the quenching rate constant was estimated.

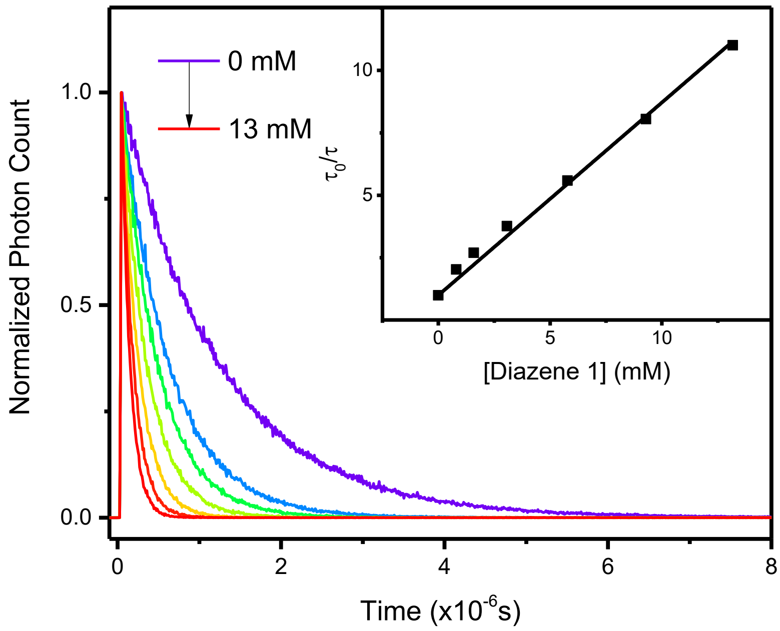

Figure S28: Normalized time-resolved emission measurements of $[Ir(ppy)_3]$ (**PC-8**) (λ_{det} = 520 nm, λ_{exc} = 450 nm) recorded in Ar-saturated acetonitrile in the presence of diazene **1** at different concentrations. Inset: Corresponding Stern-Volmer plot with linear fit from which the quenching rate constant was estimated.

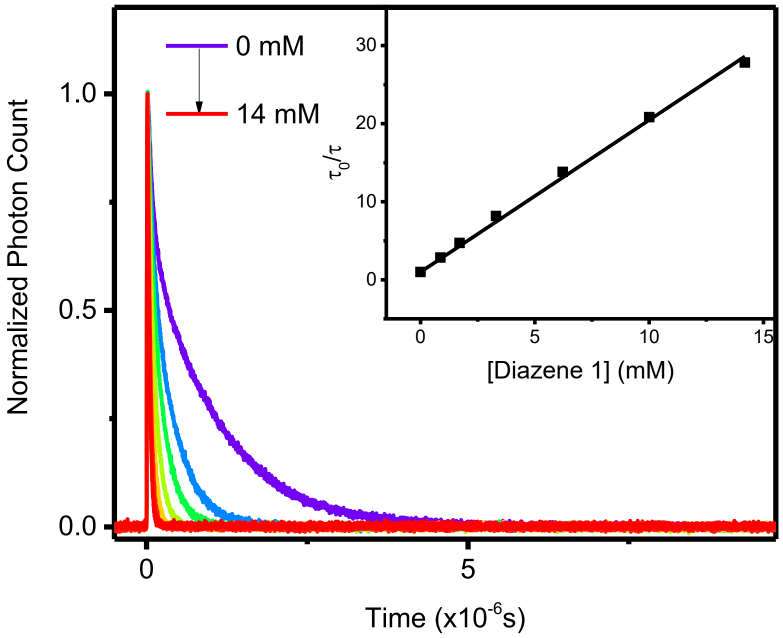

Figure S29: Normalized time-resolved emission measurements of [Ir(dFppy)$_3$] (**PC-9**) (λ_{det} = 490 nm, λ_{exc} = 410 nm) recorded in Ar-saturated acetonitrile in the presence of diazene **1** at different concentrations. Inset: Corresponding Stern-Volmer plot with linear fit from which the quenching rate constant was estimated.

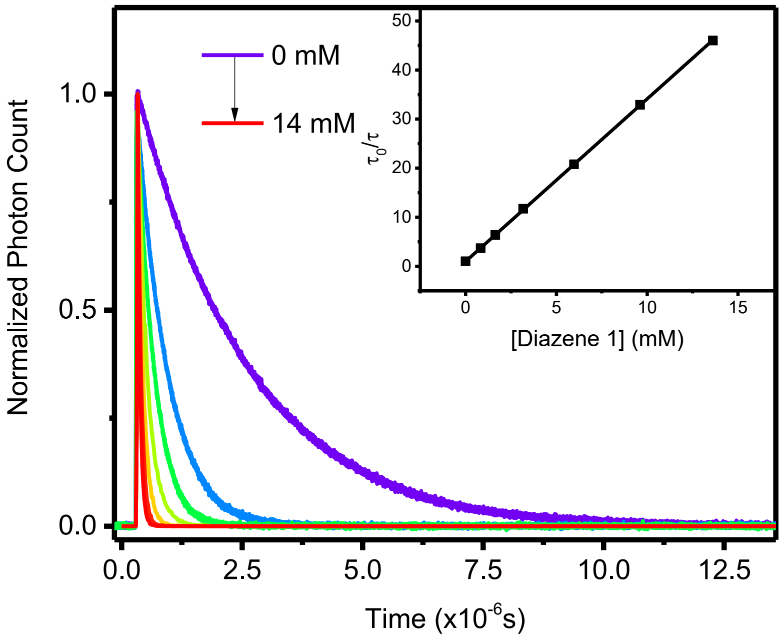

Figure S30: Normalized time-resolved emission measurements of [Ir(dFCF$_3$ppy)$_2$(dtb)]$^+$ (**PC-10**) (λ_{det} = 475 nm, λ_{exc} = 450 nm) recorded in Ar-saturated acetonitrile in the presence of diazene **1** at different concentrations. Inset: Corresponding Stern-Volmer plot with linear fit from which the quenching rate constant was estimated.

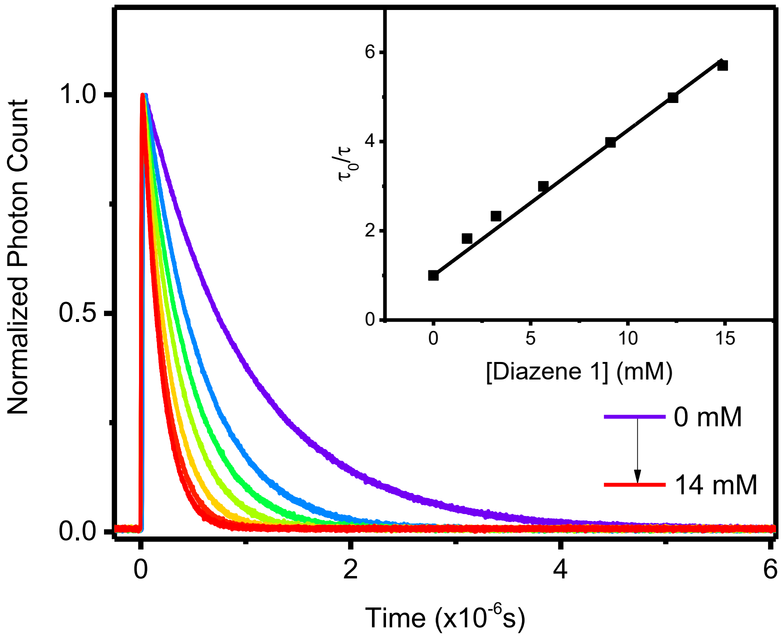

Figure S31: Normalized time-resolved emission measurements of $[Ir(dFppy)_2(bpy)]^+$ (**PC-11**) (λ_{det} = 530 nm, λ_{exc} = 410 nm) recorded in Ar-saturated acetonitrile in the presence of diazene **1** at different concentrations. Inset: Corresponding Stern-Volmer plot with linear fit from which the quenching rate constant was estimated.

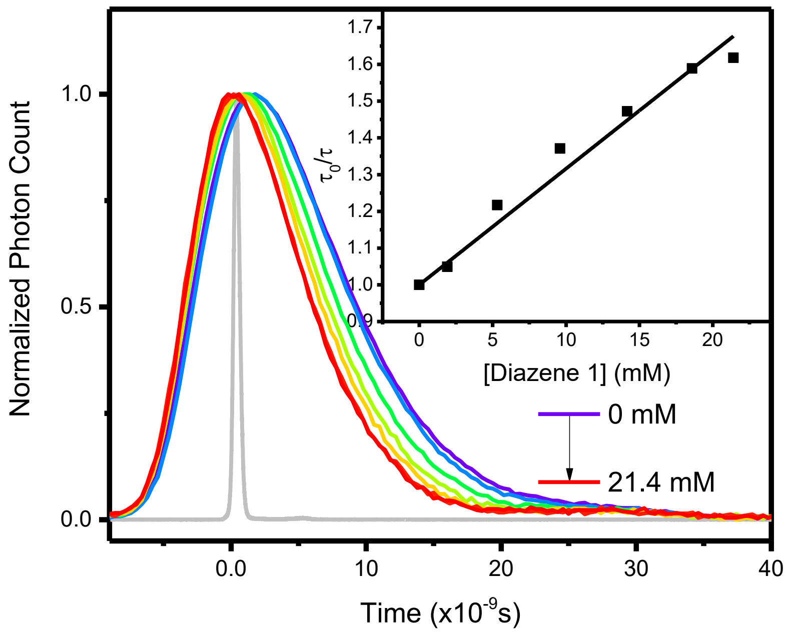

Figure S32: Normalized time-resolved emission measurements of 2,4,6-Triphenylpyrilium (**PC-12**) (λ_{det} = 470 nm, λ_{exc} = 450 nm) recorded in Ar-saturated acetonitrile in the presence of diazene **1** at different concentrations. The Instrument Response Function (IRF) is shown in clear grey. Inset: Corresponding Stern-Volmer plot with linear fit from which the quenching rate constant was estimated.

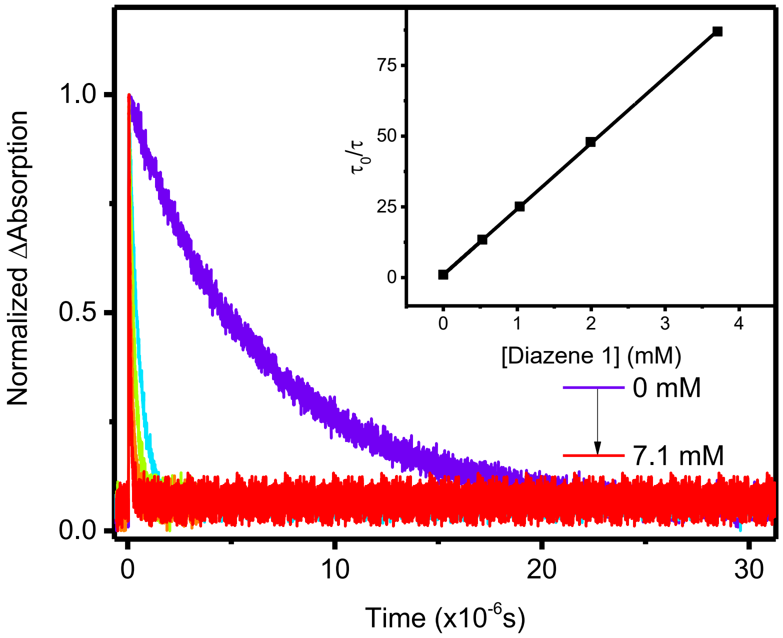

Figure S33: Normalized single wavelength absorption changes of Benzophenone (**PC-13**) (λ_{det} = 530 nm, λ_{exc} = 355 nm) recorded in Ar-saturated acetonitrile in the presence of diazene **1** at different concentrations. Inset: Corresponding Stern-Volmer plot with linear fit from which the quenching rate constant was estimated.

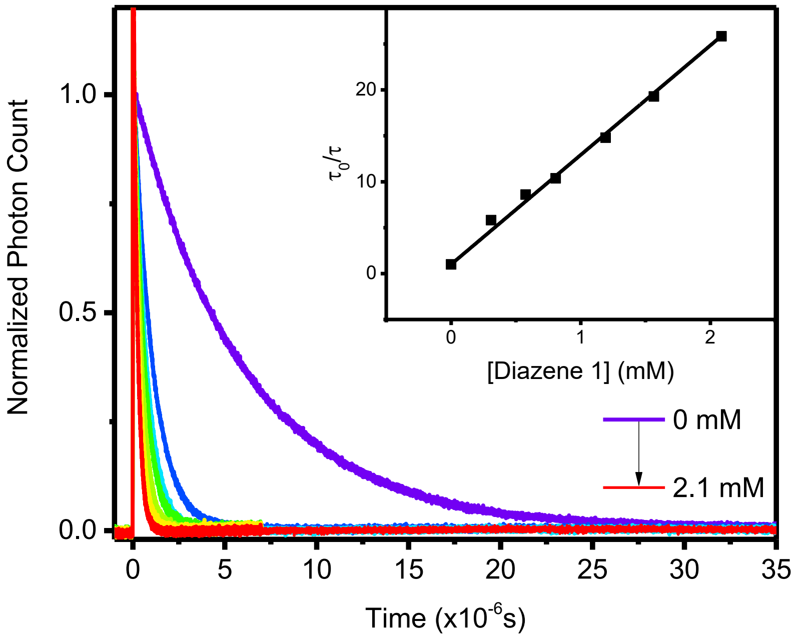

Figure S34: Normalized time-resolved emission measurements of [Ir(CF_3pmb)$_3$] (**PC-14**) (λ_{det} = 420 nm, λ_{exc} = 355 nm) recorded in Ar-saturated acetonitrile in the presence of diazene **1** at different concentrations. Inset: Corresponding Stern-Volmer plot with linear fit from which the quenching rate constant was estimated.

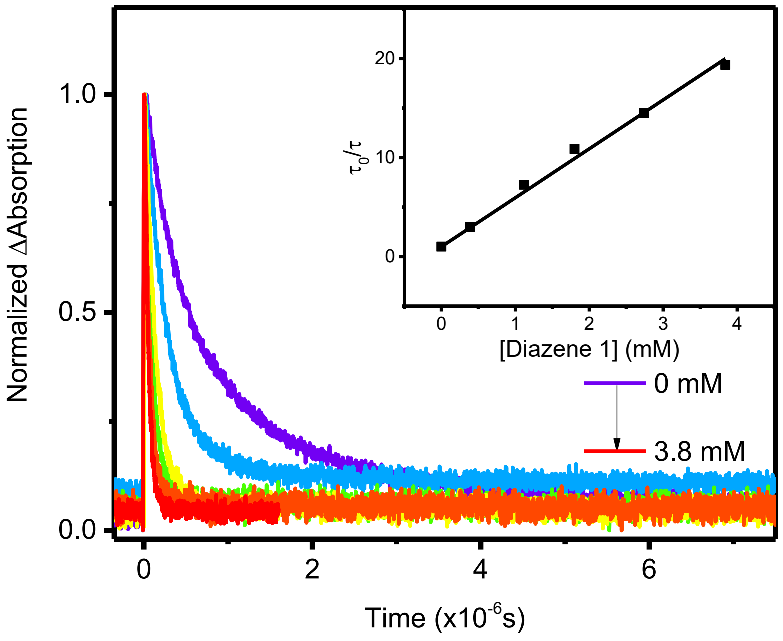

Figure S35: Normalized single wavelength absorption changes of Acetophenone (**PC-15**) (λ_{det} = 340 nm, λ_{exc} = 355 nm) recorded in Ar-saturated acetonitrile in the presence of diazene **1** at different concentrations. Inset: Corresponding Stern-Volmer plot with linear fit from which the quenching rate constant was estimated.

<u>Quenching measurement with diazene **2**</u>

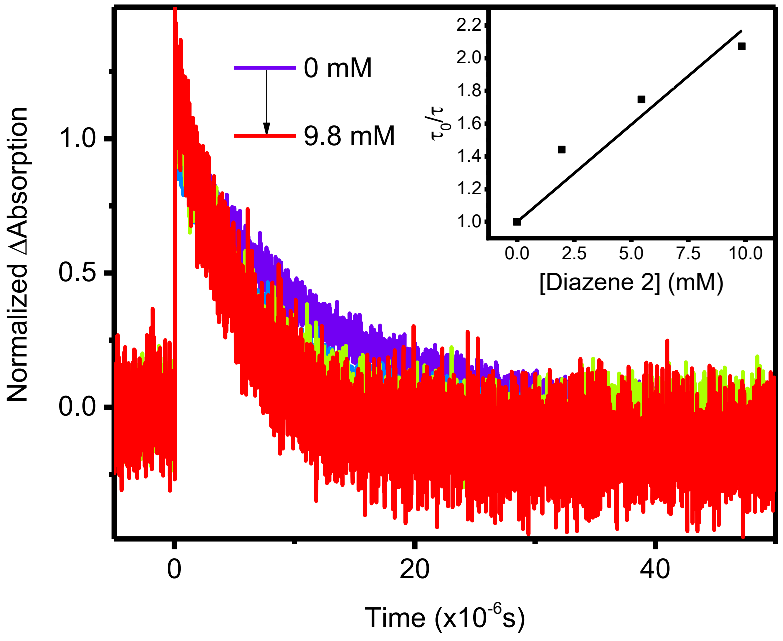

Figure S36: Normalized single wavelength absorption changes of Acr-Me (**PC-1**) (λ_{det} = 495 nm, λ_{exc} = 450 nm) in Ar-saturated acetonitrile in the presence of diazene **2** at different concentrations. Inset: Corresponding Stern-Volmer plot with linear fit from which the quenching rate constant was estimated. For discussion about photoproduct, see section **VII**. Due to presence of photoproduct, the linearity of the Stern-Volmer plot is not ideal. The linear fit has been used to estimate the quenching rate constant.

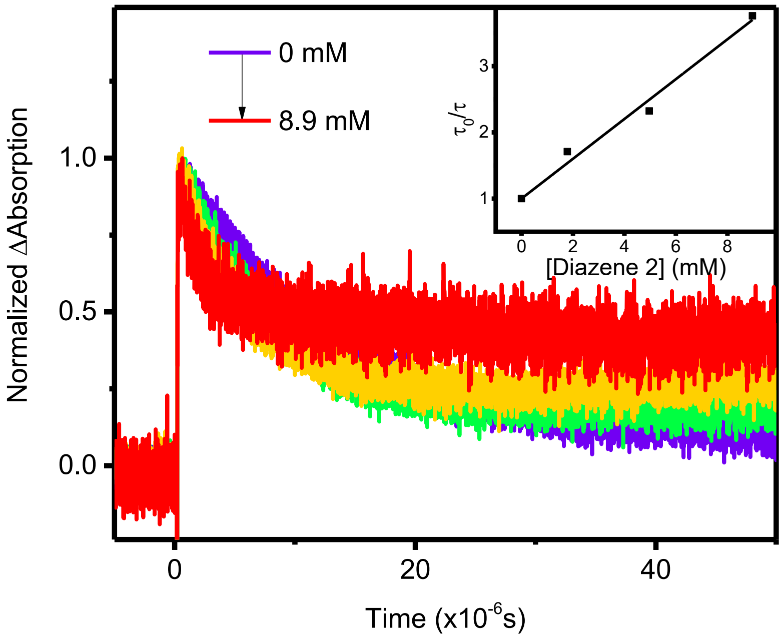

Figure S37: Normalized single wavelength absorption changes of Acr-Ph (**PC-2**) (λ_{det} = 500 nm, λ_{exc} = 450 nm) in Ar-saturated acetonitrile in the presence of diazene **2** at different concentrations. Inset: Corresponding Stern-Volmer plot with linear fit from which the quenching rate constant was estimated. For discussion about photoproduct, see section **VII**. Due to presence of photoproduct, the linearity of the Stern-Volmer plot is not ideal. The linear fit has been used to estimate the quenching rate constant.

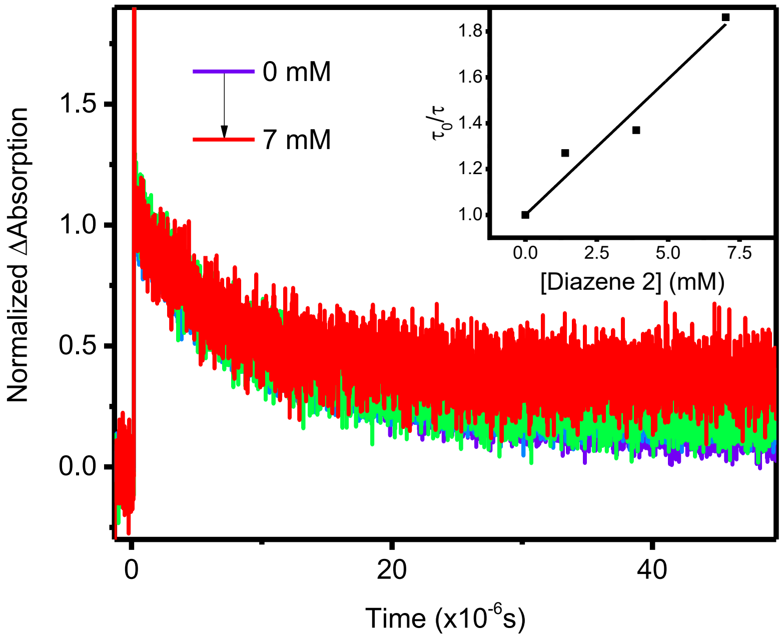

Figure S38: Normalized single wavelength absorption changes of Acr-^{t}Bu (**PC-3**) (λ_{det} = 500 nm, λ_{exc} = 450 nm) in Ar-saturated acetonitrile in the presence of diazene **2** at different concentrations. Inset: Corresponding Stern-Volmer plot with linear fit from which the quenching rate constant was estimated. For discussion about photoproduct, see section **VII**. Due to presence of photoproduct, the linearity of the Stern-Volmer plot is not ideal. The linear fit has been used to estimate the quenching rate constant.

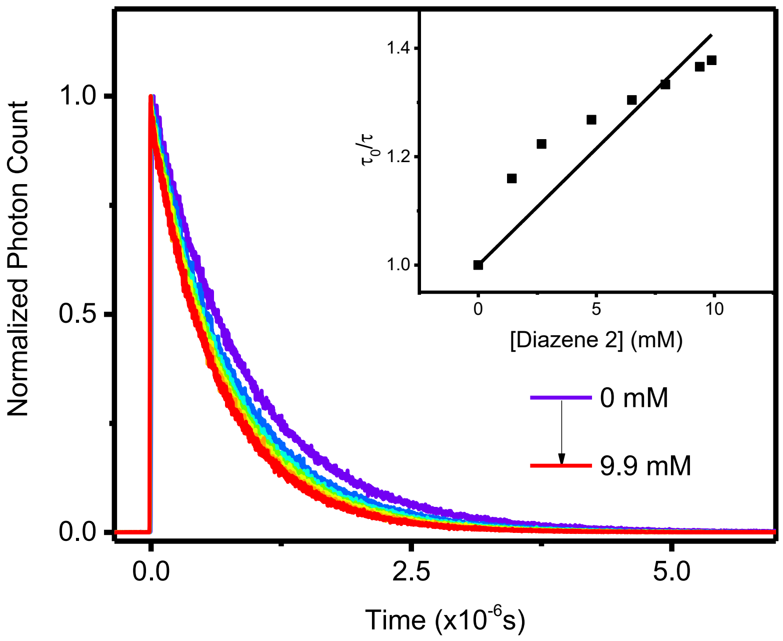

Figure S39: Normalized time-resolved emission measurements of $[Ru(bpy)_3]^{2+}$ (**PC-4**) (λ_{det} = 600 nm, λ_{exc} = 410 nm) recorded in Ar-saturated acetonitrile in the presence of diazene **2** at different concentrations. Inset: Corresponding Stern-Volmer plot with linear fit from which the quenching rate constant was estimated. Due to low quenching, the linearity of the Stern-Volmer plot is not ideal. The linear fit has been used to estimate the quenching rate constant.

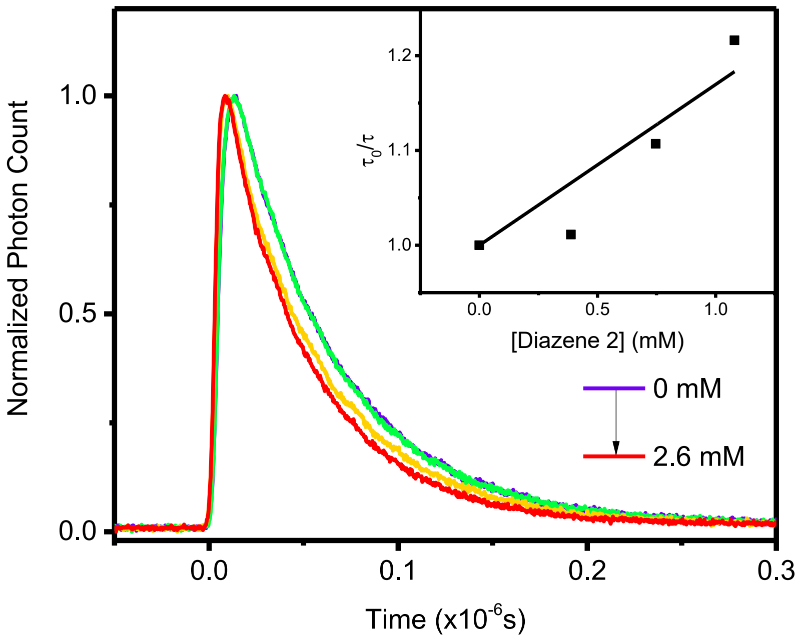

Figure S40: Normalized time-resolved emission measurements of $[Ru(TAP)_3]^{2+}$ (**PC-5**) (λ_{det} = 600 nm, λ_{exc} = 450 nm) recorded in Ar-saturated acetonitrile in the presence of diazene **2** at different concentrations. Inset: Corresponding Stern-Volmer plot with linear fit from which the quenching rate constant was estimated. Due to low quenching, the linearity of the Stern-Volmer plot is not ideal. The linear fit has been used to estimate the quenching rate constant.

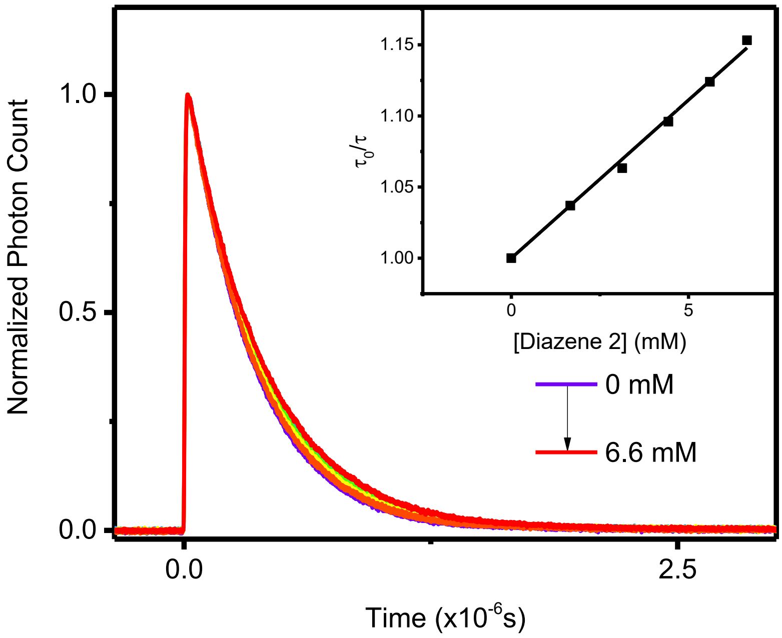

Figure S41: Normalized time-resolved emission measurements of $[Ir(dFCF_3ppy)_2((CF_3)_2bpy)]^+$ (**PC-6**) (λ_{det} = 600 nm, λ_{exc} = 450 nm) recorded in Ar-saturated acetonitrile in the presence of diazene **2** at different concentrations. Inset: Corresponding Stern-Volmer plot with linear fit from which the quenching rate constant was estimated. For discussion about photoproduct, see section **VII**.

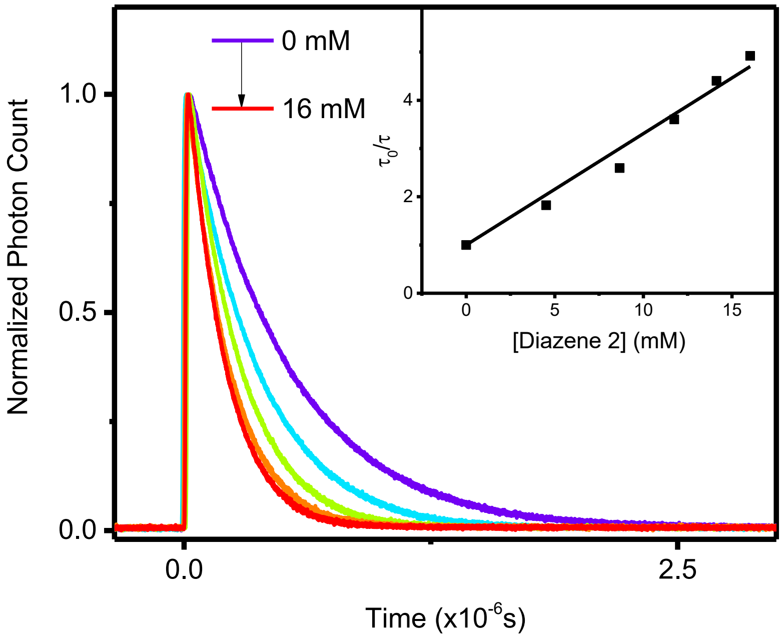

Figure S42: Normalized time-resolved emission measurements of $[Ir(Fppy)_2(bpy)]^+$ (**PC-7**) (λ_{det} = 560 nm, λ_{exc} = 410 nm) recorded in Ar-saturated acetonitrile in the presence of diazene **2** at different concentrations. Inset: Corresponding Stern-Volmer plot with linear fit from which the quenching rate constant was estimated.

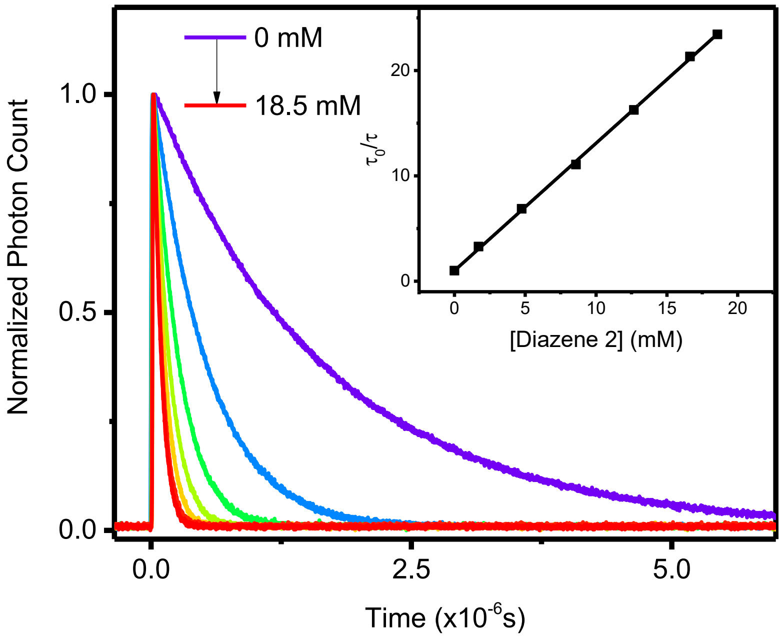

Figure S43: Normalized time-resolved emission measurements of [Ir(ppy)$_3$] (**PC-8**) (λ_{det} = 520 nm, λ_{exc} = 450 nm) recorded in Ar-saturated acetonitrile in the presence of diazene **2** at different concentrations. Inset: Corresponding Stern-Volmer plot with linear fit from which the quenching rate constant was estimated.

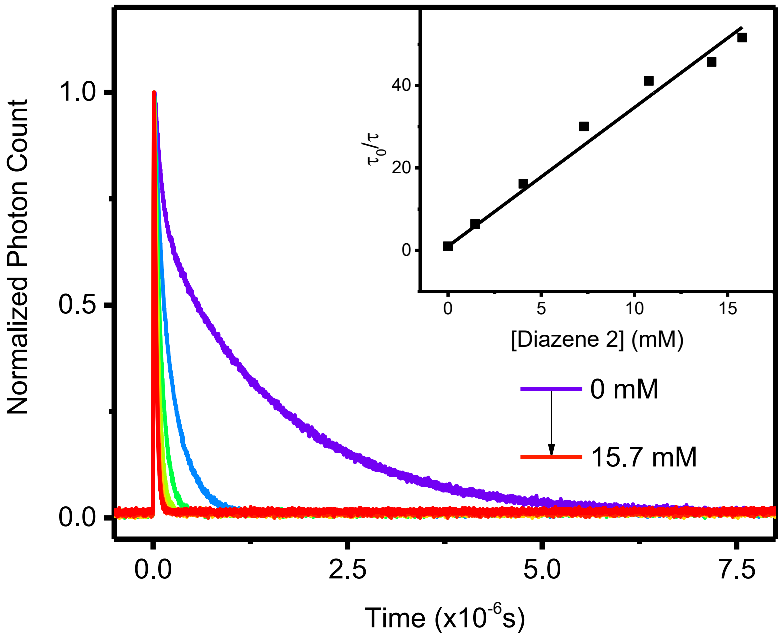

Figure S44: Normalized time-resolved emission measurements of [Ir(dFppy)$_3$] (**PC-9**) (λ_{det} = 590 nm, λ_{exc} = 410 nm) recorded in Ar-saturated acetonitrile in the presence of diazene **2** at different concentrations. Inset: Corresponding Stern-Volmer plot with linear fit from which the quenching rate constant was estimated.

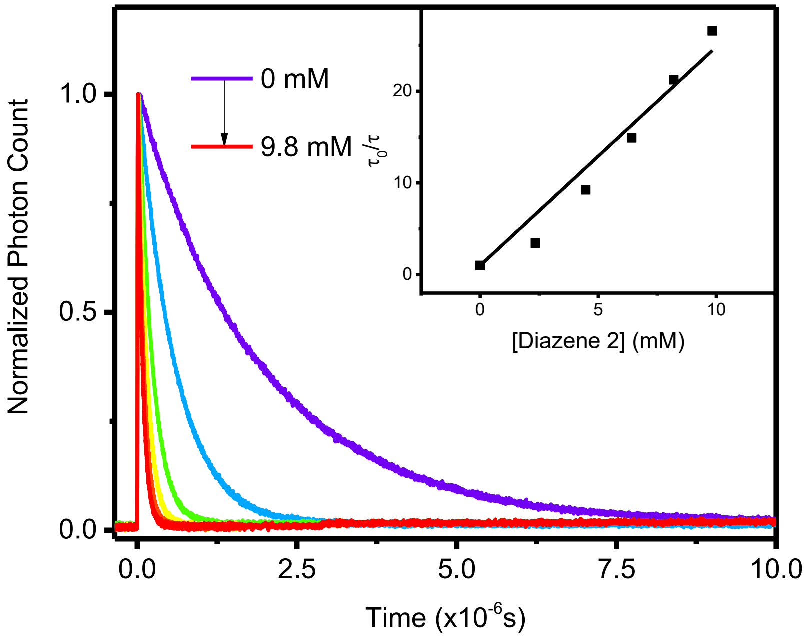

Figure S45: Normalized time-resolved emission measurements of [Ir(dFCF$_3$ppy)$_2$(dtb)]$^+$ (**PC-10**) (λ_{det} = 475 nm, λ_{exc} = 450 nm) recorded in Ar-saturated acetonitrile in the presence of diazene **2** at different concentrations. Inset: Corresponding Stern-Volmer plot with linear fit from which the quenching rate constant was estimated.

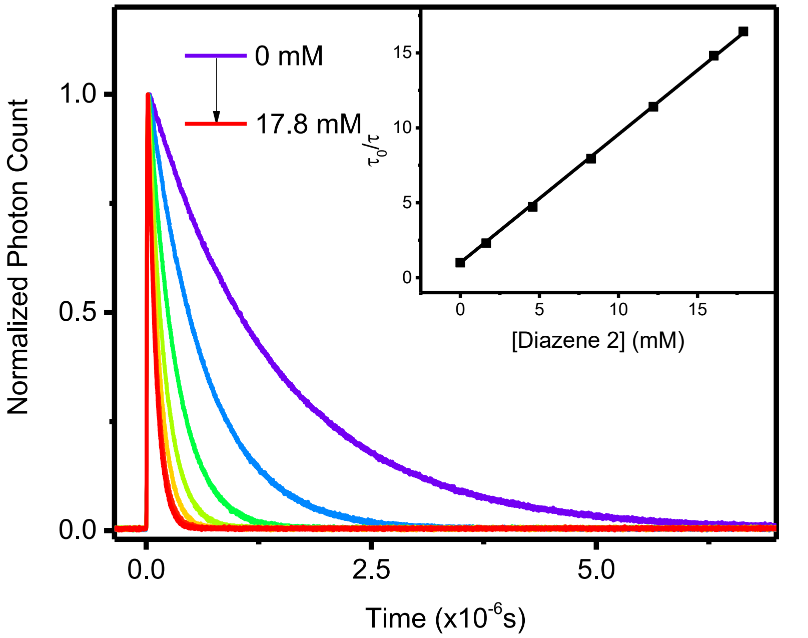

Figure S46: Normalized time-resolved emission measurements of [Ir(dFppy)$_2$(bpy)]$^+$ (**PC-11**) (λ_{det} = 530 nm, λ_{exc} = 410 nm) recorded in Ar-saturated acetonitrile in the presence of diazene **2** at different concentrations. Inset: Corresponding Stern-Volmer plot with linear fit from which the quenching rate constant was estimated.

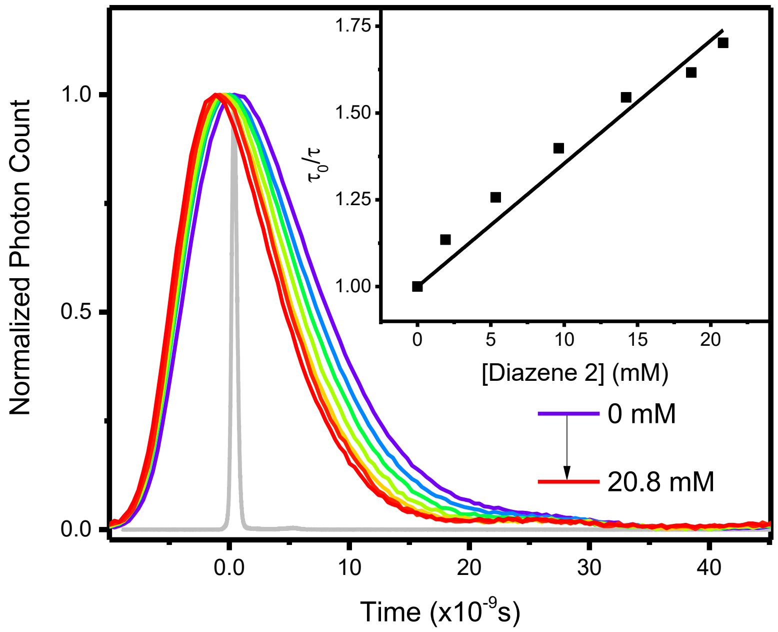

Figure S47: Normalized time-resolved emission measurements of 2,4,6-Triphenylpyrilium (**PC-12**) (λ_{det} = 470 nm, λ_{exc} = 450 nm) recorded in Ar-saturated acetonitrile in the presence of diazene **2** at different concentrations. The Instrument Response Function (IRF) is shown in clear grey. Inset: Corresponding Stern-Volmer plot with linear fit from which the quenching rate constant was estimated.

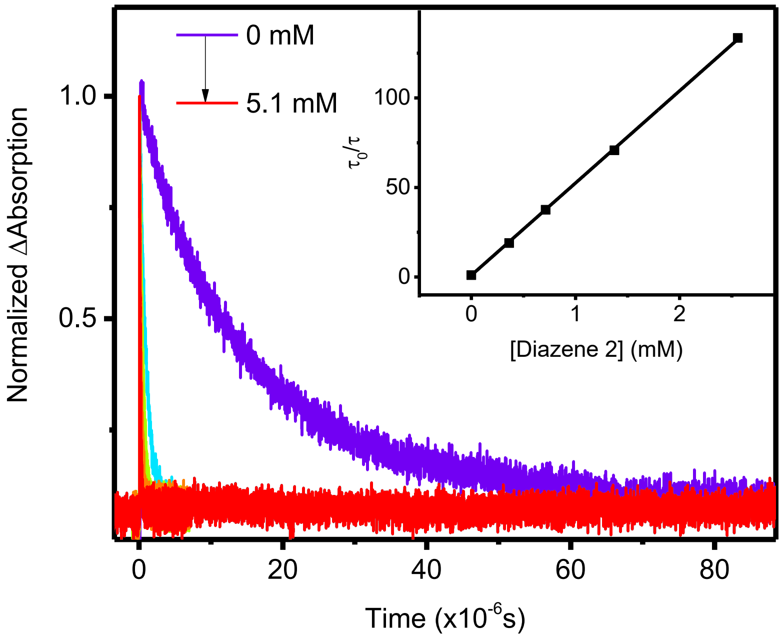

Figure S48: Normalized single wavelength absorption changes of Benzophenone (**PC-13**) (λ_{det} = 530 nm, λ_{exc} = 355 nm) in Ar-saturated acetonitrile in the presence of diazene **2** at different concentrations. Inset: Corresponding Stern-Volmer plot with linear fit from which the quenching rate constant was estimated.

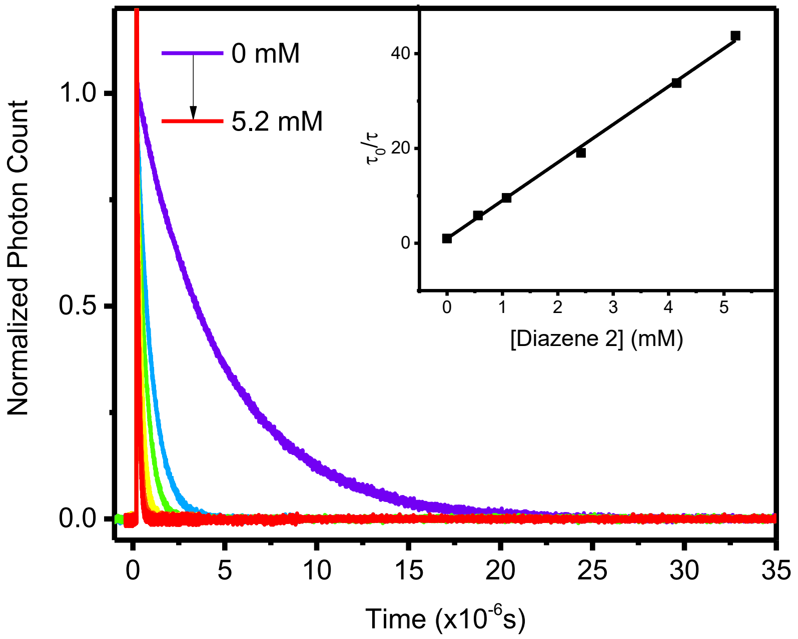

Figure S49: Normalized time-resolved emission measurements of [Ir(CF_3pmb)$_3$] (**PC-14**) (λ_{det} = 420 nm, λ_{exc} = 355 nm) recorded in Ar-saturated acetonitrile in the presence of diazene **2** at different concentrations. Inset: Corresponding Stern-Volmer plot with linear fit from which the quenching rate constant was estimated.

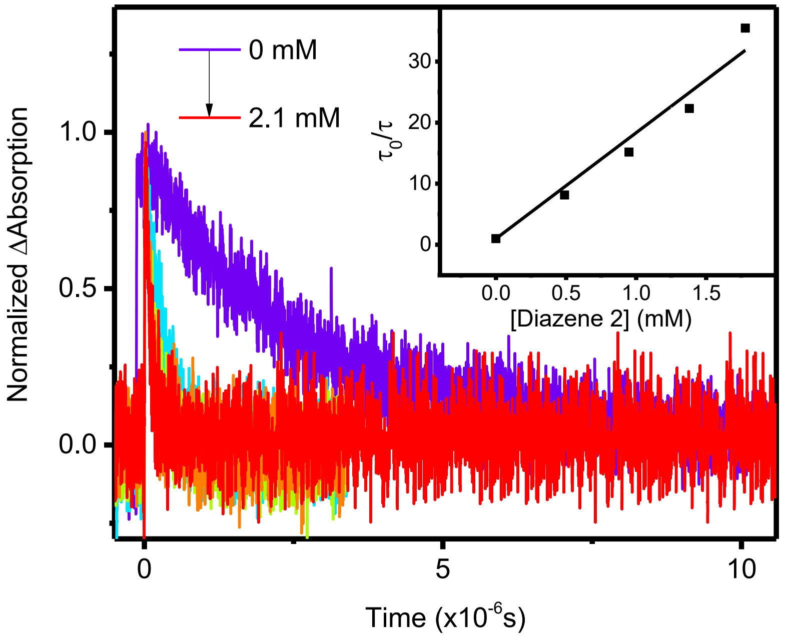

Figure S50: Normalized single wavelength absorption changes of Acetophenone (**PC-15**) (λ_{det} = 340 nm, λ_{exc} = 355 nm) in Ar-saturated acetonitrile in the presence of diazene **2** at different concentrations. Inset: Corresponding Stern-Volmer plot with linear fit from which the quenching rate constant was estimated.

VI. Nanosecond Transient Absorption Spectroscopy

The nanosecond transient absorption spectra shown below have been taken after the Stern-Volmer quenching between the PC and the diazene **1** or **2** (see section **VI**). Triethylamine (electron donor) and 4-Methoxybenzenediazonium tetrafluoroborate (electron acceptor) have been used in separate experiments to identify the spectral signature of the monoreduced or monooxidized PC, respectively. These quenchers have been chosen because of their electrochemical potentials and the absence of photoproducts absorbing in the investigated optical window, thereby allowing to solely monitor the PC's photoproduct.

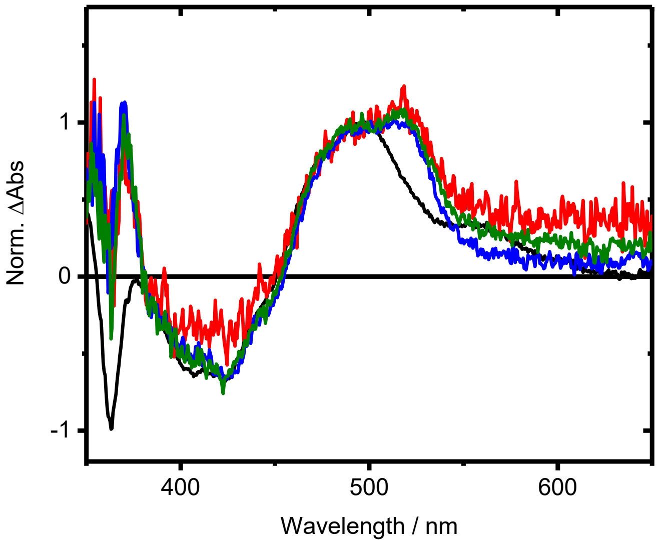

Figure S51: Nanosecond transient absorption spectroscopy of Acr-Me (**PC-1**) (black) recorded in the presence of 30 mM of triethylamine (red), 12 mM of diazene **1** (blue) and 21 mM of diazene **2** (green). Experiments were carried out in argon purged acetonitrile at room temperature following pulsed 450 nm light excitation. Except for the black curve, all spectra are recorded after the excited-state decay. The presence of monoreduced Acr-Me (**PC-1**) was confirmed by the absorption changes presenting a maximum at 530 nm. See **Figures S21** and **S36**: for the corresponding Stern-Volmer Analyses.

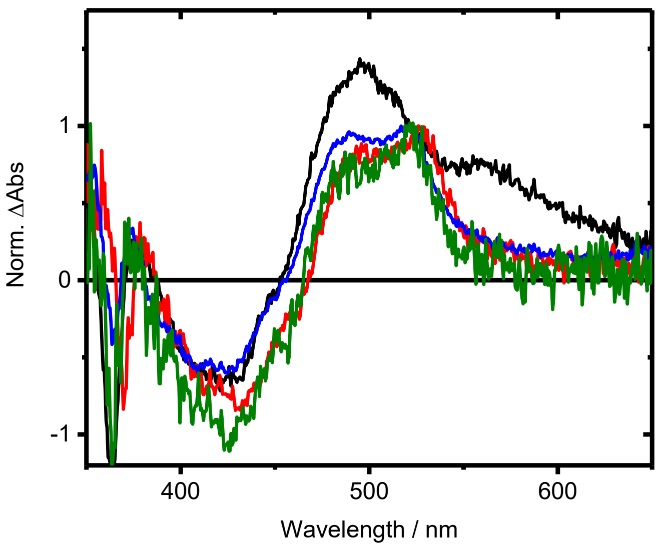

Figure S52: Nanosecond transient absorption spectroscopy of Acr-Ph (**PC-2**) (black) recorded in the presence of 26 mM of triethylamine (red), 20 mM of diazene **1** (blue) and 8.9 mM of diazene **2** (green). Experiments were carried out in argon purged acetonitrile at room temperature following pulsed 450 nm light excitation. Except for the black curve, all spectra are recorded after the excited-state decay. The presence of monoreduced Acr-Ph (**PC-2**) was confirmed by the absorption changes presenting a loss of signal after 550 nm. See **Figures S22** and **S37**: for the corresponding Stern-Volmer Analyses.

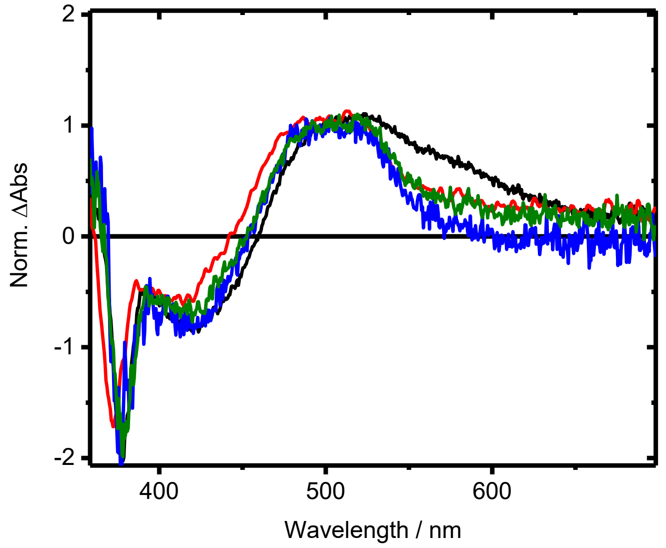

Figure S53: Nanosecond transient absorption spectroscopy of Acr-^{t}Bu (**PC-3**) (black) recorded in the presence of 35 mM of triethylamine (red), 21 mM of diazene **1** (blue) and 15 mM of diazene **2** (green). Experiments were carried out in argon purged acetonitrile at room temperature following pulsed 450 nm light excitation. Except for the black curve, all spectra are recorded after the excited-state. The presence of monoreduced Acr-^{t}Bu (**PC-3**) was confirmed by the absorption changes presenting a loss of signal between 550 and 610 nm. See **Figures S23** and **S38**: for the corresponding Stern-Volmer Analyses.

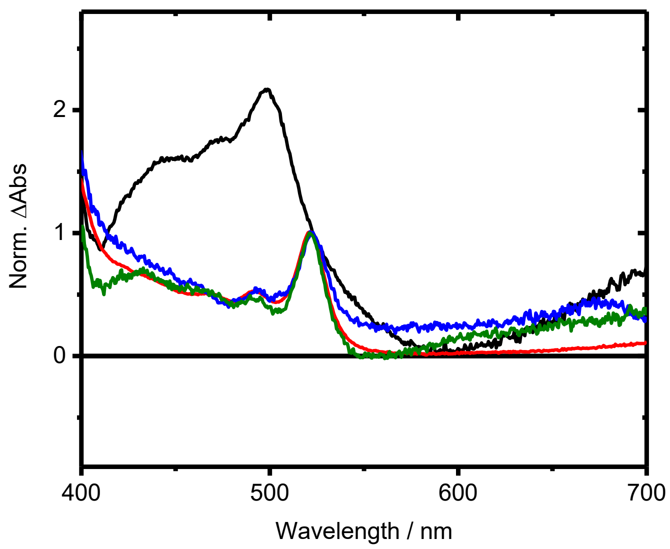

Figure S54: Nanosecond transient absorption spectroscopy of $[Ir(dFCF_3ppy)_2((CF_3)_2bpy)]^+$ (**PC-6**) (black) recorded in the presence of 10 mM of triethylamine (red), 13 mM of diazene **1** (blue) and 6.6 mM of diazene **2** (green). Experiments were carried out in argon purged acetonitrile at room temperature following pulsed 450 nm light excitation. Except for the black curve, all spectra are recorded after the excited-state. The presence of monoreduced $[Ir(dFCF_3ppy)_2((CF_3)_2bpy)]^+$ (**PC-6**) was confirmed by the absorption changes presenting a new signal at 520 nm. See **Figures S26** and **S41**: for the corresponding Stern-Volmer Analyses.

VII. Rehm-Weller Analyses

The quantitative relationship between the Gibbs free energy change of an electron-transfer event (ΔG_{et}) and the corresponding quenching rate constant (k_q) was first established by Rehm and Weller.[16] Their empirical treatment describes how an excited PC interacts with a quencher (Q), acting either as an electron donor or acceptor, and provides a practical framework for interpreting excited-state quenching kinetics. The free-energy change associated with photoinduced electron transfer is given in **Equation S1**.

$$\Delta G_{et} = E_{\frac{1}{2}}(Q) - E_{\frac{1}{2}}(PC^{*}) \qquad \textbf{Eq.S1}$$

Building on classical electron-transfer theory Rehm and Weller expressed the rate constant of electron transfer (k_{et}) through **Equation S2**, which incorporates both the diffusion-controlled limit (k_{diff}) and the dissociation rate constant of the encounter complex (k_{-diff}). The activation free energy ($\Delta G_{et}^{\#}$) is defined in **Equation S3** and includes a reorganization term (λ), capturing the energetic cost of nuclear rearrangement during charge transfer. ,[17-18]

$$k_{\text{et}} = \frac{k_{\text{diff}}}{1 + \frac{k_{-\text{diff}}}{Z}\left[\exp\left(\frac{\Delta G_{\text{et}}}{k_b T}\right) + \exp\left(\frac{\Delta G_{\text{et}}^{\#}}{k_b T}\right)\right]} \qquad \textbf{Eq.S2}$$

$$\Delta G_{\text{et}}^{\#} = \frac{\Delta G_{\text{et}}}{2} + \left(\left(\frac{\Delta G_{\text{et}}}{2}\right)^2 + \left(\frac{\lambda}{4}\right)^2\right)^{\frac{1}{2}} \qquad \textbf{Eq.S3}$$

In acetonitrile at room temperature: $$k_{et} = \frac{2\,x10^{10}\,M^{-1}s^{-1}}{1+0.25\left[\exp\left(\frac{\Delta G_{et}}{0.0252}\right) + \exp\left(\frac{\Delta G_{et}^{\#}}{0.0252}\right)\right]} \qquad \textbf{Eq.S4}$$

In **Equation S4**, k_{diff} denotes the bimolecular diffusion rate constant, while k_{-diff} describes the dissociation of the transient encounter complex. The pre-exponential factor of 0.25, which corresponds to k_{-diff}/Z, was extracted by Rehm and Weller based on the solvent's collision frequency (Z) and the dissociation rate constant. For acetonitrile, the diffusion-controlled limit of 2 x10^{10} $M^{-1}s^{-1}$ sets the upper boundary for observable k_{et} values.[19] At room temperature, the Boltzmann constant k_b expressed in eV/K multiplied by the temperature T can be approximated to 0.0252 eV. Importantly, the empirical treatment captures the transition to diffusion control but does not account for more complex kinetic pathways beyond this regime e.g. when

recombination in the solvent cage is significant (low cage escape), k_q no longer follows the Rehm-Weller relationship.[20]

An analogous strategy can be applied to excited-state quenching operating via energy transfer. In this context, the energy gap between donor (EnD) and acceptor (EnA) determines the rate of energy transfer (k_{EnT}). A simplified expression was introduced by Sandros, based on a Boltzmann distribution of donor-acceptor energy transfer events (**Equation S5**), where $\Delta G_{EnT} = E_{EnD} - E_{EnA}$.[21]

$$k_{\mathrm{EnT}} = \frac{k_{\mathrm{diff}}}{1 + \exp\left(\frac{-\Delta G_{\mathrm{EnT}}}{k_b T}\right)} \qquad \boldsymbol{Eq.S5}$$

As with electron transfer, the rate approaches the diffusion-controlled limit for large driving forces. However, deviations from this behavior were observed in some systems, prompting Balzani and coworkers to introduce a more general "non-vertical" model, **Equation 3**,[22] which closely resembles the Weller-Rehm approach for electron transfer and is therefore here also referred to as Weller-Rehm-type analysis(reproduced below for clarity).

$$k_{\mathrm{EnT}} = \frac{k_{\mathrm{diff}}}{1 + \exp\left(\frac{\Delta G_{\mathrm{EnT}}}{k_b T}\right) + \frac{k_{-\mathrm{diff}}}{Z}\exp\left(\frac{\Delta G^{\#}_{\mathrm{EnT}}}{k_b T}\right)} \qquad \boldsymbol{Eq.3}$$

$$\Delta G^{\#}_{\mathrm{EnT}} = \frac{\Delta G_{EnT}}{2} + \left(\left(\frac{\Delta G_{EnT}}{2}\right)^2 + \left(\frac{\lambda}{4}\right)^2\right)^{\frac{1}{2}} \qquad \boldsymbol{Eq.4}$$

This formulation extends Sandros' equation by including an activation barrier ($\Delta G_{EnT}^{\ddagger}$), which becomes critical for excited states that are significantly distorted relative to the ground state. The expression of this term (**Equation 4**) is similar to the activation free energy ($\Delta G_{et}^{\#}$). For weakly distorted excited states, the additional term is negligible and the simpler Sandros expression is sufficient. The formal similarity between the electron-transfer (**Equations S2-S3**) and energy-transfer (**Equations 3-4**) is not surprising, as both are based on analogous assumptions about the nature of excited-state quenching.

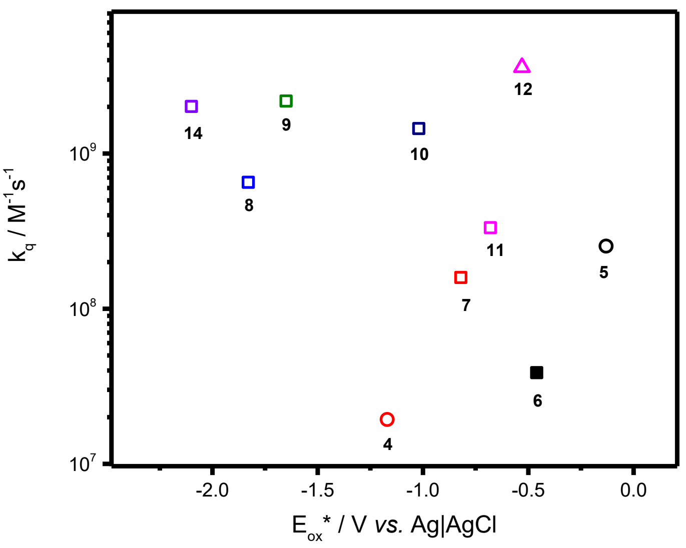

Figure S55: Rehm-Weller plot gathering the 10 PCs quenching rate constants for the quenching with diazene **1** as a function of excited-state oxidation potential. Experiments were carried out in argon purged acetonitrile at room temperature. Ir(III) PCs are represented by a square, Ru(II) PCs by a circle and organic PCs by a triangle. Symbols are filled when photoproducts corresponding to excited-state electron transfer were observed and empty when no photoproducts were detected. Numbers refer to PCs in table 1. The PCs that do not show oxidative wave between 0 and +2.5 V vs. Ag/AgCl in cyclic voltammetry are not included in the plot.

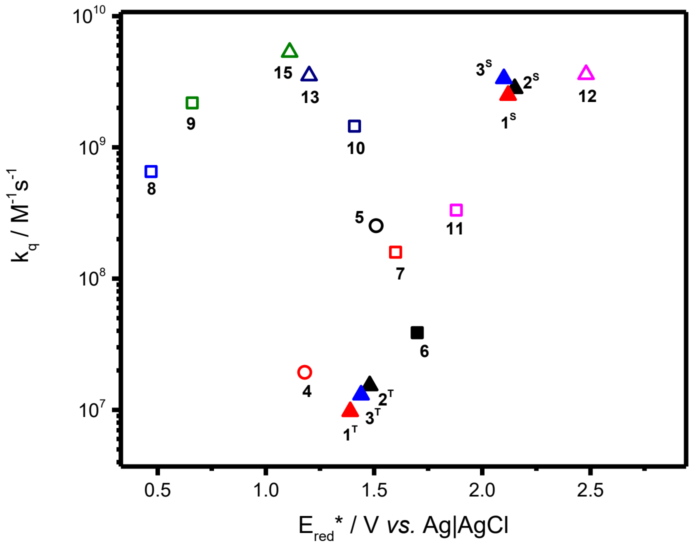

Figure S56: Rehm-Weller plot gathering the 14 PCs quenching rate constants for the quenching with diazene **1** as a function of excited-state reduction potential. Experiments were carried out in argon purged acetonitrile at room temperature. Ir(III) PCs are represented by a square, Ru(II) PCs by a circle and organic PCs by a triangle. Symbols are filled when photoproducts corresponding to excited-state electron transfer were observed and empty when no photoproducts were detected. Numbers refer to PCs in table 1. **PC-14** that do not show reductive wave between 0 and -2.5 V vs. Ag/AgCl in cyclic voltammetry are not included in the plot.

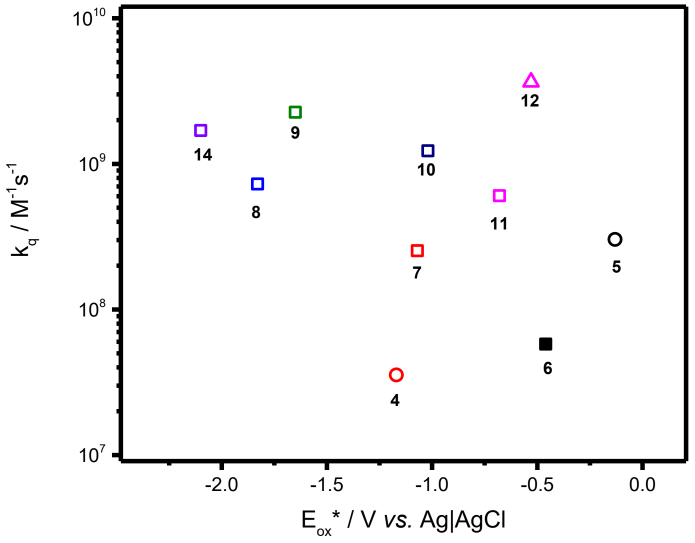

Figure S57: Rehm-Weller plot gathering the 10 PCs quenching rate constants for the quenching with diazene **2** as a function of excited state oxidation potential. Experiments were carried out in argon purged acetonitrile at room temperature. Ir(III) PCs are represented by a square, Ru(II) PCs by a circle and organic PCs by a triangle. Symbols are filled when photoproducts corresponding to excited-state electron transfer were observed and empty when no photoproducts were detected. Numbers refer to PCs in table 1. The PCs that do not show oxidative wave between 0 and +2.5 V vs. Ag/AgCl in cyclic voltammetry are not included in the plot.

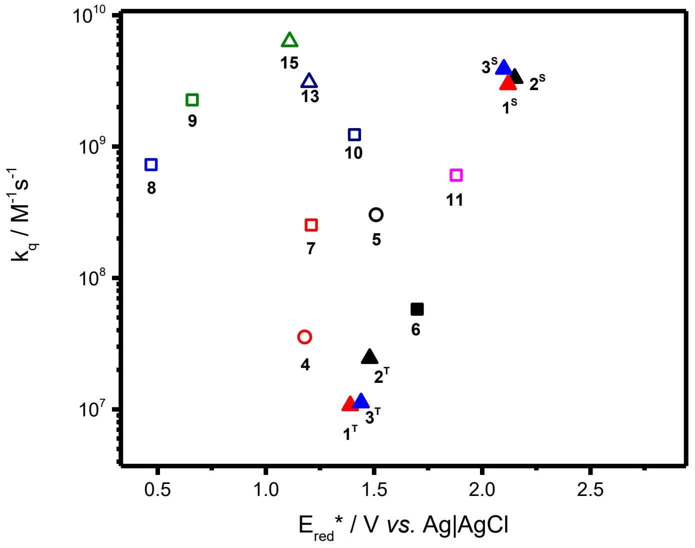

Figure S58: Rehm-Weller plot gathering the 14 PCs quenching rate constants for the quenching with diazene **2** as a function of excited state reduction potential. Experiments were carried out in argon purged acetonitrile at room temperature. Ir(III) PCs are represented by a square, Ru(II) PCs by a circle and organic PCs by a triangle. Symbols are filled when photoproducts corresponding to excited-state electron transfer were observed and empty when no photoproducts were detected. Numbers refer to PCs in table 1. The PCs that do not show reductive wave between 0 and -2.5 V vs. Ag/AgCl in cyclic voltammetry are not included in the plot.

VIII. Mediator-enhanced triplet energy transfer strategy

To quantify the maximal amount of triplet anthracene directly produced by the iridium photosensitizer, we determined (i) the molar absorption coefficient change (Δε) associated with triplet anthracene (Anth) and (ii) the concentration of excited iridium [Ir*] generated under our experimental conditions. To do so, $[Ru(bpy)_3]^{2+}$ was used as an actinometer due to its excited state displays a well-characterized by a ground-state bleach at 455 nm (Δε value of –10100 $M^{-1}cm^{-1}$).[23-24]

A solution of $[Ru(bpy)_3]^{2+}$ without anthracene and a solution of $[Ru(bpy)_3]^{2+}$ containing 0.1 mM of anthracene are prepared with the same absorption at the excitation wavelength (Abs = 0.3), ensuring matched photon absorption per pulse.

i. Determination of $\Delta\varepsilon_{Anthr}$.

Starting from the Beer-Lambert law (**Equations S6** and **S7**), the differential signal of an excited species can be expressed as:

$$A = \varepsilon.c.l \qquad \textbf{Eq.S6}$$

$$\Delta A = \Delta\varepsilon.c.l \qquad \textbf{Eq.S7}$$

Anthracene efficiently quenches the ^{3}MLCT state of $[Ru(bpy)_3]^{2+}$. The quenching efficiency is quantified from the ratio of excited-state lifetimes of the reference sample (τ_0) and the sample with anthracene (τ).

$$\phi_{Quenching} = 1 - \frac{\tau}{\tau_0} = 1 - \frac{115}{2250} = 0.95 \qquad \textbf{Eq.S8}$$

Using this quenching efficiency, $\Delta\varepsilon_{Anth}$ was obtained by comparing the ΔA of ^{3}Anth with the ground-state bleach of the actinometer:

$$\Delta\varepsilon_{Anth} = \frac{\Delta A_{Anth}}{\Delta A_{Acti}}.\Delta\varepsilon_{Acti}.\frac{1}{\phi_{Quenching}} \qquad \textbf{Eq.S9}$$

$$\Delta\varepsilon_{Acti} = \Delta\varepsilon_{Rubipy@455} = -10100\ M^{-1}cm^{-1}$$

After excitation, the sample containing anthracene showed a positive transient absorption at 425 nm following triplet-triplet energy transfer with an intensity of ΔAbs = +0.3885. This spectrum has been recorded after the decay of the excited state $[Ru(bpy)_3]^{2+}$. In parallel, the reference solution of $[Ru(bpy)_3]^{2+}$ showed a maximum negative signal of ΔAbs = –0.1583. Using Equation S9, we find

$$\Delta A_{Anth@425} = 0.3885$$

$$\Delta A_{Rubipy@455} = -0.1583$$

$$\Delta\varepsilon_{Anth@425} = \frac{0.3885}{-0.1583}.(-10100).\frac{1}{0.95} = 26000\ M^{-1}cm^{-1}$$

ii. Determination of the concentration of excited iridium photosensitizer, [Ir*]

A third solution containing the iridium photosensitizer with an absorption of 0.3 at 410 nm (same as the $[Ru(bpy)_3]^{2+}$ reference solution), 7.8 mM of diazene **1** and 0.1 mM of anthracene is prepared.

After light pulse, as the two solutions have the same absorption, the amount of generated excited state is assumed identical.

$$[Ir^*] = [Ru^*] = \frac{\Delta Abs}{\Delta \varepsilon} = \frac{-0.1583}{-10100} = 1.56x10^{-5}\ M$$

iii. Experimental Data

$\Delta\varepsilon_{Anth@425}$	$[Ir^*]$	$k_{q\text{-diazene}}$	$k_{q\text{-anthracene}}$	$C_{anthracene}$	$C_{diazene}$
26000 $M^{-1}.cm^{-1}$	1.56x10^{-5} M	1.45x10^{9} $M^{-1}s^{-1}$	7.76x10^{8} $M^{-1}s^{-1}$	0.1 x10^{-3} M	7.8 x10^{-3} M^{-1}

The k_q for the diazene has been taken from table 2 and the k_q for anthracene has been measured (see **Figure S59**)

iv. Quantification of maximal amount of triplet anthracene by the iridium photosensitizer

The excited-state lifetime of a photosensitizer depends on all radiative, nonradiative, and collisional decay pathways. When two quenchers (1 and 2 or anthracene and diazene) are in solution, their kinetic rate constant is directly linked to their concentration.

$$\tau = \frac{1}{k_r + k_{nr} + k_{q1}.C_1 + k_{q2}.C_2} \qquad \textbf{Eq.S10}$$

From there, we can define the quenching efficiency ϕ for a given quencher that has the same expression as **Equation S8** for two collisional decay pathways.

$$\phi_1 = \frac{k_{q1}.C_1}{k_r + k_{nr} + k_{q1}.C_1 + k_{q2}.C_2} \qquad \textbf{Eq.S11}$$

From the lifetime of the unquenched iridium complex:

$$k_r + k_{nr} = \frac{1}{\tau_0} = \frac{1}{2325x10^{-9}} = 430100\ s^{-1} \qquad \textbf{Eq.S12}$$

Using **Equations S11** and **S12**, the quenching fraction attributable to anthracene is:

$$\phi_{Anth} = \frac{k_{q-Anth}.C_{Anth}}{k_r + k_{nr} + k_{q-Anth}.C_{Anth} + k_{q-Dia}.C_{Dia}} = 0.062$$

Likewise, diazene **1** accounts for:

$$\phi_{Dia} = \frac{k_{q-Dia}.C_{Dia}}{k_r + k_{nr} + k_{q-Anth}.C_{Anth} + k_{q-Dia}.C_{Dia}} = 0.904$$

Note that the sum of the two contributions is not equal to one due to a non-absolute quenching of the photosensitizer.

By multiplying the quenching efficiency with the excited state photosensitizer concentration, we can find the theoretical maximum of photoproduct generated by a direct photoinduced reaction.

$$C_{max-photoproduct} = \phi\ x\ [Ir^*] \qquad \textbf{Eq.S13}$$

$$C_{max-{}^{T}Anth} = 0.062\ x\ 1.56\text{x}10^{-5} = 9.68\text{x}10^{-7}\ M$$

Using the Lambert-Beer equation, we can calculate the maximal signal that is detectable after a direct sensitization of anthracene by the iridium photosensitizer.

$$\Delta A_{Max\,-\,Anth@425} = \Delta \varepsilon_{Anth@425}\ x\ C_{max-{}^{T}Anth} = 9.68\text{x}10^{-7} x\ 26000 = \ 0.025$$

$$\Delta A_{Observed\,-\,Anth@425} = 0.03$$

$$\frac{\Delta A_{Observed\,-\,Anth@425}}{\Delta A_{Max\,-\,Anth@425}} = 1.20 \qquad \boldsymbol{Eq. S14}$$

With a concentration of 0.1 mM of anthracene and 7.8 mM of diazene **1**, the signal detected is 20% higher than the theoretical maximum, thereby providing clear evidence for the mediator-enhanced triplet energy transfer process before the fragmentation of the diazene and confirming that initial energy transfer from $[Ir(dFCF_3ppy)_2(dtb)]^+$ to the diazene occurs.

Following the same methodology, we provide measurements at different concentrations of diazene **1** and the results are tabulated in **Table S1**. Overall, in a range between 2.8 and 7.8 mM of diazene **1** the signal detected is 21% higher than the theoretical maximum.

Table S1: Results from the mediator-enhanced triplet energy transfer strategy carried out at different concentrations of diazene **1.** The measured ΔAbs were determined using **Figure S58**.

**Concentration of diazene 1 (x10^{-3} M)**	**0**	**2.8**	**5.4**	**7.8**
Concentration of Anthracene (x10^{-3} M)	0.1	0.1	0.1	0.1
ΔAbs measured at 425 nm	0.26	0.07	0.043	0.03
Theoretical maximum ΔAbs calculated	0.261	0.058	0.034	0.025
Ratio using **Equation S14**	0.99	1.21	1.26	1.20

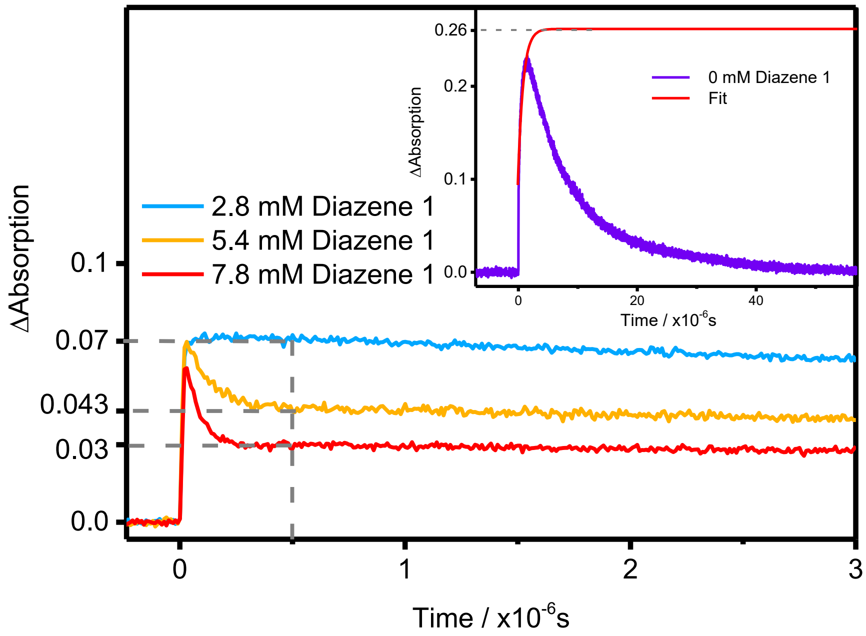

Figure S59: Single wavelength absorption changes of [Ir(dFCF$_3$ppy)$_2$(dtb)]+ (**PC-10**) (λ_{det} = 425 nm, λ_{exc} = 450 nm) in Ar-saturated acetonitrile in the presence of 0.1 mM of Anthracene and diazene **1** at different concentrations. The maximal signal of the triplet Anthracene is measured after the excited state decay of **PC-10** (Pale grey, dashed line). Inset: Single wavelength absorption changes of [Ir(dFCF$_3$ppy)$_2$(dtb)]+ (**PC-10**) (λ_{det} = 425 nm, λ_{exc} = 450 nm) in Ar-saturated acetonitrile in the presence of 0.1 mM of Anthracene. The maximum signal of the triplet Anthracene is measured by fitting the signal growth until a plateau is reached.

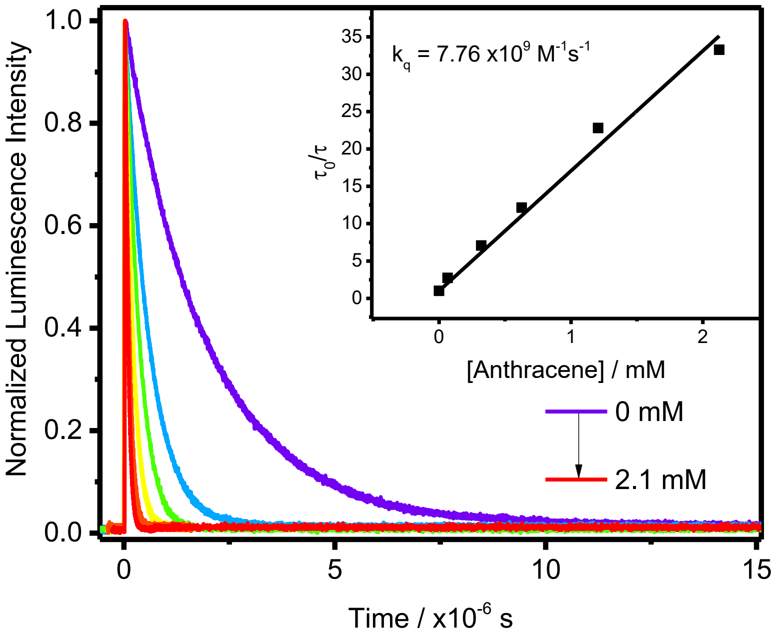

Figure S60: Normalized time-resolved emission measurements of [Ir(dFCF$_3$ppy)$_2$(dtb)]+ (**PC-10**) (λ_{det} = 495 nm, λ_{exc} = 450 nm) recorded in Ar-saturated acetonitrile in the presence of anthracene at different concentrations. Inset: Corresponding Stern-Volmer plot with linear fit from which the quenching rate constant was estimated.

IX. Reaction Progress Monitoring via ^{1}H NMR

Product distribution under photosensitized fragmentation of diazene 3:

A flame-dried and argon-purged 4 mL vial was charged with **PC-10** (1.2 mg, 1 mol%). The vial was then evacuated under high vacuum for 15 min and then backfilled with argon. Anhydrous degassed CD_3CN (0.6 mL) was added with a syringe under an argon atmosphere. Finally, diazene **3** (25 mg, 0.10 mmol) was added with a microsyringe. 17 µL (0.10 mmol, 1.0 equiv) of $PhSiMe_3$ was added as an NMR internal standard to monitor the reaction progress through ^{1}H NMR. The resulting solution was further sparged for 10 mins with argon. The vial was then taken into a nitrogen-filled glovebox, where the homogeneous reaction mixture was transferred to a J. Young NMR tube. The NMR tube was sealed inside the glovebox, removed, and irradiated with a Kessil lamp (PR160L, 30 W, 456 nm) operated at 25X intensity. *The distance between the lamp and the NMR tube was maintained at 5 cm throughout the experiment.* A fan was placed next to the setup to ensure adequate heat dissipation and maintain the reaction at room temperature. For each timepoint, the concentration of each species was calculated by comparison to the methyl signal of the internal standard (see labeled peaks in **Figure S62**). Due to peak overlap, the concentration of **6b** could not be estimated using the corresponding Me signals, however integration of the separate benzylic hydrogens of diastereomers **6a** and **6b** indicated that both are formed in a 1:1 ratio consistent with the literature.[7]

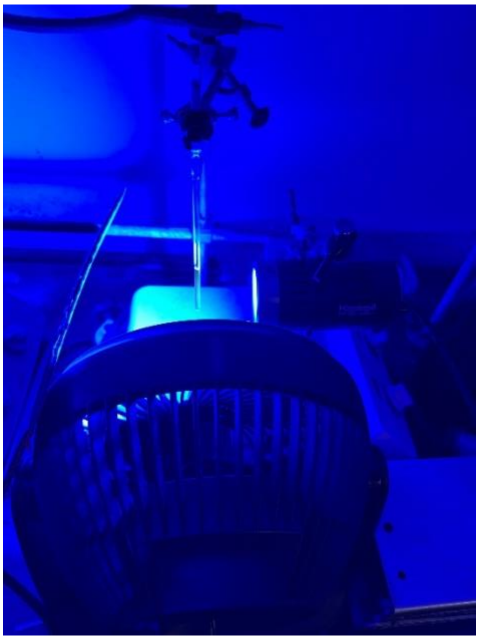

Figure S61: Setup for NMR kinetics experiments: Reaction monitoring in a closed J. Young NMR tube irradiated with a Kessil lamp (PR160L, 30 W, 456 nm, placed 5 cm to the tube).

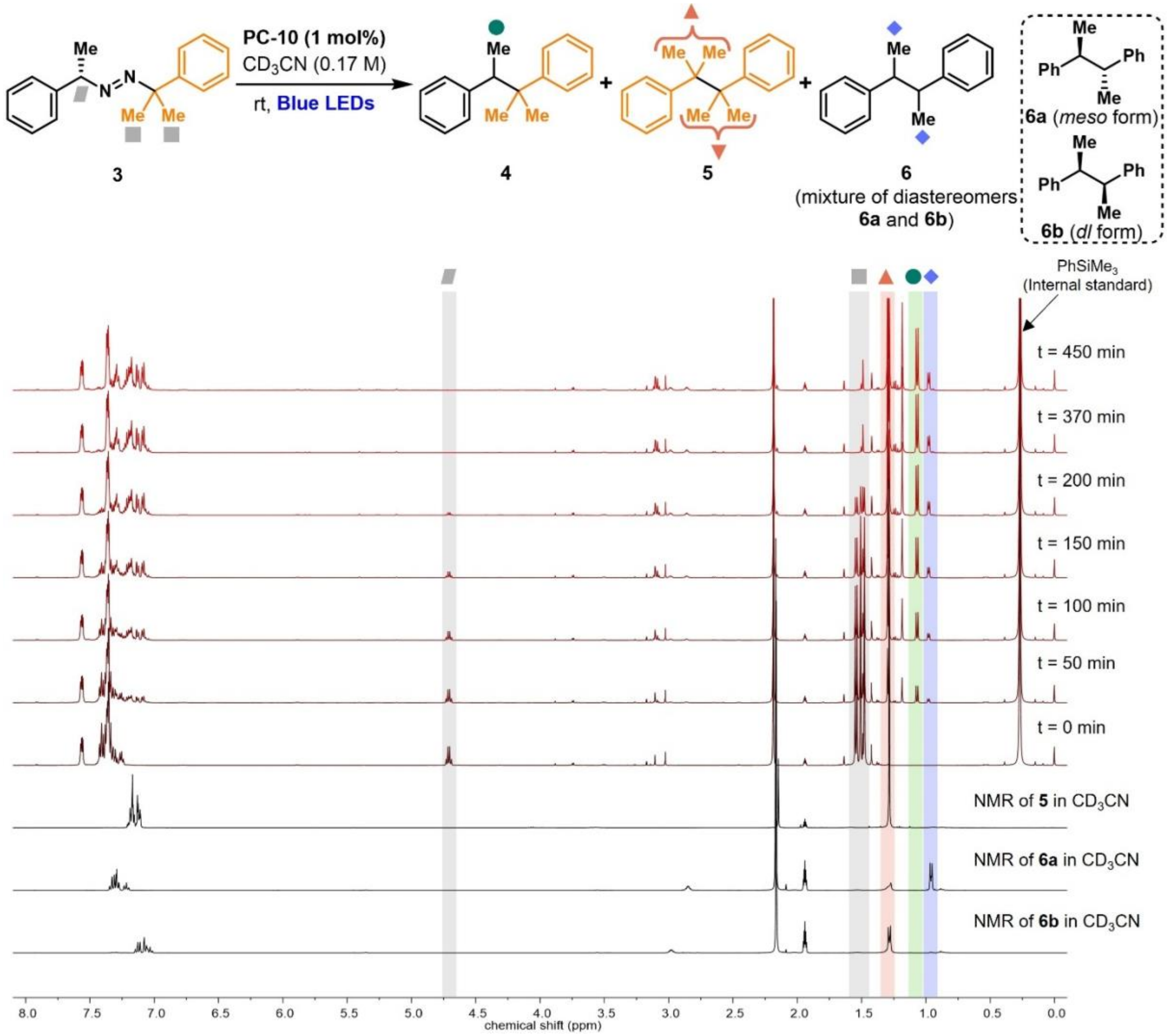

Figure S62: Monitoring of photofragmentation of **3** under blue light in the presence of **PC-10** via ^{1}H NMR spectroscopy. Characteristic peaks are highlighted. ^{1}H NMR spectra of isolated products **5**, **6a** and **6b** in CD_3CN are stacked below for comparison. ^{1}H NMR data for product **4** is consistent with the literature.[25]

Product distribution under UV irradiation of diazene **3**:

Anhydrous degassed CD_3CN (0.6 mL) was added with a syringe to a flame-dried and argon-purged 4 mL vial under an argon atmosphere. Diazene **3** (25 mg, 0.10 mmol) was added with a microsyringe. 17 µL (0.10 mmol, 1.0 equiv) of $PhSiMe_3$ was added as an NMR internal standard to monitor the reaction progress through ^{1}H NMR. The resulting solution was then sparged for 10 min with argon. The vial was then taken into a nitrogen-filled glovebox, where the homogeneous reaction mixture was transferred to a J. Young NMR tube. The NMR tube was sealed inside the glovebox, removed, and irradiated with two 350 nm Rayonet UV lamps (RPR-3500A). *The distance between the lamps and the NMR tube was maintained at 2 cm throughout the experiment*. A fan was placed next to the setup to ensure adequate heat dissipation and maintain the reaction at room temperature. For each timepoint, the concentration of

each species was calculated by comparison to the methyl signal of the internal standard (see labeled peaks in **Figure S63**). Due to peak overlap, the concentration of **6b** could not be estimated using the corresponding Me signals, however integration of the separate benzylic hydrogens of diastereomers **6a** and **6b** indicated that both are formed in a 1:1 ratio as in the photosensitized conditions.

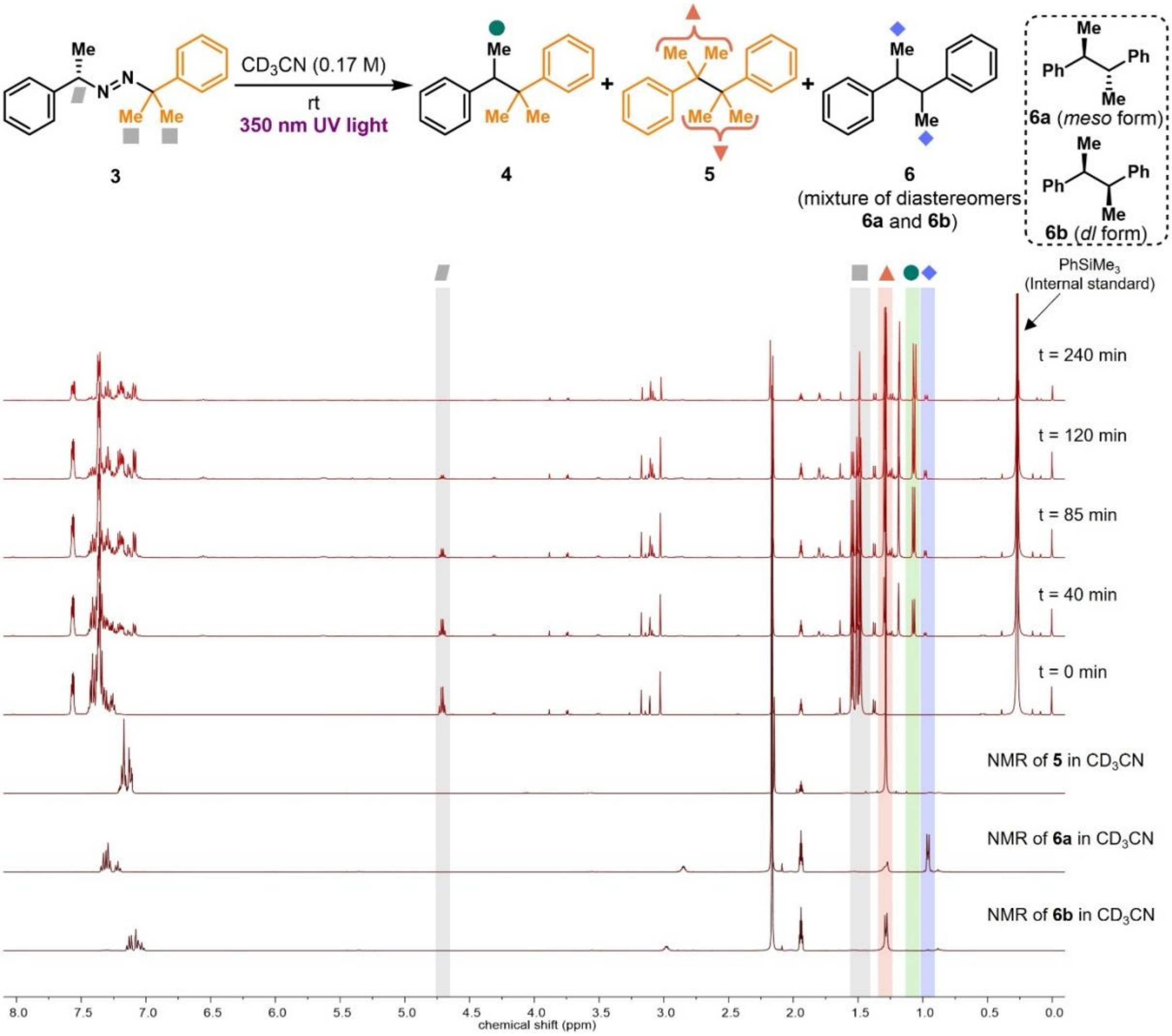

Figure S63: Monitoring of photofragmentation of **3** under UV irradiation. Characteristic peaks are highlighted. ^{1}H NMR spectra of isolated products **5**, **6a** and **6b** in CD_3CN are stacked below for comparison. ^{1}H NMR data for product **4** are consistent with the literature.[25]

Competitive photofragmentation of various pairs of 1,2-dialkyldiazenes:

A flame-dried and argon-purged 4 mL vial was charged with **PC-10** (2.4 mg, 2 mol%). The vial was then evacuated under high vacuum for 15 min and then backfilled with argon. Anhydrous degassed CD_3CN (0.6 mL) was added with a syringe under an argon atmosphere. Finally, the two diazenes (0.10 mmol each) were added sequentially using a microsyringe. 17 µL (0.10 mmol, 1.0 equiv) of $PhSiMe_3$ was added as an NMR internal standard to monitor the reaction progress through ^{1}H NMR. The resulting solution was then sparged for 10 min with argon. The vial was then taken into a nitrogen-filled glovebox,

where the homogeneous reaction mixture was transferred to a J. Young NMR tube. The NMR tube was sealed inside the glovebox, removed, and irradiated with a Kessil lamp (PR160L, 30 W, 456 nm) operated at 100X intensity. *The distance between the lamp and the NMR tube was maintained at 5 cm throughout the experiment.* A fan was placed next to the setup to ensure adequate heat dissipation and maintain the reaction at room temperature. For each timepoint, the concentration of each diazene was calculated by comparison to the methyl signal of the internal standard (see labeled peaks in **Figures S64** and **S65**).

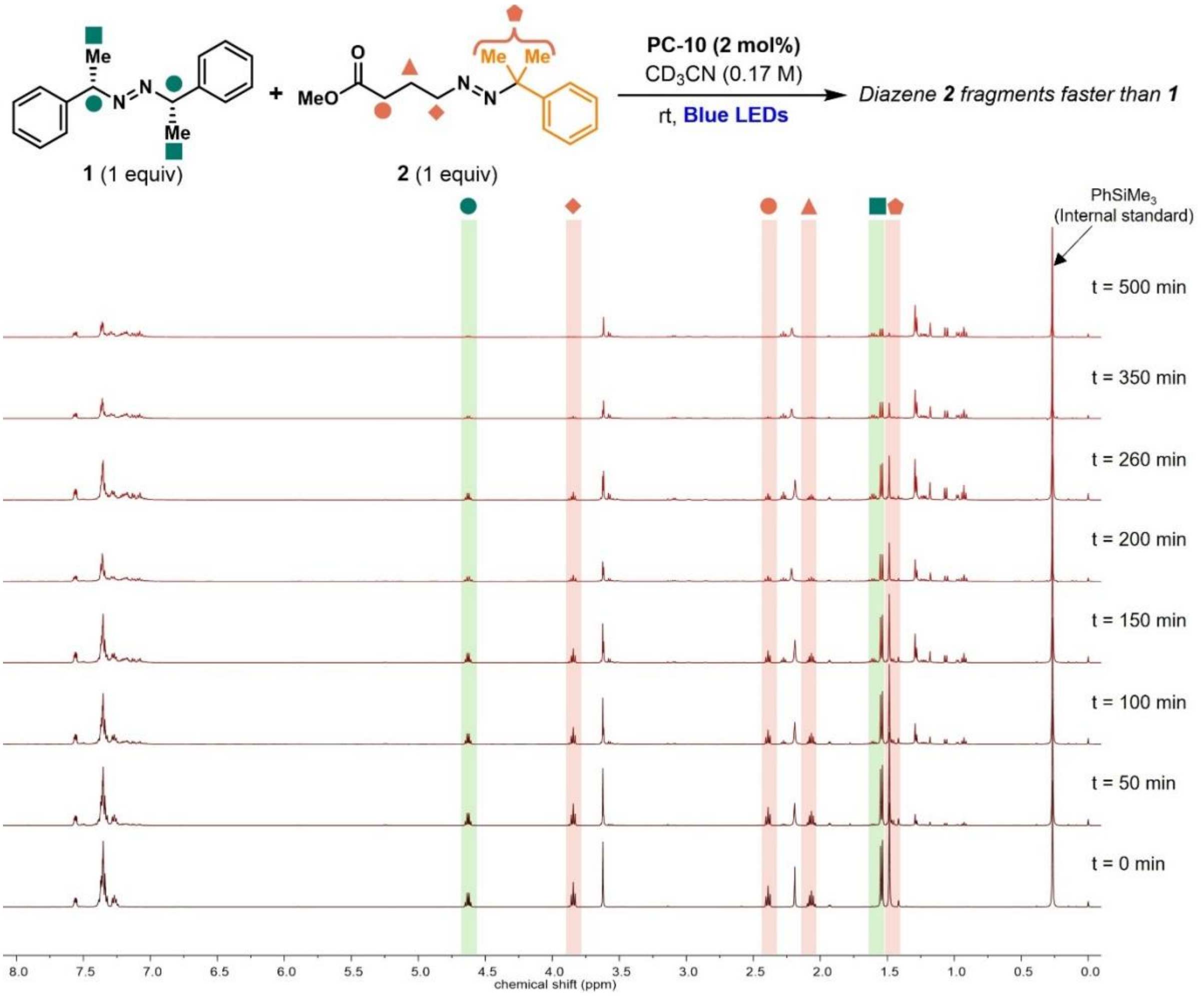

Figure S64: Monitoring of competitive photofragmentation of diazene **1** and **2** under blue light in the presence of **PC-10** via ^{1}H NMR spectroscopy. Characteristic peaks are highlighted.

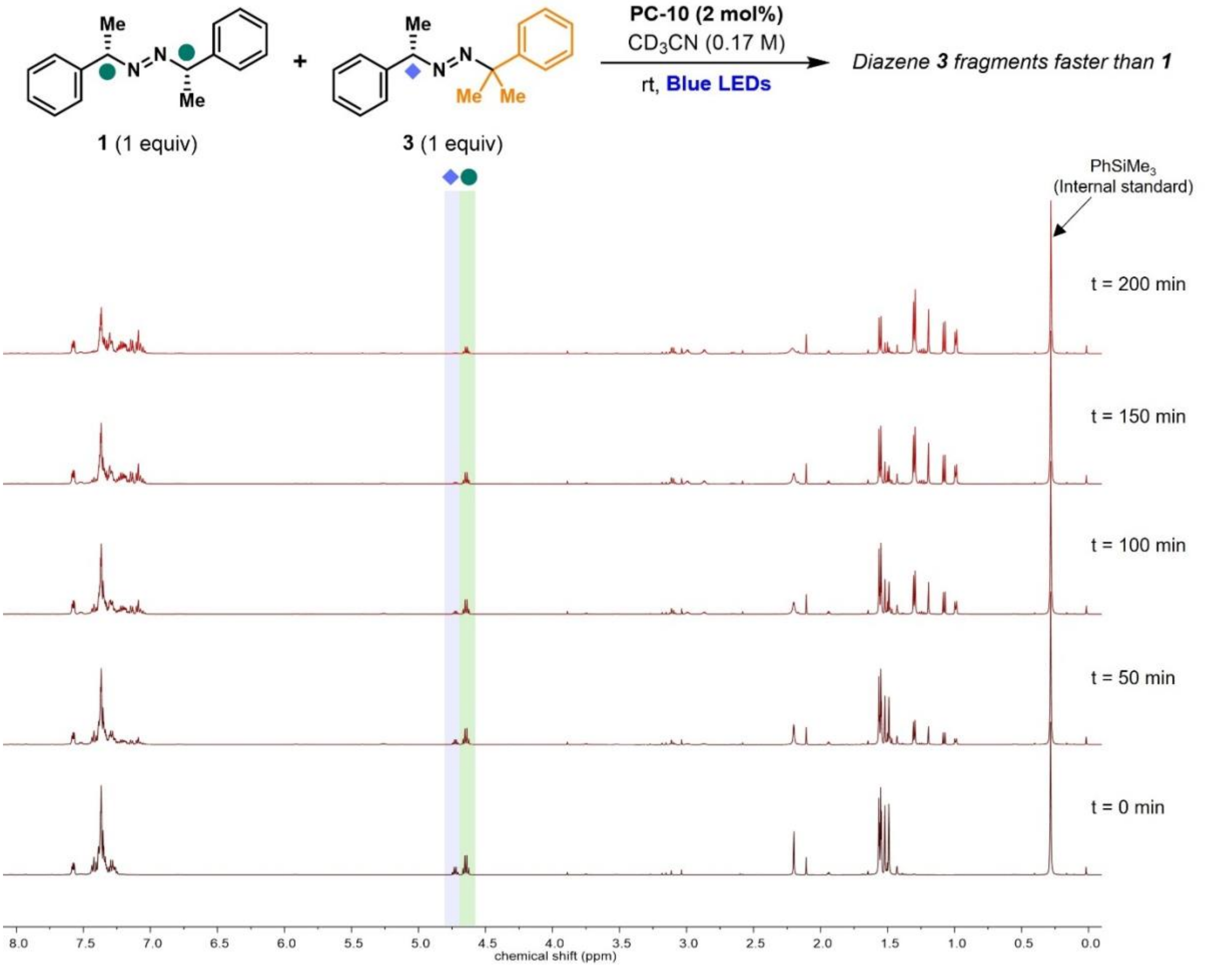

Figure S65: Monitoring of competitive photofragmentation of diazene **1** and **3** under blue light in the presence of **PC-10** via ^{1}H NMR spectroscopy. Characteristic peaks are highlighted.

Photofragmentation kinetics of diazene **2** with photocatalysts of different triplet energy:

A flame-dried and argon-purged 4 mL vial was charged with appropriate photocatalyst (**PC**) (1 mol%). The vial was then evacuated under high vacuum for 15 min and then backfilled with argon. Anhydrous degassed CD_3CN (0.6 mL) was added with a syringe under an argon atmosphere. Finally, diazene **2** (25 mg, 0.10 mmol) was added using a microsyringe. 17 µL (0.10 mmol, 1.0 equiv) of $PhSiMe_3$ was added as an NMR internal standard to monitor the reaction progress through ^{1}H NMR. The resulting solution was then sparged for 10 min with argon. The vial was then taken into a nitrogen-filled glovebox, where the homogeneous reaction mixture was transferred to a J. Young NMR tube. The NMR tube was sealed inside the glovebox, removed, and irradiated with a Kessil lamp (PR160L, 30 W, 456 nm) operated at 100X intensity. *The distance between the lamp and the NMR tube was maintained at 5 cm throughout the experiment*. A fan was placed next to the setup to ensure adequate heat dissipation and maintain the reaction at room temperature. For each timepoint, the concentration of the diazene was calculated by comparison to the methyl signal of the internal standard (see labeled peaks in **Figures S66** and **S67**).

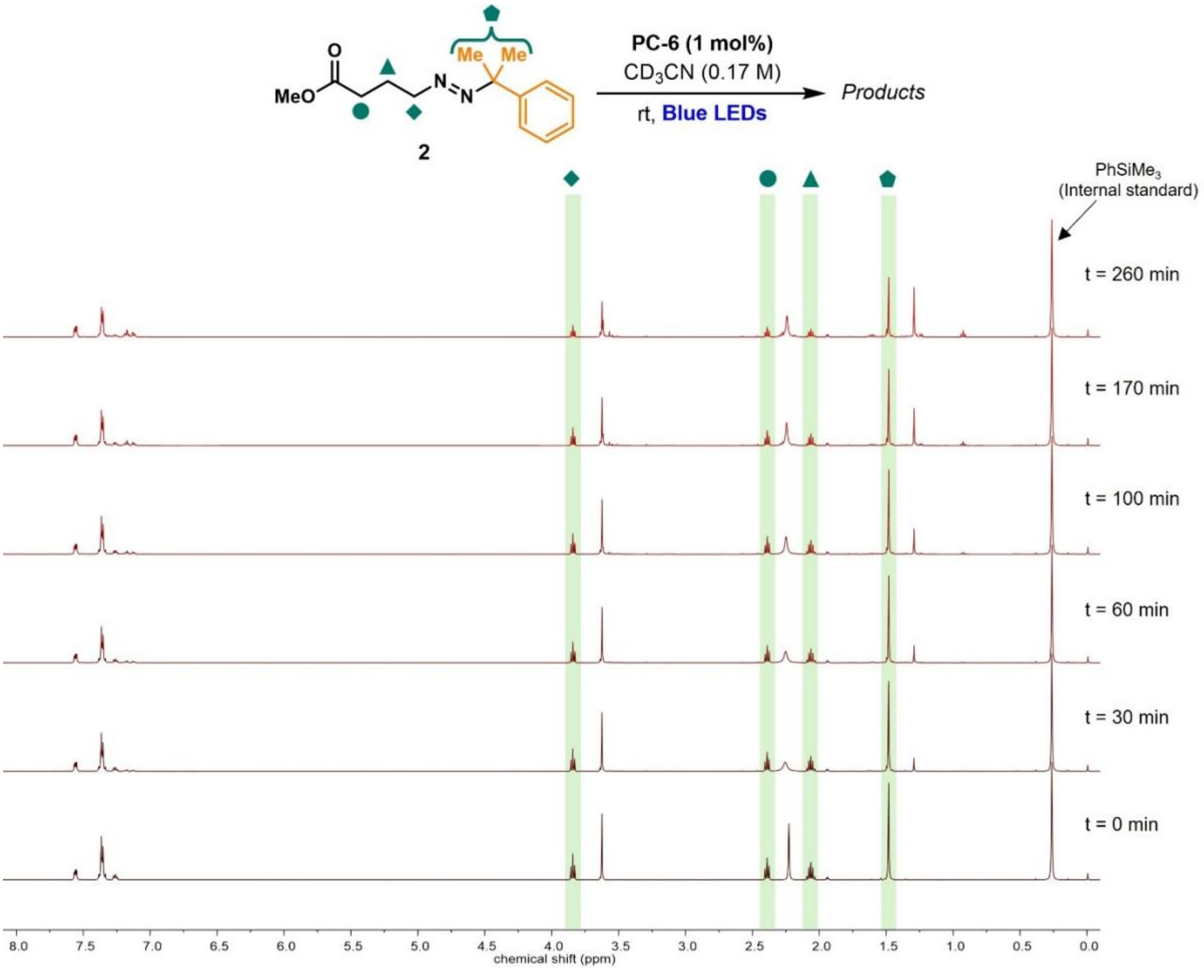

Figure S66: Monitoring of photofragmentation of **2** under blue light in presence of **PC-6** via ^{1}H NMR spectroscopy. Characteristic peaks are highlighted.

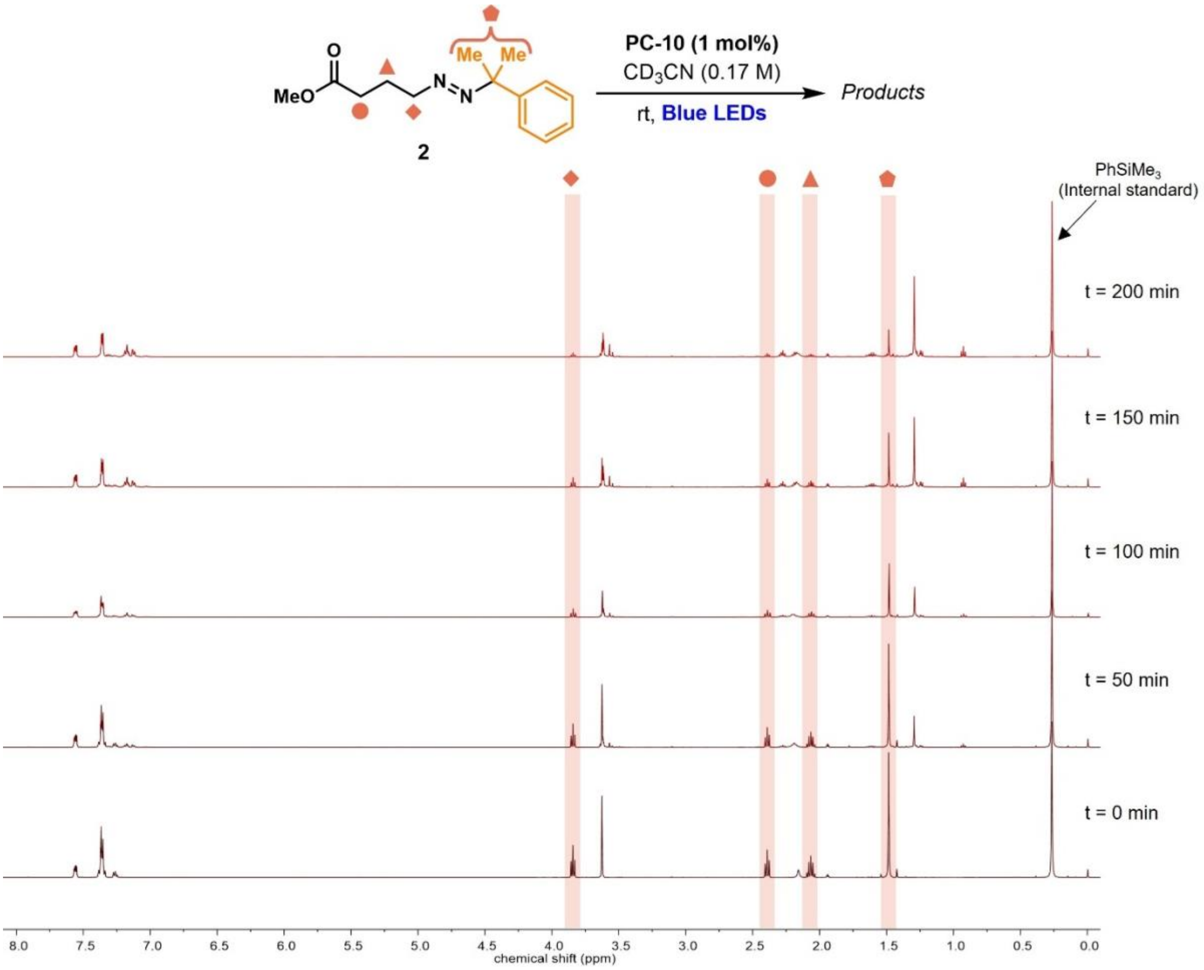

Figure S67: Monitoring of photofragmentation of **2** under blue light in presence of **PC-10** via ^{1}H NMR spectroscopy. Characteristic peaks are highlighted.

X. Value of λ in the Rehm-Weller plot

In the present study, λ was initially optimized and subsequently fixed to 1.0 eV for all photocatalysts in order to minimize the number of adjustable parameters and to enable meaningful comparison across the photocatalyst series. Importantly, variation of λ within a physically reasonable range (0.5-1.5 eV) significantly impacts the quality of the Rehm-Weller fits and, consequently, the extracted triplet energy of the diazene. This sensitivity is illustrated in **Figure S68**, which shows that a reorganization energy of 1.0 eV affords the best overall agreement with the experimental data. This value represents a commonly accepted value accounting for the combined inner- and outer-sphere contributions in bimolecular electron- and energy-transfer processes in solution.[26-30]

For the purpose of this analysis, λ was treated as constant across the 15 photocatalysts examined. While variations in reorganization energy between different photocatalysts cannot be strictly excluded, an ideal scenario in which a single family of photosensitizers spans the entire relevant triplet-energy range is rarely achievable. Accordingly, λ is treated here as an effective average parameter that reflects solvent reorganization (acetonitrile), encounter-complex formation, and excited-state relaxation processes common to the entire photocatalyst series. Given that all measurements were performed in the same solvent, at room temperature, and with an identical quencher, the assumption of a constant effective λ constitutes a reasonable and widely adopted approximation within the empirical Rehm-Weller framework.

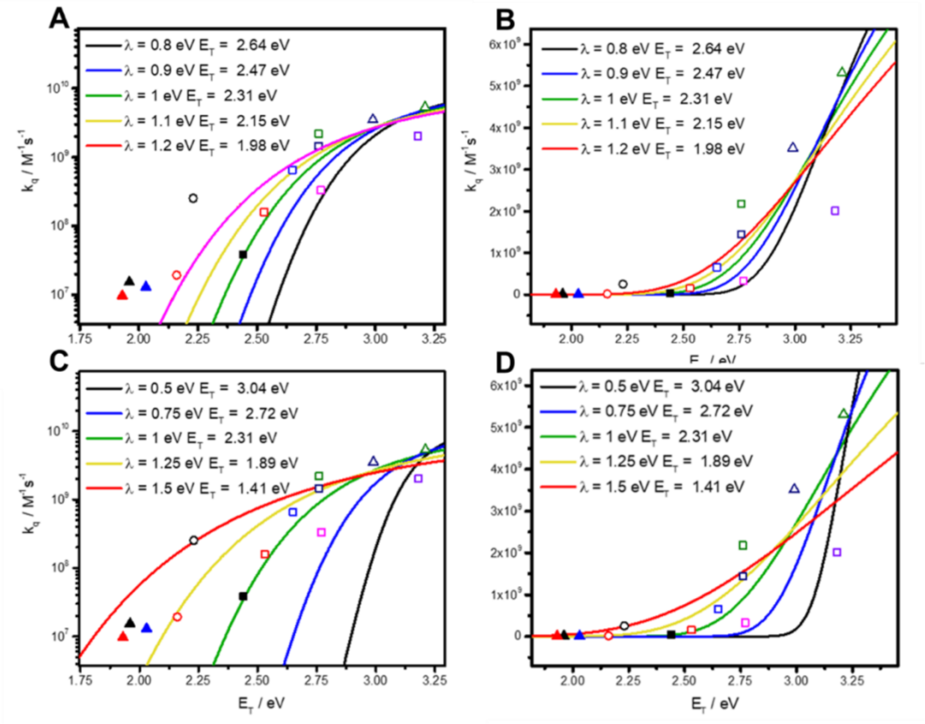

Figure S68: Influence of the reorganization energy (λ) on the Rehm-Weller analysis and the extracted triplet energy of diazene **1**. Rehm-Weller fits obtained using λ values ranging from 0.8 to 1.2 eV, displayed on (A) a logarithmic scale and (B) on a linear scale. Rehm-Weller fits obtained using λ values ranging from 0.5 to 1.5 eV, displayed on (C) a logarithmic scale and (D) on a linear scale.

XI. References

1. Cusumano, A. Q.; Chaffin, B. C.; Doyle, A. G., Mechanism of Ni-Catalyzed Photochemical Halogen Atom-Mediated C(Sp^3)–H Arylation. *J. Am. Chem. Soc.* **2024,** *146*, 15331-15344.
2. Smith, C. R., Activated Zinc Dust. *Synlett* **2009,** *2009*, 1522-1523.
3. Hartl, F.; Luyten, H.; Nieuwenhuis, H. A.; Schoemaker, G. C., Versatile Cryostated Optically Transparent Thin-Layer Electrochemical (Ottle) Cell for Variable-Temperature Uv-Vis/Ir Spectroelectrochemical Studies. *Appl. Spectrosc.* **1994,** *48*, 1522-1528.
4. Mahabiersing, T.; Luyten, H.; Nieuwendam, R. C.; Hartl, F., Synthesis, Spectroscopy and Spectroelectrochemistry of Chlorocarbonyl {1,2-Bis[(2,6-Diisopropylphenyl)Imino]Acenaphthene-κ^2-N,N'}Rhodium(I). *Collection of Czechoslovak Chemical Communications* **2003,** *68*, 1687-1709.
5. Krejčik, M.; Daněk, M.; Hartl, F., Simple Construction of an Infrared Optically Transparent Thin-Layer Electrochemical Cell. *J. Electroanal. Chem.* **1991,** *317*, 179-187.
6. Chattapadhyay, D.; Liu, E.-C.; Diaz, M. J.; Maity, A.; Bratten, B. A.; Michaudel, Q., Radical Sorting as a General Framework for Deaminative C(Sp^3)–C(Sp^2) Cross-Coupling. *Chem* **2025**, 102716.
7. Chattapadhyay, D.; Aydogan, A.; Doktor, K.; Maity, A.; Wu, J. W.; Michaudel, Q., Harnessing Sulfur(Vi) Fluoride Exchange Click Chemistry and Photocatalysis for Deaminative Benzylic Arylation. *ACS Catal.* **2023,** *13*, 7263-7268.
8. Yasu, Y.; Koike, T.; Akita, M., Visible Light-Induced Selective Generation of Radicals from Organoborates by Photoredox Catalysis. *Adv. Synth. Catal.* **2012,** *354*, 3414-3420.
9. Yan, C.-S.; Peng, Y.; Xu, X.-B.; Wang, Y.-W., Nickel-Mediated Inter- and Intramolecular Reductive Cross-Coupling of Unactivated Alkyl Bromides and Aryl Iodides at Room Temperature. *Chem-Eur. J.* **2012,** *18*, 6039-6048.
10. Luo, J.; Davenport, M. T.; Ess, D. H.; Liu, T. L., Electro/Ni Dual-Catalyzed Decarboxylative C(Sp^3)−C(Sp^2) Cross-Coupling Reactions of Carboxylates and Aryl Bromide. *Angew. Chem. Int. Ed.* **2024,** *63*, e202403844.
11. Qin, Q.; Wang, W.; Zhang, C.; Song, S.; Jiao, N., A Metal-Free Desulfurizing Radical Reductive C–C Coupling of Thiols and Alkenes. *Chem. Commun.* **2019,** *55*, 10583-10586.
12. Wang, J.; Ehehalt, L. E.; Huang, Z.; Beleh, O. M.; Guzei, I. A.; Weix, D. J., Formation of C(Sp^2)–C(Sp^3) Bonds Instead of Amide C–N Bonds from Carboxylic Acid and Amine Substrate Pools by Decarbonylative Cross-Electrophile Coupling. *J. Am. Chem. Soc.* **2023,** *145*, 9951-9958.
13. Manvar, A.; Fleming, P.; O'Shea, D. F., General Ambient Temperature Benzylic Metalations Using Mixed-Metal Li/K-Tmp Amide. *J. Org. Chem.* **2015,** *80*, 8727-8738.
14. Bastick, K. A.; Watson, A. J., Pd-Catalyzed Homologation of Arylboronic Acids as a Platform for the Diversity-Oriented Synthesis of Benzylic C–X Bonds. *Synlett* **2023,** *34*, 2097-2102.
15. Du, W.; Zhao, F.; Yang, R.; Xia, Z., Gold-Catalyzed C(Sp^3)–C(Sp^2) Suzuki–Miyaura Coupling Reaction. *Org. Lett.* **2024,** *26*, 3145-3150.
16. Rehm, D.; Weller, A., Kinetik Und Mechanismus Der Elektronübertragung Bei Der Fluoreszenzlöschung in Acetonitril. *Berich. Bunsen. Gesell.* **1969,** *73*, 834-839.
17. Marcus, R. A., On the Theory of Oxidation-Reduction Reactions Involving Electron Transfer. I. *J. Chem. Phys.* **1956,** *24*, 966-978.
18. Valeur, B.; Berberan-Santos, M. N., *Molecular Fluorescence*. 2012.
19. Montalti, M.; Credi, A.; Prodi, L.; Gandolfi, M. T., *Handbook of Photochemistry*. 2006.
20. Vauthey, E., Effect of Steric Hindrance on the Dynamics of Charge Recombination within Geminate Ion Pairs. *J. Phys. Chem. A* **2000,** *104*, 1804-1810.
21. Sandros, K.; Haglid, F.; Ryhage, R.; Ryhage, R.; Stevens, R., Transfer of Triplet State Energy in Fluid Solutions. Iii. Reversible Energy Transfer. *Acta Chem. Scand.* **1964,** *18*, 2355-2374.
22. Balzani, V.; Bolletta, F.; Scandola, F., Vertical and "Nonvertical" Energy Transfer Processes. A General Classical Treatment. *J. Am. Chem. Soc.* **2002,** *102*, 2152-2163.
23. Muller, P.; Brettel, K., $[Ru(Bpy)_3]^{2+}$ as a Reference in Transient Absorption Spectroscopy: Differential Absorption Coefficients for Formation of the Long-Lived ^{3}MLCT Excited State. *Photochem. Photobiol. Sci.* **2012,** *11*, 632-6.

24. Kerzig, C.; Goez, M., Combining Energy and Electron Transfer in a Supramolecular Environment for the "Green" Generation and Utilization of Hydrated Electrons through Photoredox Catalysis. *Chem. Sci.* **2016,** *7*, 3862-3868.
25. Bellos, K.; Stamm, H., Two-Step 1,2-Shifts by β-Cleavage of Carbenium Ions and Recombination in Friedel−Crafts Reactions of 2-Tert-Butyl-1-Tosylaziridines1. *J. Org. Chem.* **1998,** *63*, 7749-7752.
26. Pfund, B.; Wenger, O. S., Excited Organic Radicals in Photoredox Catalysis. *JACS Au* **2025,** *5*, 426-447.
27. Bangle, R. E.; Schneider, J.; Piechota, E. J.; Troian-Gautier, L.; Meyer, G. J., Electron Transfer Reorganization Energies in the Electrode-Electrolyte Double Layer. *J. Am. Chem. Soc.* **2020,** *142*, 674-679.
28. Gray, H. B.; Winkler, J. R., Long-Range Electron Transfer. *Proc. Natl. Acad. Sci. U S A* **2005,** *102*, 3534-9.
29. Kumpulainen, T.; Lang, B.; Rosspeintner, A.; Vauthey, E., Ultrafast Elementary Photochemical Processes of Organic Molecules in Liquid Solution. *Chem. Rev.* **2017,** *117*, 10826-10939.
30. Jayanthi, S. S.; Ramamurthy, P., Photoinduced Electron Transfer Reactions of 2,4,6-Triphenylpyrylium: Solvent Effect and Charge-Shift Type of Systems. *Phys. Chem. Chem. Phys.* **1999,** *1*, 4751-4757.

XII. NMR Spectroscopy

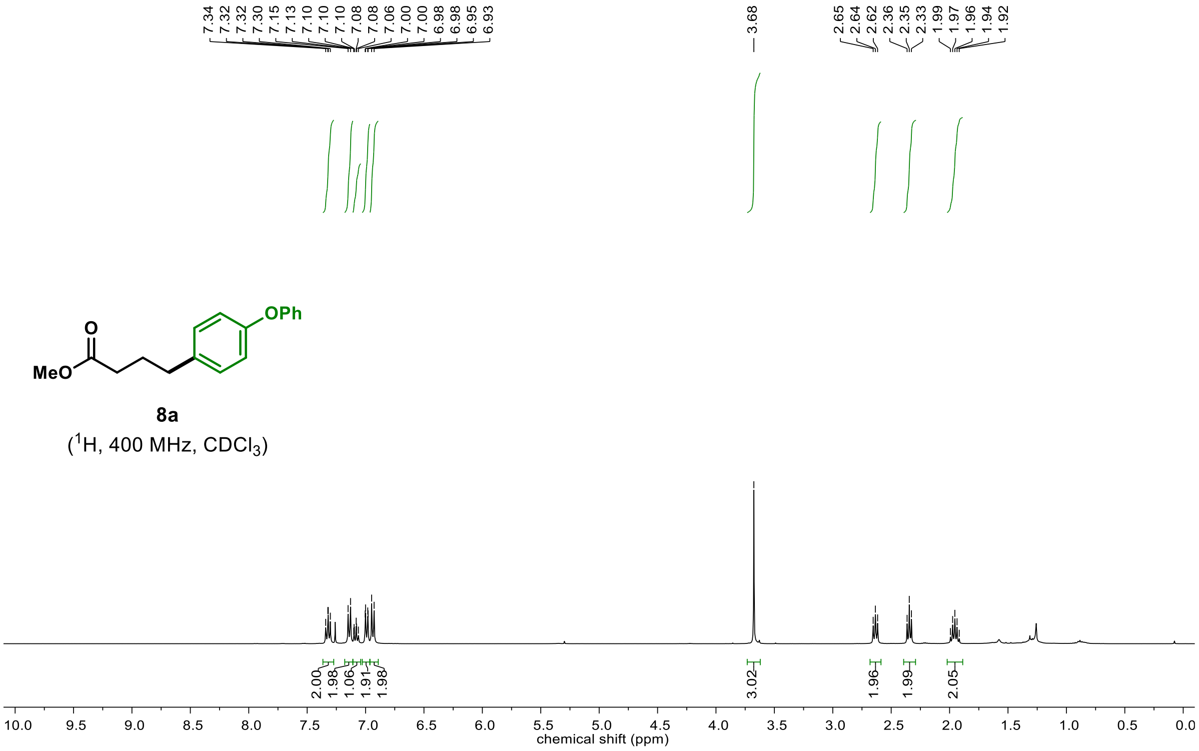

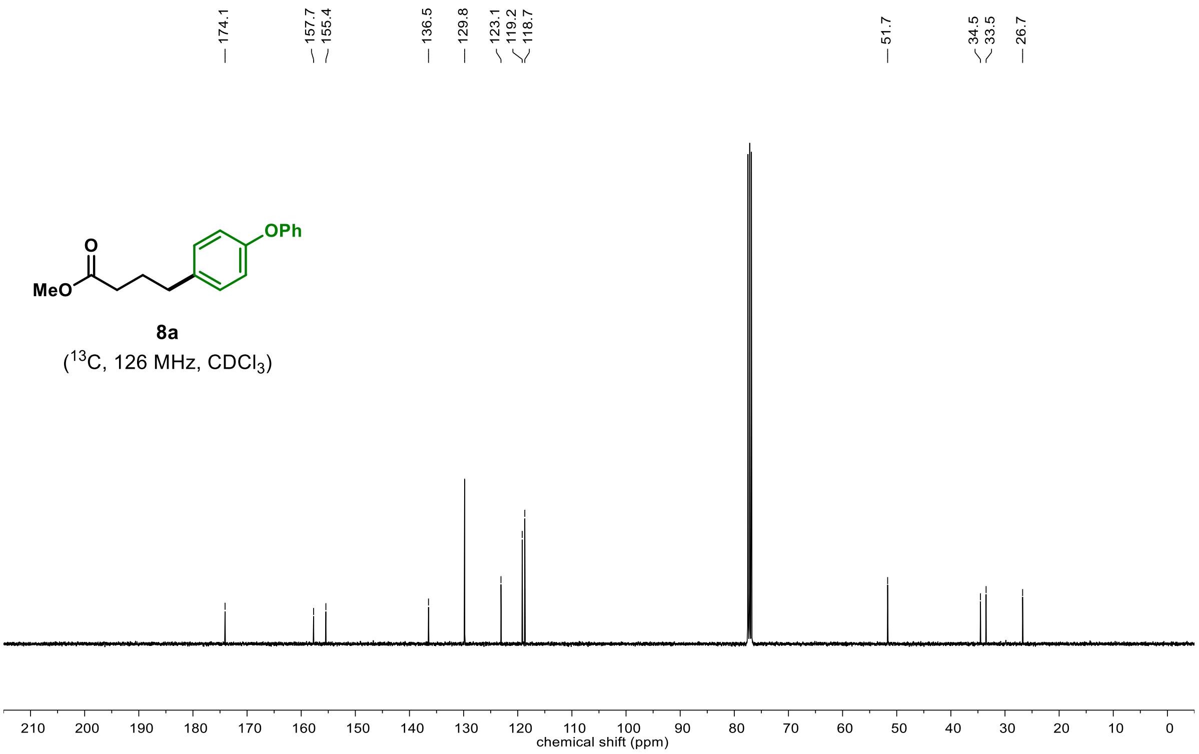

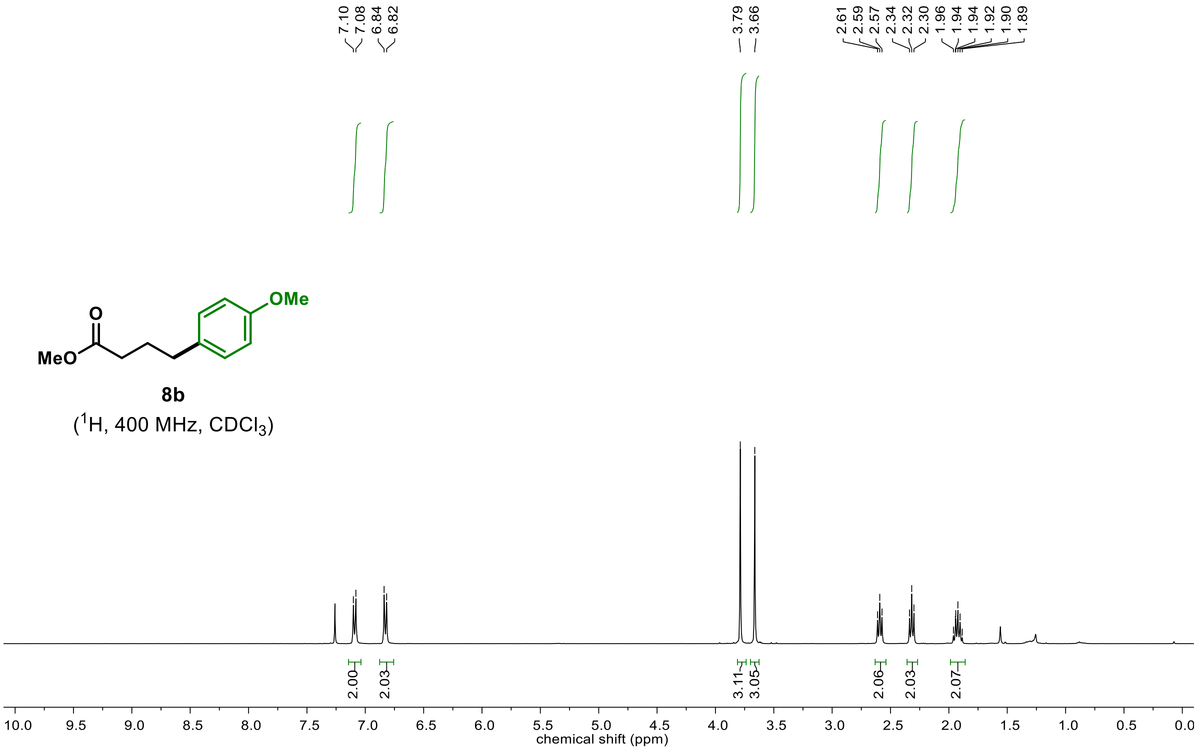

8b
(^{1}H, 400 MHz, $CDCl_3$)

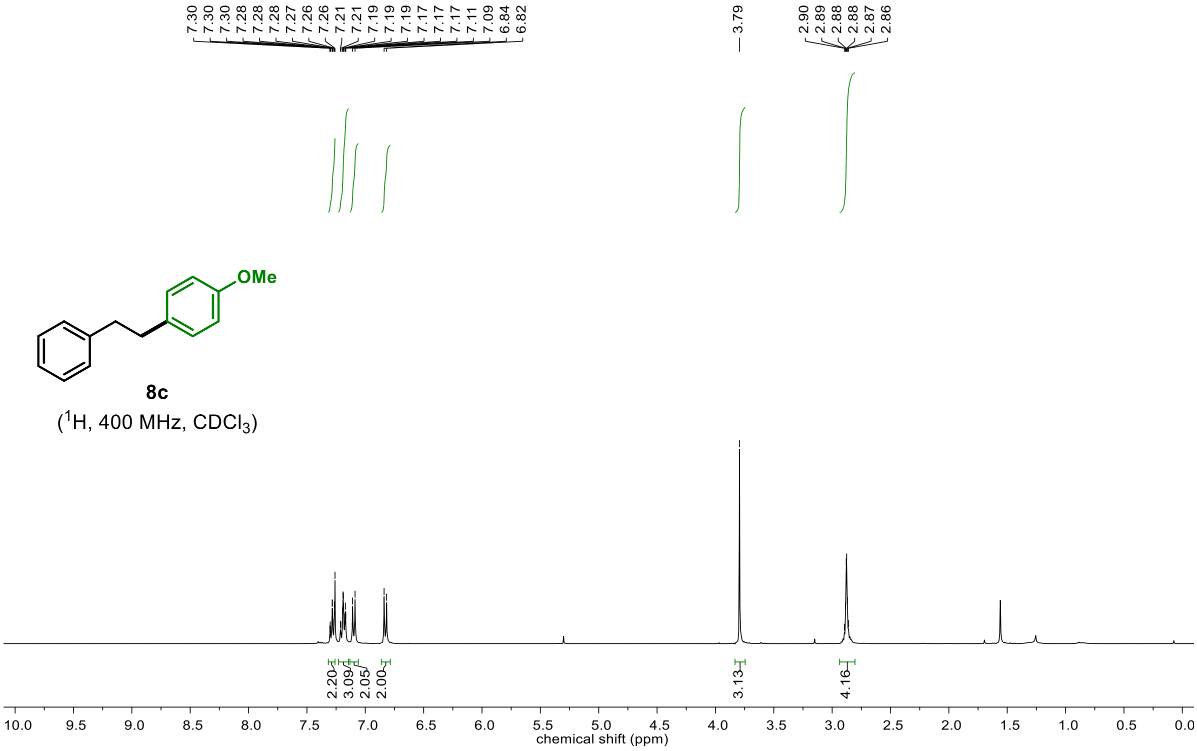

8c
(^{1}H, 400 MHz, $CDCl_3$)

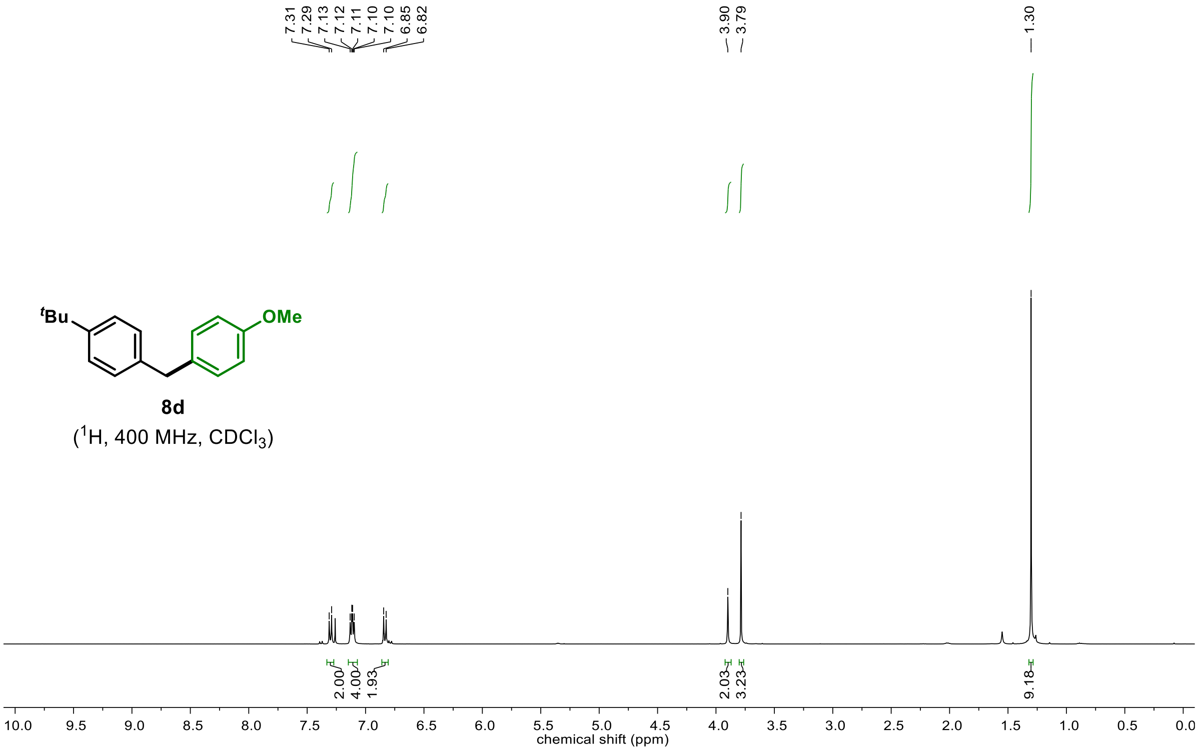
7.31
7.29
7.13
7.12
7.11
7.10
7.10
6.85
6.82
3.90
3.79
1.30
tBu
OMe
8d
(1H, 400 MHz, CDCl3)
2.00
4.00
1.93
2.03
3.23
9.18
10.0 9.5 9.0 8.5 8.0 7.5 7.0 6.5 6.0 5.5 5.0 4.5 4.0 3.5 3.0 2.5 2.0 1.5 1.0 0.5 0.0
chemical shift (ppm)

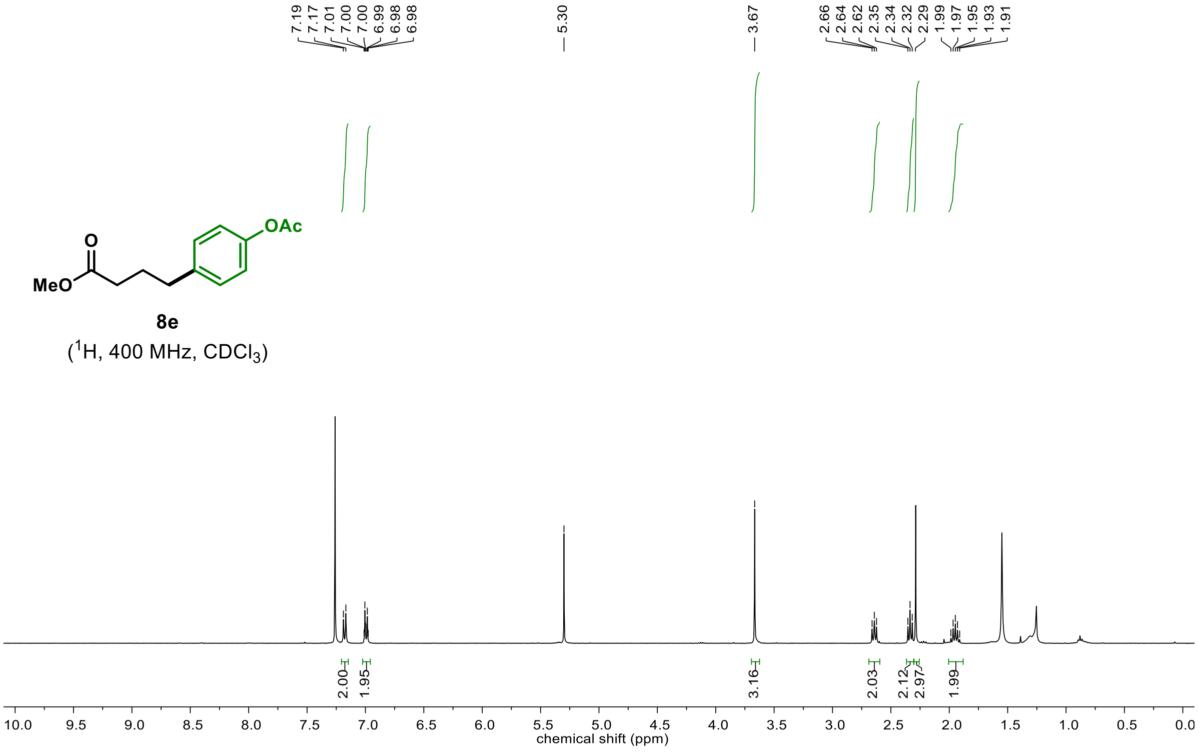
7.19
7.17
7.01
7.00
7.00
6.99
6.98
6.98
5.30
3.67
2.66
2.64
2.62
2.35
2.34
2.32
2.29
1.99
1.97
1.95
1.93
1.91
O
MeO
OAc
8e
(1H, 400 MHz, CDCl3)
2.00
1.95
3.16
2.03
2.12
2.97
1.99
10.0 9.5 9.0 8.5 8.0 7.5 7.0 6.5 6.0 5.5 5.0 4.5 4.0 3.5 3.0 2.5 2.0 1.5 1.0 0.5 0.0
chemical shift (ppm)

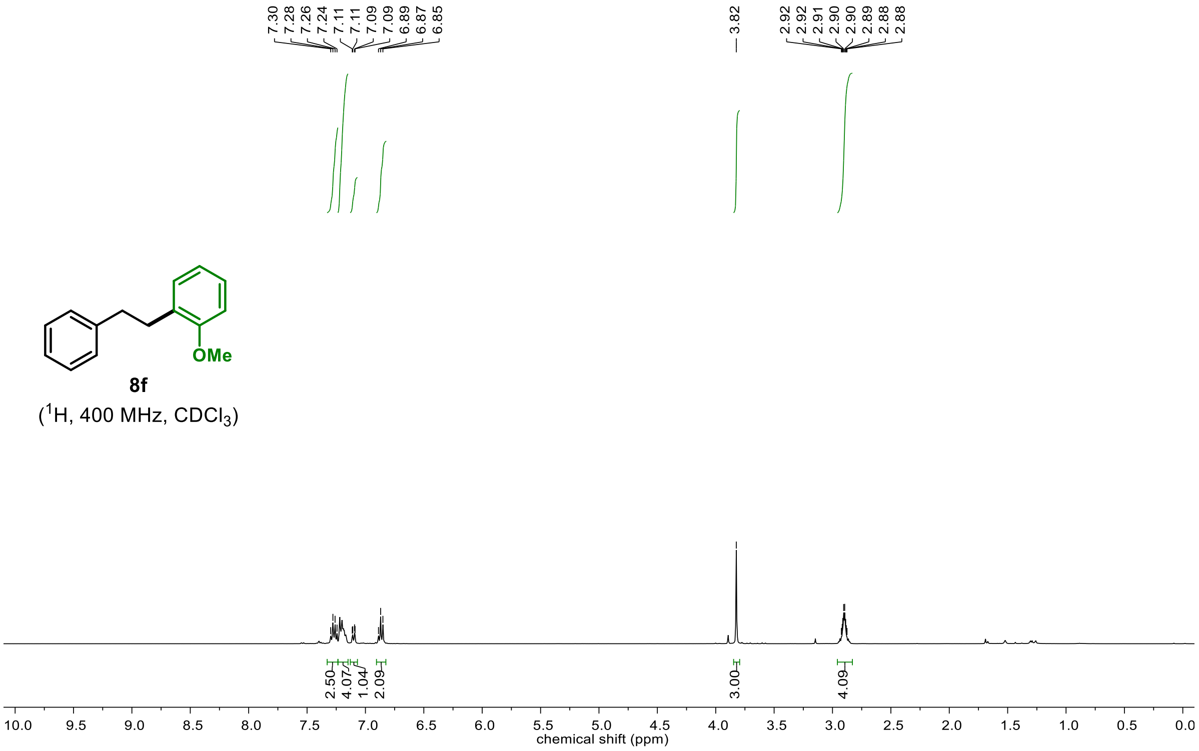
7.30 7.28 7.26 7.24 7.11 7.11 7.09 6.89 6.87 6.85
3.82
2.92 2.92 2.91 2.90 2.90 2.89 2.88 2.88
OMe
8f
(^{1}H, 400 MHz, CDCl$_3$)
2.50 4.07 1.04 2.09
3.00
4.09
10.0 9.5 9.0 8.5 8.0 7.5 7.0 6.5 6.0 5.5 5.0 4.5 4.0 3.5 3.0 2.5 2.0 1.5 1.0 0.5 0.0
chemical shift (ppm)

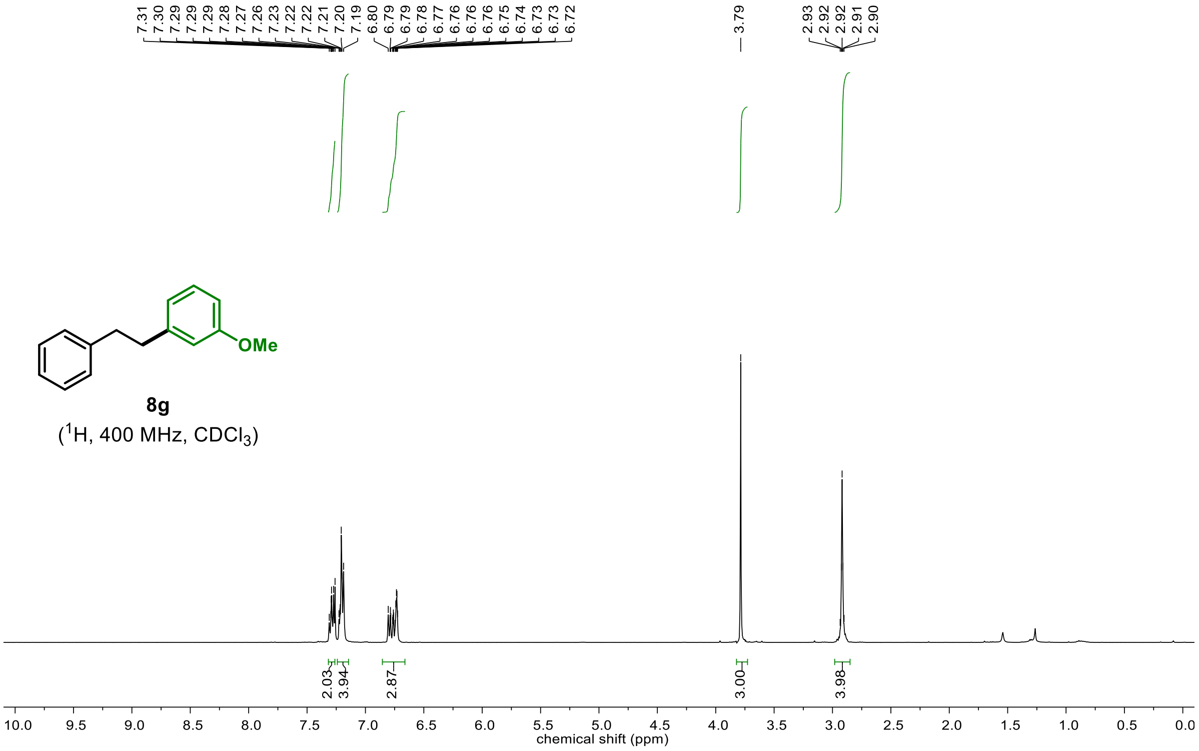
7.31 7.30 7.29 7.29 7.29 7.28 7.27 7.26 7.23 7.22 7.22 7.21 7.20 7.19 6.80 6.79 6.79 6.78 6.77 6.76 6.76 6.76 6.75 6.74 6.73 6.73 6.72
3.79
2.93 2.92 2.92 2.91 2.90
OMe
8g
(^{1}H, 400 MHz, CDCl$_3$)
2.03 3.94 2.87
3.00
3.98
10.0 9.5 9.0 8.5 8.0 7.5 7.0 6.5 6.0 5.5 5.0 4.5 4.0 3.5 3.0 2.5 2.0 1.5 1.0 0.5 0.0
chemical shift (ppm)

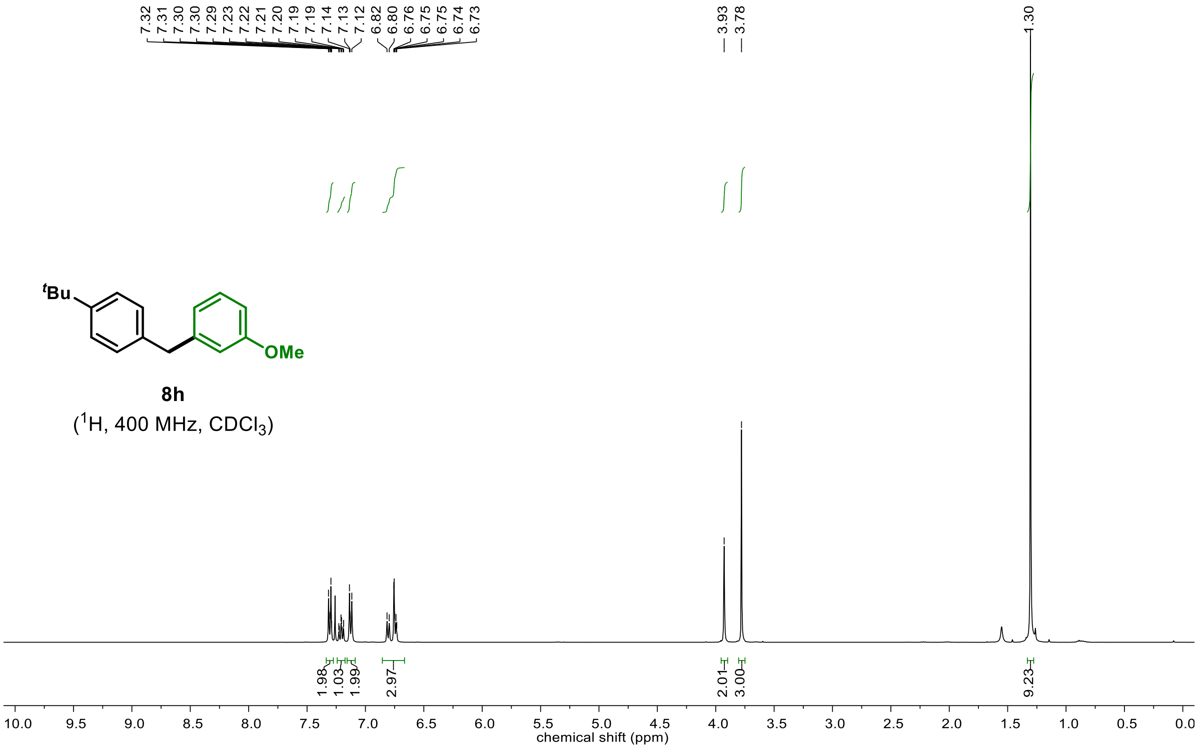
tBu
OMe
8h
(1H, 400 MHz, CDCl3)
chemical shift (ppm)

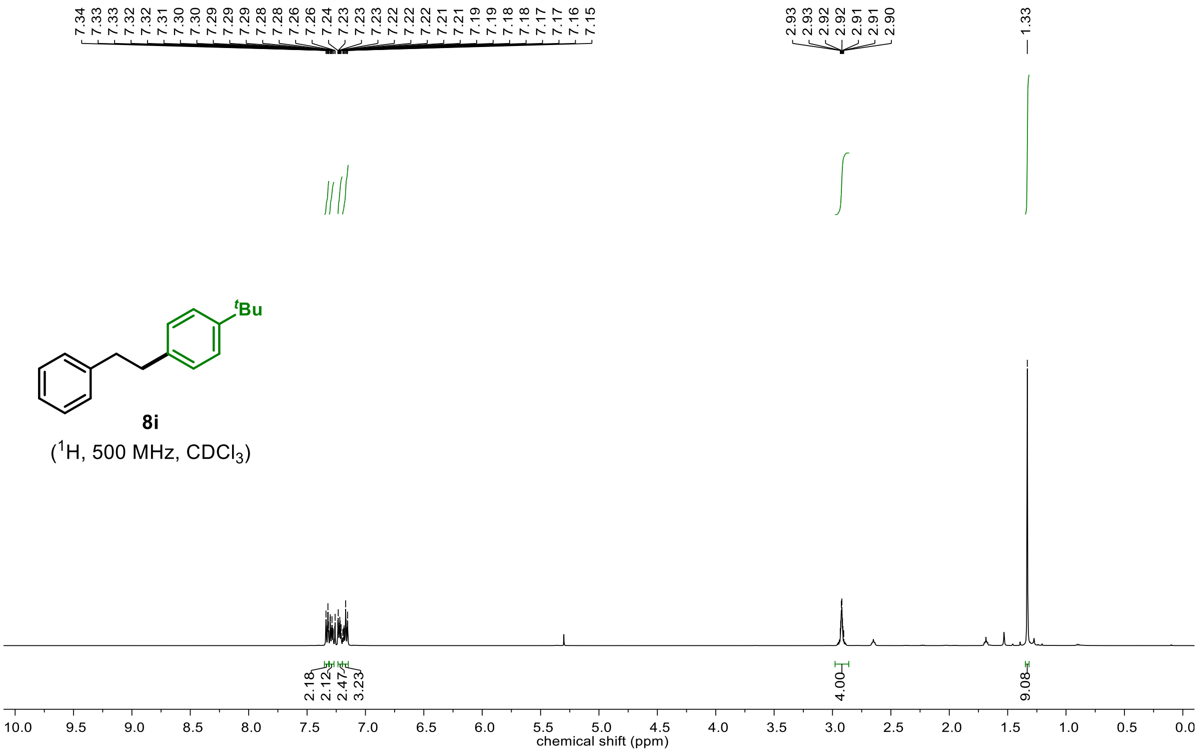
tBu
8i
(1H, 500 MHz, CDCl3)
chemical shift (ppm)
